# Genome, Functional Gene Annotation, and Nuclear Transformation of the Heterokont Oleaginous Alga *Nannochloropsis oceanica* CCMP1779

**DOI:** 10.1371/journal.pgen.1003064

**Published:** 2012-11-15

**Authors:** Astrid Vieler, Guangxi Wu, Chia-Hong Tsai, Blair Bullard, Adam J. Cornish, Christopher Harvey, Ida-Barbara Reca, Chelsea Thornburg, Rujira Achawanantakun, Christopher J. Buehl, Michael S. Campbell, David Cavalier, Kevin L. Childs, Teresa J. Clark, Rahul Deshpande, Erika Erickson, Ann Armenia Ferguson, Witawas Handee, Que Kong, Xiaobo Li, Bensheng Liu, Steven Lundback, Cheng Peng, Rebecca L. Roston, Sanjaya, Jeffrey P. Simpson, Allan TerBush, Jaruswan Warakanont, Simone Zäuner, Eva M. Farre, Eric L. Hegg, Ning Jiang, Min-Hao Kuo, Yan Lu, Krishna K. Niyogi, John Ohlrogge, Katherine W. Osteryoung, Yair Shachar-Hill, Barbara B. Sears, Yanni Sun, Hideki Takahashi, Mark Yandell, Shin-Han Shiu, Christoph Benning

**Affiliations:** 1 Department of Biochemistry and Molecular Biology, Michigan State University, East Lansing, Michigan, United States of America; 2 Cell and Molecular Biology Program, Michigan State University, East Lansing, Michigan, United States of America; 3 Department of Plant Biology, Michigan State University, East Lansing, Michigan, United States of America; 4 DOE–Plant Research Laboratory, Michigan State University, East Lansing, Michigan, United States of America; 5 Deptartment of Computer Science and Engineering, Michigan State University, East Lansing, Michigan, United States of America; 6 Department of Human Genetics, University of Utah, Salt Lake City, Utah, United States of America; 7 Department of Biological Sciences, Western Michigan University, Kalamazoo, Michigan, United States of America; 8 Howard Hughes Medical Institute, Department of Plant and Microbial Biology, University of California Berkeley, Berkeley, California, United States of America; 9 Physical Biosciences Division, Lawrence Berkeley National Laboratory, Berkeley, California, United States of America; 10 Department of Horticulture, Michigan State University, East Lansing, Michigan, United States of America; Rutgers University, United States of America

## Abstract

Unicellular marine algae have promise for providing sustainable and scalable biofuel feedstocks, although no single species has emerged as a preferred organism. Moreover, adequate molecular and genetic resources prerequisite for the rational engineering of marine algal feedstocks are lacking for most candidate species. Heterokonts of the genus Nannochloropsis naturally have high cellular oil content and are already in use for industrial production of high-value lipid products. First success in applying reverse genetics by targeted gene replacement makes *Nannochloropsis oceanica* an attractive model to investigate the cell and molecular biology and biochemistry of this fascinating organism group. Here we present the assembly of the 28.7 Mb genome of *N. oceanica* CCMP1779. RNA sequencing data from nitrogen-replete and nitrogen-depleted growth conditions support a total of 11,973 genes, of which in addition to automatic annotation some were manually inspected to predict the biochemical repertoire for this organism. Among others, more than 100 genes putatively related to lipid metabolism, 114 predicted transcription factors, and 109 transcriptional regulators were annotated. Comparison of the *N. oceanica* CCMP1779 gene repertoire with the recently published *N. gaditana* genome identified 2,649 genes likely specific to *N. oceanica* CCMP1779. Many of these *N. oceanica*–specific genes have putative orthologs in other species or are supported by transcriptional evidence. However, because similarity-based annotations are limited, functions of most of these species-specific genes remain unknown. Aside from the genome sequence and its analysis, protocols for the transformation of *N. oceanica* CCMP1779 are provided. The availability of genomic and transcriptomic data for *Nannochloropsis oceanica* CCMP1779, along with efficient transformation protocols, provides a blueprint for future detailed gene functional analysis and genetic engineering of Nannochloropsis species by a growing academic community focused on this genus.

## Author Summary

Algae are a highly diverse group of organisms that have become the focus of renewed interest due to their potential for producing biofuel feedstocks, nutraceuticals, and biomaterials. Their high photosynthetic yields and ability to grow in areas unsuitable for agriculture provide a potential sustainable alternative to using traditional agricultural crops for biofuels. Because none of the algae currently in use have a history of domestication, and bioengineering of algae is still in its infancy, there is a need to develop algal strains adapted to cultivation for industrial large-scale production of desired compounds. Model organisms ranging from mice to baker's yeast have been instrumental in providing insights into fundamental biological structures and functions. The algal field needs versatile models to develop a fundamental understanding of photosynthetic production of biomass and valuable compounds in unicellular, marine, oleaginous algal species. To contribute to the development of such an algal model system for basic discovery, we sequenced the genome and two sets of transcriptomes of *N. oceanica* CCMP1779, assembled the genomic sequence, identified putative genes, and began to interpret the function of selected genes. This species was chosen because it is readily transformable with foreign DNA and grows well in culture.

## Introduction

The search for sustainable sources of liquid transportation fuels has led to renewed interest in microalgae as potential feedstocks and rising research activity focused on the basic biology of algae. Microalgae can accumulate large quantities of oils (triacylglycerols) and carbohydrates, particularly when nutrient-deprived [1], [2]. Recent estimates taking into account different locations predict that microalgal photosynthesis can produce between 40,000 and 50,000 L ha^−1^ year^−1^, which is 5-to-6 times the yield observed for oil palm [3]. To realize this potential, it will be necessary to understand photosynthetic growth and metabolism of specific model algae. Even though genomic information and basic molecular tools are available for a range of organisms such as the diatoms *Phaeodactylum tricornutum* [4], [5], the brown algae *Ectocarpus siliculosus* [6] or the tiny chlorophyte *Ostreococcus tauri* [7], the mechanistic study of microalgal gene functions is currently lagging behind models such as Arabidopsis. Of all algae, *Chlamydomonas reinhardtii* is currently the most thoroughly studied based on the number of entries in the Public Library of Medicine (http://www.ncbi.nlm.nih.gov/pubmed/). Despite its proven versatility, Chlamydomonas is still somewhat limited with regard to available tools for its molecular analysis. For example, efficient targeted inactivation of genes by gene disruption technology is not available and loss-of-function mutants can be difficult to obtain by RNA interference and related techniques. The recent achievement of homologous gene replacement in *Nannochloropsis oceanica* [8] opens up potential opportunities to develop this alga into an alternate model organism representing marine, oleaginous microalgae.

Nannochloropsis is classified under the class Eustigmatophyceae of the Heterokontophyta [9], a diverse algal group that includes brown algae and diatoms. The plastid of this alga is surrounded by four membranes derived from a secondary endosymbiotic event [10]. Strains from this genus have been investigated for their lipid composition and lipid accumulation, e.g. [11]–[14]. In addition, the biomass production by strains of Nannochloropsis grown under different conditions has been increasingly studied in recent years, e.g. [15]–[19]. Given the potential of this alga as an industrial feedstock and the progress made in developing homologous gene replacement, several research groups have set out to sequence the genome of different Nannochloropsis strains and draft genomes of *Nannochloropsis oceanica* [20] and *Nannochloropsis gaditana* [21] have recently become available.

Here we focus on the publicly available strain *Nannochloropsis oceanica* CCMP1779, which we chose based on its growth in culture, its sensitivity to antibiotics, and ease of integrating transformation markers into its nuclear genome. We sequenced its genomic DNA and two sets of cDNAs obtained from two different growth conditions to aid in the annotation of genes. Its genome has been tentatively compared to that of *N. gaditana*. In addition a team of scientists has begun to manually annotate and examine the gene repertoire for specific pathways and processes to better understand the biology of this alga.

## Results/Discussion

### Strain selection—antibiotic sensitivity, growth and introduction of selectable markers

**Figure 1:**
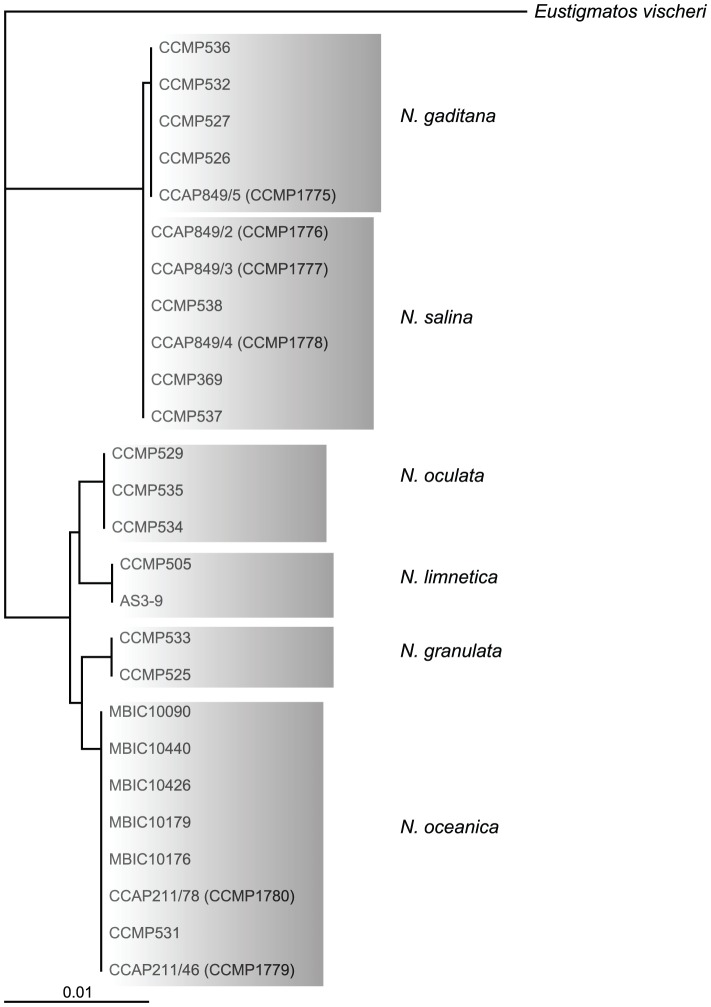
Rooted neighbor joining tree of 18s rRNA sequences of different Nannochloropsis species using *Eustigmatos vischeri* as an outgroup. Labels refer to strain identification numbers from the respective culture collections, if applicable the synonym is given as 2^nd^ name. CCMP, Provasoli Guillard Culture Collection for Marine Phytoplankton, USA; CCAP, Culture Collection of Algae and Protozoa, UK; MBIC, Marine Biotechnology Institute Culture Collection, Japan, AS3-9 from [177].

Out of 20 axenic Nannochloropsis strains obtained from the Provasoli-Guillard National Center for Marine Algae and Microbiota (NCMA, formerly CCMP), strains of the *N. salina* (CCMP369), *N. gaditana* (CCMP1775 and 536) and *N. granulata* (CCMP529), as well as two not further specified strains (CCMP1779 and CCMP531) were selected based on uniformly dispersed, robust growth in enriched artificial sea water (16 g/L marine salt content) in batch culture as well as on agar-solidified medium. Both unspecified *Nannochloropsis sp.* strains cluster with strains of the *N. oceanica* species in a rooted tree [22] based on 26 published 18S rRNA nucleotide sequences (Figure 1) using *Eustigmatos vischeri* (Eustigmatophyceae) as an out-group [23]. For this reason, these strains are hereafter referred to as *N. oceanica*. Because of poor growth under the conditions we have used, *N. oculata* and the fresh water species *N. limnetica* were not further analyzed.

The use of antibiotics is essential for eliminating contaminants from cultures and genes conferring resistance to antibiotics are frequently used as markers for the introduction and genomic insertion of foreign DNA. Therefore, we tested the Nannochloropsis strains for their sensitivity to a range of antibiotics. Cells were plated at high density on agar-solidified medium containing the antibiotics at high density to determine the appropriate dosage (Table S1). Zeocin (5 µg/mL), and Hygromycin B (25 µg/mL) were chosen for use in subsequent selection marker studies, because of the consistent inhibition of growth at low concentrations by these antibiotics. Sensitivity to Paromomycin and Hygromycin B varied among the Nannochloropsis strains; Paromomycin had promise as a selective agent for the two *N. oceanica* strains (CCMP1779 and CCMP531), which were also the most sensitive to Hygromycin B. Of those four antibiotics, plasmids with genes that confer resistance to Zeocin, Hygromycin B, or Paromomycin are readily available and commonly used for transformation of Chlamydomonas as reviewed in [24]. Sensitivity to antibiotics is often determined by its rate of entry into the respective cells, which may be determined by the cell membrane and its transporters and the physical barrier provided by the cell wall. Differences in cell wall composition or thickness allowing more efficient cell entry of antibiotics are possible explanations for increased sensitivity in *N. oceanica* strains. Since efficient uptake of antibiotics or other supplemented molecules (such as metabolic substrates, inhibitors or nucleic acids) is a desirable trait for a laboratory model organism, we focused on *N. oceanica*.

All Nannochloropsis strains were resistant to low concentrations of Rifampicin (10 µg/mL), Benomyl (5 µg/mL), Nystatin (5 µg/mL), and higher concentrations of Spectinomycin (100 µg/ml), Ampicillin (200 µg/ml), and Chloramphenicol (100 µg/mL). Hence these antibiotics can be useful for selecting against bacterial and other possible contaminants in Nannochloropsis cultures.

Basic growth characteristics of *N. oceanica* CMP1779 were determined. The growth curves were fitted to a sigmoidal curve and the averaged exponential growth rate k, maximum cell density a_max_ and time of half maximum cell density x_c_ were determined (Table S2). Under photoautotrophic conditions in enriched sea water the exponential growth rate of the population, k, reached an average of 0.66±0.17 d^−1^ and cultures grew to a cell density of approximately 6×10^7^ cells mL^−1^ (a_max_). The addition of vitamins did not enhance growth in liquid culture, whereas the addition of an external carbon source drastically increased final cell densities in stationary phase, reaching up to 8.7×10^7^ or 1.5×10^8^ cells mL^−1^ when the medium was supplemented with 30 mM glucose or fructose, respectively. The intrinsic growth rate did not increase, indicating a positive effect of sugars on cell division only during the later log phase and/or early stationary phase when self-shading limited growth in the photoautotrophic culture.

Introduction of foreign DNA and stable integration into the genome are crucial for many reverse-genetics approaches. Recently, efficient protocols using an electroporation approach have been published for *N. oceanica* sp. and *N. gaditana* [8], [21]. We tested the strain CCMP1779 for nuclear transformation using an endogenous promoter region of a structural lipid droplet surface protein [25] driving the *aphVII* gene that confers resistance to Hygromycin B. Transformation was performed by electroporation without prior enzymatic treatments [26], and selection on 50 µg/mL Hygromycin B resulted in a transformation rate of 1.25×10^−06^±0.6×10^−06^ per µg plasmid DNA (Table S3). This equals a more than 10-fold increase in transformation events compared to plasmid pHyg3 [27] that was engineered for *C. reinhardtii*. The insertion of the transgene into the genome was confirmed for selected clones of both constructs by Southern hybridization (Figure S1).

### Genome sequencing strategy, assembly, and annotation

**Figure 2:**
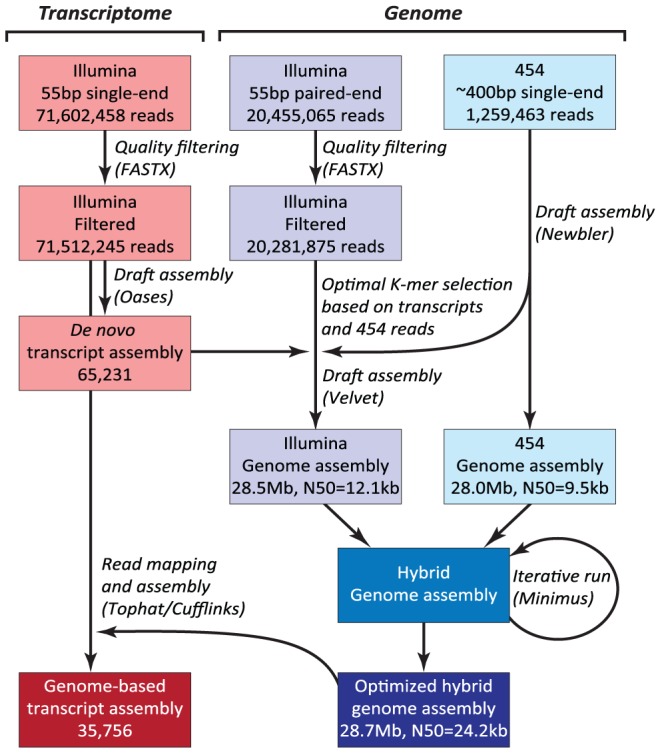
Hybrid assembly strategy using Illumina and 454 reads. N50: the length N for which 50% of all bases in the sequences are in a sequence of length L<N. Kb: kilobase.

The *N. oceanica* CCMP1779 genome was sequenced with 454 and Illumina technology. Both types of reads were used to generate a hybrid assembly with 3,731 contigs, an assembly size of 28.7 Mb and an N50 of 24,152 bp (see Materials and Methods; Figure 2, NCBI/SRA SRP013753). The coverage of the hybrid assembly was calculated to be ∼116-fold (30-fold for 454, and 86-fold for Illumina data). In addition to genomic sequences, we conducted RNA-sequencing (RNA-seq) and generated a *de novo* assembly of 65,321 transcripts. Using these transcripts, we assessed the parameter choice for genome assembly (see Materials and Methods). RNA-seq reads were also mapped to the final genome assembly and assembled into 35,756 transcripts to facilitate structural annotation.

Genome annotation was carried out using the MAKER pipeline [28]. In addition to *ab initio* gene predictions, transcripts from RNA-seq and protein sequences from six other heterokonts (see Materials and Methods for species) were incorporated to generate a draft gene annotation with evidence-based quality values (AED, Annotation Edit Distance) [29]. Basic information about predicted genes and the genome is shown in Table 1. The final annotation set contains 11,973 protein-coding genes: 6,362 gene models with transcript and/or protein similarity support and an additional 5,611 *ab initio* predictions (NCBI/GEO GSE36959). Protein domain search results showed that the percentage of proteins with InterPro domains in CCMP1779 is comparable to but slightly lower than that of the other six sequenced heterokonts (Figure S2C, Table S24). We also found 83.4% of the proteins from the CEGMA database that contain highly conserved eukaryotic proteins [30]. For comparison, the representation of CEGMA proteins in the green alga Chlamydomonas, the parasitic protozoan *Toxoplasma gondii*, and the heterokont *Ectocarpus siliculosus* are 88.9%, 66.2% [30], and 85.8% [31], respectively. These findings demonstrate that our annotation is of similar quality as that for the other eukaryotes, particularly heterokont genome annotations.

**Table 1:** Genome summary.

Feature	Value
Assembly size	28.7 Mbp
G+C content	53.8%
Protein coding genes	11,973
Average gene size	1,547 bp
Average exons per gene	2.7
Average introns per gene	1.7
Average length of exons	417 bp
Average length of introns	230 bp

### Functional annotation based on protein domains, functional category assignments, and expression

**Figure 3:**
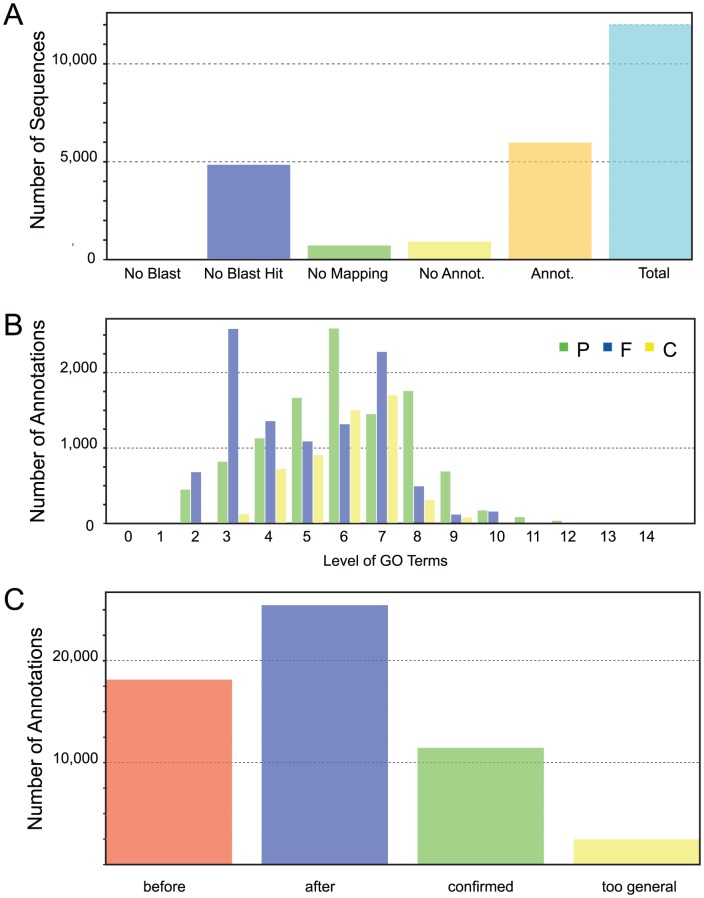
Gene Ontology. (A) Blast2Go functional annotation results overview. No Blast, Number of sequences without blast search performed; No Blast Hit, Number of sequences without blastp hits at the given threshold (e-value<10^−4^); No Mapping, Number of blast hits that did not map to the Blast2GO database; No Annot., Number of mapped hits that did not retrieve GO annotations from the Blast2GO database; Annot., Number of sequences that did retrieve one or more GO annotations from the Blast2GO database; Total, Total number of analyzed sequences. (B) The distribution of GO annotations by GO level shows the respective number of added GO annotations in relation to their GO level for each category (P biological process, F molecular function, C cellular component). (C) Results distribution after implementation of InterProScan results. Before, Total number of added GO terms after Blast2GO annotation; after, Total number of GO annotations after implementation of InterProScan results; confirmed, Number of initial GO annotations confirmed by InterProScan result; too general, Number of GO annotations removed after InterProScan because of a lack of specificity.

To generate functional annotation, we first identified protein domains in annotated genes. Of the 12,012 identified protein models in our first annotation run, 4,847 did not have a significant match in the NCBI (http://www.ncbi.nlm.nih.gov) non-redundant protein database (version 4, January 20, 2012). One potential explanation for this relatively high number of putatively unique genes is that related sequences are not annotated in heterokonts. In addition, we cannot rule out the possibility of false positive gene prediction. Of the 7,165 (59.6%) protein models with matches, 721 protein sequences could not be mapped by Blast2GO [32] to retrieve GO (Gene Ontology) terms and annotation to select reliable functions. Manual examination of a random selection of these proteins revealed that they matched mostly uncharacterized proteins, usually from other heterokont genomes such as *E. siliculosus* or *Albugo laibachii*. A total of 26,573 GO terms were assigned after augmented annex annotation [33] and merging primary GO annotations with the InterPro Scan results [34] (Figure 3). A total of 5,981 (49.8%) CCMP1779 genes had GO annotations.

Our RNA-seq runs were conducted with RNA samples obtained from cells grown under nitrogen (N)-replete and N-deprived conditions that typically differ in the biosynthesis of storage lipids among other metabolic functions, (see e.g. [35]). To assess whether expression of genes in certain functional categories were particularly influenced by these conditions, we determined the enrichment of GO terms in up- and down-regulated genes. At 1% significance level (Fisher's Exact Test), genes with 7 and 27 GO terms were significantly enriched in up- and down-regulated genes, respectively (Table S4). In particular, genes associated with photosynthesis and DNA replication tended to be down-regulated following N deprivation, but also genes for central carbon metabolism were affected, such as gluconeogenesis and glycolysis. We previously observed similar effects for Chlamydomonas [35] which is evolutionarily distant from Nannochloropsis.

### Comparison of *N. oceanica* and *N. gaditana* gene sets

Recently the genome sequence of a related species, *N. gaditana* (*Ng*) has become available providing an opportunity for direct comparison. It was reported that 2,733 genes (30.2% of the total gene models) in the *Ng* genome were exclusive to the species compared to *E. siliculosus* and other distantly related algae [21]. The assembly sizes were ∼28.7 Mb for *N. oceanica* CCMP1779 (*No*) and ∼29 Mb for *Ng* with a larger protein number identified in *No* (12,012) compared to *Ng* (9,053). To identify unique and conserved gene repertories between the two *Nannochloropsis* species, we first compiled annotated protein coding sequences from both as well as *E. siliculosus* and defined orthologous groups (OGs).

An OG contains a group of genes that were descendants of a single ancestral gene in the most recent common ancestor of both *Nannochloropsis* species. Among 6,395 OGs identified, 5,048 OGs contain genes from both *Nannochloropsis* species. These “shared” OGs contain 5,324 *No* and 5,251 *Ng* genes, respectively (Table S5). On the other hand, 6,688 *No* and 3,802 *Ng* genes are in single species OGs (which is indicative of gene loss in the other species lineage) or are singleton genes. To evaluate if any of the presumptive *No*-specific genes had a match in the *Ng* genome and, thus, were not truly species-specific, a similarity search was carried out using *No* protein sequences against *Ng* genome sequences. Of the 6,688 presumptive *No*-specific genes, 2,394 had ≥1 significant matches (see Materials and Methods) to the *Ng* genome, while 4,294 remain *No*-specific (Table S5). Among 3,802 presumptive *Ng*-specific genes, 1,153 of them have ≥1 significant matches to the *No* genome and 2,649 remain *Ng*-specific (Table S5).

Some of these species-specific genes may be relevant to biological differences between the two species, perhaps related to their distinct life histories. However, they could also be false positive predictions. Using three lines of evidence, we show that some of these species-specific genes are likely authentic. The first is through examining their Annotation Edit Distance (AED), a score that reflects the annotation quality with a range between 0 (perfect match to similar sequences or transcript evidence) and 1 (no match) [29]. The AED distributions of *No* genes in conserved OGs and those that are species-specific are shown in Figure S3. Here conserved OGs refer to OGs with the same number of genes from both *Nannochloropsis* species. Genes in conserved OGs have an average AED score of 0.35, significantly lower than that of species-specific genes (0.73, Kolmogorov-Smirnov Test, *p*<2.2e-16). Given an AED closer to 1 indicating diminishing support, species-specific genes generally have less support based on similarity or transcript evidence compared to conserved genes. Nonetheless, 34.8% of *No*-specific genes have AED<0.5, indicating 50% of the annotated regions overlap with ≥1 similar sequences and/or transcripts. Thus, some of these species-specific genes are likely not spurious.

The second line of evidence is that a number of *No*-specific and *Ng*-specific genes have putative *E. siliculosus* orthologs. Among 1,040 OGs without *Ng* gene, 863 contain both *No* and *E. siliculosus* genes. Similarly, in 307 OGs without *No* gene, 236 have genes from both *Ng* and *E. siliculosus*. These findings indicate that a number of species-specific genes are authentic and the reason they are species-specific is most likely due to gene loss and/or missing annotation in one of the *Nannochloropsis* species. The third line of evidence is that 1,086 *No*- and 253 *Ng*-specific genes have a significant match to annotated proteins from other species that can be used for functional category annotation based on sequence similarity (see previous section on Blast2GO).

We conducted enrichment tests to examine which functional categories tend to be associated with conserved genes or species-specific genes. Here conserved genes are defined as genes that reside in OGs with the same number of genes from both *Nannochloropsis* species. Species-specific genes on the other hand are defined as annotated genes from one species that do not have a protein or genomic match from the second species. We found that conserved genes, as expected, are involved in essential processes including translation, ribosome biogenesis, photosynthesis, and central metabolism (Table S6). For species-specific genes, we also identified multiple enriched categories (Table S6). However, the degree of enrichment is rather marginal and the test statistics are not particularly robust. This is most likely because there is extremely limited knowledge of gene functions among Heterokont species. One noteworthy enriched GO category (acetyl-CoA carboxylase activity) may reflect subtle differences in fatty acid biosynthesis, which is relevant for the use of the respective organism for the production of biofuel feedstock.

### Repetitive sequences

Approximately 10% of the assembled genome is composed of repetitive sequences. The majority of them (8.7% of the genome) are low complexity or simple repeats. Only 1.4% of the assembled sequence is composed of interspersed repetitive sequences, and half of these are recognizable transposable elements. This is likely an underestimate of their occurrence in the genome, due to the collapsing of contigs with repetitive elements during genome assembly. Despite the low abundance, transposons in CCMP1779 are rather diverse including distinct elements with similarity to those in animals, plants and other algae. Among the recognizable transposable elements, DNA transposons are the predominant type, with *Helitron*s being the most abundant elements in the genome (Table 2). In contrast, there are few (17) retrotransposons, and no intact element was detected. While we cannot rule out the possibility that the lack of intact copies is an artifact of assembly, it is clear that the copy number of retrotransposons is relatively low and there is no indication of recent activity of retrotransposons. This is distinct from the composition of most plant repeats where LTR retrotransposons are the most abundant repetitive sequences and often contribute significantly to genome size expansion [36], [37].

**Table 2:** Occurrence of repetitive sequences in the CCMP1779 genome.

	Repeat	Copy number*	Length occupied (bps)	Genome fraction (%)
Interspersed repeats	LINE	2	659	0.00
	LTR elements	17	7863	0.03
	DNA elements	736	194338	0.68
	Unclassified	1270	214449	0.75
Local repeats	Simple repeats	–	1149204	4.00
	Low complexity	–	1353066	4.71

* Copy number includes truncated elements and fragments, LINE long interspersed nuclear elements, LTR long terminal repeat.

### Non-coding RNAs

In addition to protein coding genes, there are a substantial number of putative non-coding RNA (ncRNA) genes in CCMP1779. These ncRNA genes were identified by first searching for sequences similar to annotated ncRNA families from Rfam [38] with Infernal [39] using the gathering cutoff score threshold. In the second step, ncRNA predictions were excluded from further analysis if they overlapped with annotated exons or repetitive regions soft-masked by RepeatMasker [40]. After these two steps, 6300 putative ncRNA genes remained, of which 5931 were putative microRNAs (miRNAs). To further reduce the number of false-positive predictions, we examined whether these putative miRNAs have readily identifiable potential targets within the CCMP1779 genome. Assuming that the regulatory targets will have at least partial sequence identities to true miRNAs, RNA-seq data generated for cells grown under N-replete and N-depleted conditions were combined to search for miRNA targets with FindMiRNA [41]. After removing all putative miRNAs that lack potential target genes and consolidating overlapping predictions, 101 putative miRNA genes were identified. It is possible that this approach would lead to false negatives because not all genes are transcribed under the conditions we examined. Together with 125 tRNAs, snoRNAs, and other types of ncRNAs, 226 ncRNA genes were predicted with high confidence (Table 3).

**Table 3:** Distribution of putative non-coding RNAs in the CCMP1779 genome.

	Proportion of ncRNA	Number of Records
miRNA	44.69%	101
snoRNA	33.63%	76
tRNA	16.81%	38
rRNA	2.21%	5
others	2.65%	6

### Metabolism: Photosynthesis

Photosynthesis is an essential physiological process in *N. oceanica*, which, as an obligate photoautotroph, must be able to harness light energy for metabolism and growth. Proteins involved in the light reactions of photosynthesis are encoded by both the nuclear and plastid genomes in eukaryotic algae. We have identified several nuclear genes that encode components of the photosynthetic linear electron transport chain, including components of the photosystem (PS) I reaction center, the PSII reaction center, the cytochrome b_6_/f complex, ATP synthase, and electron carriers (Table S7). In particular, CCMP1779 contains *ATPD, PETM*, and *PSBX* genes in the nucleus, in contrast to other heterokont algae, in which these genes are found in the plastid genome.

**Figure 4:**
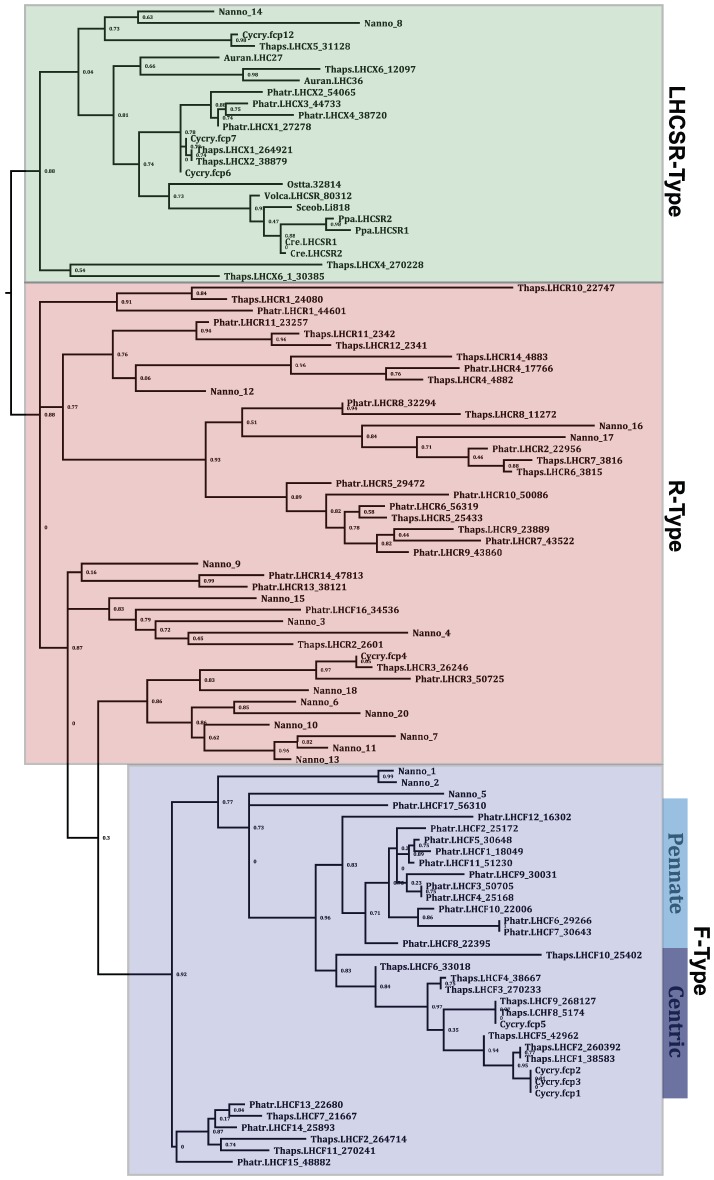
Maximum likelihood analysis of a MUSCLE alignment of 19 of the identified putative VCP protein sequences from *Nannochloropsis oceanica* CCMP1779 (Nanno) and the annotated LHC and LHCSR-like sequences from *Phaeodactylum tricornutum* (Phatr), *Cyclotella cryptica* (Cycry), *Thalassiosira pseudonana* (Thaps), *Aureococcus anophagefferens* (Auran), and the LHCSR-like sequences from *Chlamydomonas reinhardtii* (Cre), *Ostreococcus tauri* (Ossta), *Physcomitrella patens* (Ppa), *Scenedesmus obliquus* (Sceob), and *Volvox carteri* (Volca). *Nannochloropsis* VCP model 19 (Nanno_19) was not included in the analysis because the sequence is incomplete, causing it to be erroneously assigned as an out-group.

Collection of light energy for conversion to chemical energy is performed by light-harvesting complexes found in the thylakoid membrane of the plastid. The most abundant of these pigment-binding antenna proteins are part of the light-harvesting complex (LHC) superfamily of proteins [42]. Analysis of genes encoding proteins homologous to LHC proteins shows that CCMP 1779 has genes for at least 19 members of the LHC superfamily. These members belong to three distinct clades of LHC proteins (Figure 4, Table S8): one group related to the major fucoxanthin-chlorophyll protein (FCP)-like LHCs or LHCFs of diatoms [43], a second group related to the red-algal-like LHCs known as LHCRs [44] and a third group of stress-responsive LHC proteins known as LHCSRs in green algae and bryophytes [45], [46] and LHCXs in diatoms [47]. However, no genes encoding the PSBS protein were identified, which is essential for the photoprotective qE component of non-photochemical quenching (NPQ) in plants [48] and contributes to qE in bryophytes [46].

Nannochloropsis makes chlorophyll *a* but lacks an accessory chlorophyll, and it produces predominantly violaxanthin and vaucheriaxanthin-esters as the major light-harvesting accessory carotenoids pigments, which are associated with the antenna proteins [49] (Table S9). Genes homologous to carotenoid biosynthetic genes of land plants and green algae are present in the CCMP1779 genome, except for a clear ortholog of carotene isomerase (Table S9). Consistent with the exclusive presence of β-xanthophylls, only a single lycopene β-cyclase gene was found, and unlike plants, only a single carotene hydroxylase gene (of the cytochrome P450 type rather than a di-iron hydroxylase) is present (Table S9).

As described by Sukenik et al. [50], the LHC superfamily proteins in Nannochloropsis are referred to as violaxanthin-chlorophyll proteins (VCPs). CCMP1779 contains a protein homolog with 96% identity and 95% coverage at the protein level of the VCP protein of another Nannochloropsis strain [50]. A homolog of the VCP gene has also been described recently by Kilian et al. [8] in another Nannochloropsis isolate, and called VCP1. This group also identified a second LHC gene called VCP2. We identified homologs of these genes in CCMP1779, which have 99% and 100% identity, respectively, at the nucleotide sequence level.

Based on sequence similarity, there are also members of the LHC superfamily in CCMP1779 that might function in photoprotection as opposed to light harvesting. Two of the identified putative VCP proteins have higher similarity to LHCSR and LHCX protein than the other LHC types, and we hypothesize that they function in qE.

CCMP1779 has a gene encoding a highly conserved violaxanthin de-epoxidase (VDE) protein like that found in plants (Table S9). In Arabidopsis, VDE is responsible for the conversion of violaxanthin to antheraxanthin and zeaxanthin, in a process known as the xanthophyll cycle. Violaxanthin and zeaxanthin have a well-established role as pigment ligands for plant LHCII complexes [51] and as quenchers of triplet chlorophyll and singlet oxygen in the thylakoid membrane [52]. Furthermore, zeaxanthin and/or antheraxanthin are necessary for maximum qE *in vivo* in Arabidopsis [53]. Nannochloropsis has been shown to utilize the xanthophyll cycle in high light, and the activity appears to be somewhat dependent on temperature acclimation of the cells [54].

### Metabolism: Carbon fixation

The photosynthesis of aquatic microorganisms accounts for approximately 50% of global carbon fixation [55]. Microalgae are adapted to limited and fluctuating inorganic carbon (C_i_) sources in their environment and employ carbon concentrating mechanisms (CCMs), which locally enhances the intracellular C_i_ concentration and thereby the rate of photosynthetic CO_2_ fixation. In Chlamydomonas, C_i_ transporters, carbonic anhydrases and various regulatory genes have been identified as recently reviewed in [56]. We used the information available for the CCM in Chlamydomonas to identify orthologous genes in CCMP1779. In Chlamydomonas, at least nine carbonic anhydrases have been identified [57] with different subcellular localizations. These carbonic anhydrases are divided into two groups, α-type and β-type. The α-type carbonic anhydrases are similar to mammalian carbonic anhydrases, while the β-type is more similar to plant carbonic anhydrases. Only two putative carbonic anhydrase encoding genes were identified in the CCMP1779 genome, one of them an α-type and the other a β-type (Table S10). Notably, in a recently published genome annotation, six putative carbonic anhydrases were identified in *N. gaditana* [21]. Even though we cannot rule out differences in assembly and annotation procedures as a possible cause for this observation, this seems to be an apparent difference between the two species possibly reflecting a better adaptation of *N. gaditana* to lower C_i_ concentrations in the environment. C_i_-transport across membranes in Chlamydomonas is mediated by HLA3 [58] and LCI1 [59], two C_i_ transporters in the plasma membrane, and LCIA [60] and CCP [61] located in plastid membranes. In the CCMP1779 genome we identified several genes that resemble the LCIA and CCP encoding genes of Chlamydomonas, but no putative orthologs of HLA3 or LCI1 were present. This result suggests that CCMP1779 might have a plastid C_i_ transport system similar to that of Chlamydomonas, but a distinct mechanism for uptake of C_i_ at the plasma membrane.

The marine unicellular diatom *Thalassiosira weissflogii* was shown to have the enzyme repertoire to possibly conduct C4 photosynthesis [62] and key enzymes required for C4 photosynthesis were biochemically identified or their genes were annotated for several diatom species [63], [64]. Like diatoms, Nannochloropsis contains a red algal plastid acquired by secondary endosymbiosis during heterokont evolution [65] and therefore is similar with regard to the intracellular membrane system. For *N. gaditana*, C4-type carbon concentrating mechanisms were reconstructed *in silico*, based on the predictions of protein localization [21]. In CCMP1779, most of genes presumably involved in a C4 pathway were identified, including phosphoenolpyruvate carboxylase (PEPCase), phosphate dikinase (PPDK), NAD malic enzymes (ME) and malate dehydrogenase (MHD). However, we were unable to identify a possible ortholog encoding phosphoenolpyruvate carboxykinase (PEPCK) in CCMP1779. Thus it seems possible that the genes putatively encoding enzymes of the C4-pathway may play metabolic roles beyond carbon fixation in CCMP1779, e.g. anapleurotic reactions. Testing of these hypotheses derived from the genome sequence will likely provide insights into alternate carbon concentration pathways that might be targeted for maximizing biomass yield in algae.

All the genes of central metabolism (glycolysis and gluconeogenesis, the TCA cycle, oxidative and reductive pentose phosphate pathway, as well as the glyoxylate cycle) appear to be present in the CCMP1779 genome (Table S10). Many are present in multiple copies indicating that these enzymes may be present in multiple compartments. However there are exceptions. Only a single copy of the TCA cycle genes encoding aconitase and isocitrate dehydrogenase were found and predicted to be mitochondrial. Similarly the pentose phosphate gene encoding ribose-5-phosphate isomerase is present only as a single copy, indicating that this activity is restricted to the plastid. The multiple copies of all the enzymes of the glycolytic pathway indicate this pathway is likely to be active in both the cytosol and plastid.

Nannochloropsis is reported to have an enhanced growth on various carbon sources [66]–[68], which is consistent with the presence of the genes for the full repertoire of central metabolism.

### Metabolism: Hydrogen production

Hydrogen produced by microalgae has long been discussed as a possible sustainable transportation fuel source as electrons derived from photosynthetic water-splitting can be coupled to H_2_ production in green algae and cyanobacteria [69]. Upon examination of the CCMP1779 genome, we discovered a single gene that encodes a putative [FeFe]-hydrogenase (*hydA*) (Table S11). This class of enzymes catalyzes the reversible reduction of protons to molecular hydrogen [70]. In addition, three genes that code for proteins required for hydrogenase maturation (*hydE*, *hydF*, and *hydG*) were located directly up- and downstream of *hydA*. In several H_2_-evolving bacteria, these genes cluster together and often form operons or have an operon-like organization. Interestingly, unlike currently sequenced green algae, in which two of the maturation genes have been fused (*hydEF*) [71], CCMP1779 does not show evidence of this fusion. In the recently reported genome sequence of *N. gaditana* [21], a cluster of hydrogenase and maturation protein orthologs was noted. Clustering of these genes in CCMP1779 may indicate a relatively recent horizontal gene transfer in the organism's evolution, and the absence of the *hydEF* fusion gene hints that acquisition of this hydrogenase gene cluster by CCMP1779 could be distinct from that in green algae.

**Figure 5:**
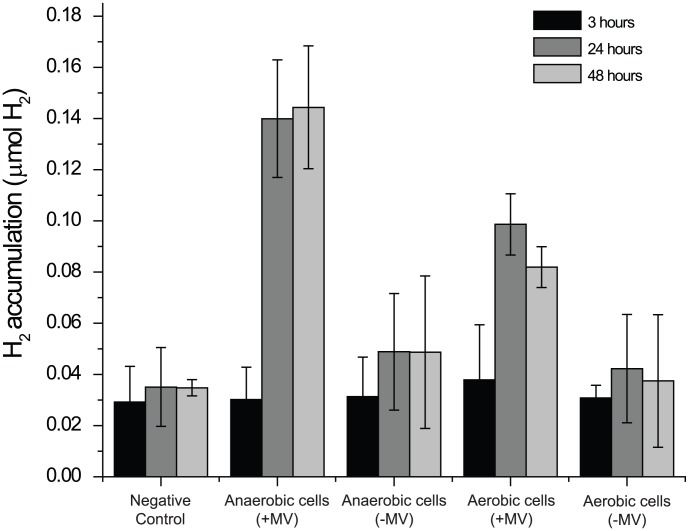
Hydrogen production. H_2_ accumulation was measured at 3, 24, and 48 hours after adding aerobically- or anaerobicially-incubated cells to air-tight sample vials. The vials contained growth media supplemented with 10 mM methyl viologen and 100 mM Na_2_S_2_O_4_ (+MV) or unsupplemented growth media (−MV). A sample without cells was used as a negative control. (n≥3).

Based on the presence of this cluster, anaerobically acclimated CCMP1779 was tested for its ability to produce H_2_ in both the presence and absence of an abiotic electron donor. An accumulation of H_2_ was noted in the headspace only when methyl viologen was supplied (Figure 5). No appreciable increase of H_2_ in the headspace was noted in the absence of the abiotic electron donor relative to the negative control, even 48 hours after initiating the assay. Aerobically-incubated cells accumulated considerably less H_2_ in the headspace, presumably because the proteins involved in H_2_ production are not synthesized until the assay conditions assure anaerobiosis. Together, these data indicate that CCMP1779 contains functional genes for H_2_ metabolism.

### Metabolism: Fatty acid and lipid synthesis

Glycerolipids and specifically triacylglycerols (TAG) are the feedstock for the production of biodiesel from algae. Therefore, one focus here is on genes related to the synthesis and degradation of these lipids in CCMP1779. In general, the glycerolipid compositions of CCMP1779 resembles that of a typical photosynthetic organism, comprised mostly of the prevalent glycoglycerolipids mono- and digalactosyldiacylglycerol (MGDG and DGDG) and sulfoquinovosyldiacylglycerol (SQGD), as well as the common phospholipids phosphatidylcholine (PtdCho), phosphatidylethanolamine (PtdEtn) and phosphatidylglycerol (PtdGro). In addition, the betaine lipid diacylglycerol-O-4′-(*N,N,N*-trimethyl)-homoserine (DGTS) is present [11].

With respect to the proposed use of Nannochloropsis species as a feedstock for biodiesel and nutraceuticals, their high content of TAG and their enrichment in eicosapentaenoic acid (EPA), a polyunsaturated fatty acid (FA) of 20 carbon length containing five double bonds (20∶5), are of particular interest. EPA is mainly found in the membrane lipid fraction, with only traces present in the TAG fraction (Table S12, [72]). In CCMP1779 EPA occurs mostly in MGDG and DGTS (Table S12). A synthesis pathway for EPA was suggested involving the phospholipid pools PtdCho and PtdEtn, with 18 carbon fatty acids (18∶2) accumulating only in PtdCho, and 20 carbon intermediates (20∶4) only in PtdEtn (Table S12, [11]).

**Figure 6:**
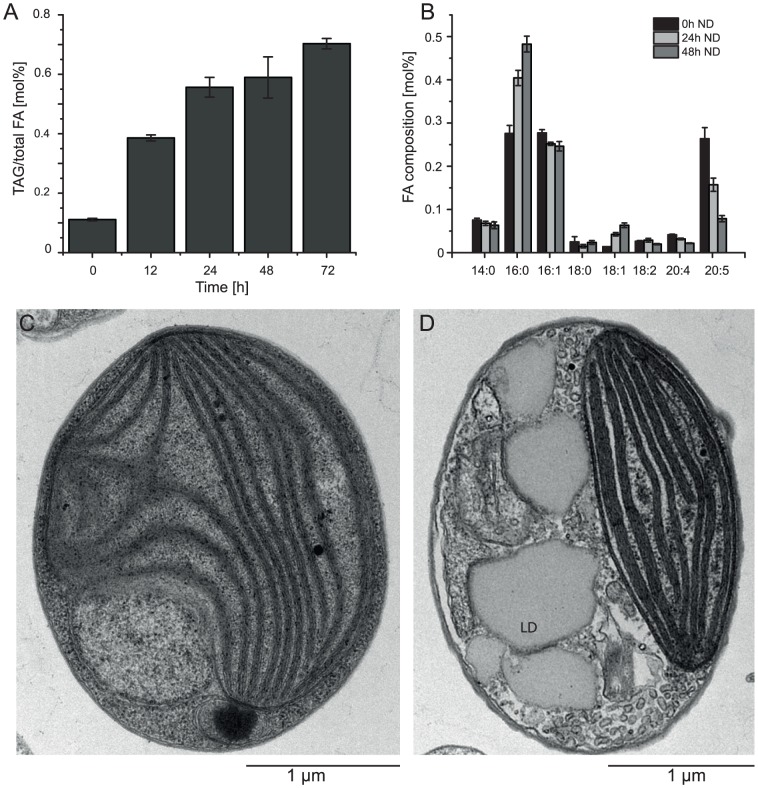
Accumulation of oil. (A) TAG accumulation over time shown as fatty acids esterified to TAG (TAG FA) over total fatty acids (FAtotal) following nitrogen deprivation, and (B) characteristic changes in the fatty acid profile. Fatty acids are designated based on number of carbon atoms: number of double bonds. The accumulation of TAG and the formation of lipid droplets can be observed in ultra-structural changes following nitrogen starvation (C, N-replete; D, N-depleted).

Nitrogen (N) deprivation is commonly used to induce the accumulation of triacylglycerols (TAG) and the formation of lipid droplets [25]. We investigated the basic characteristics of CCMP1779 in terms of TAG accumulation and changes in the fatty acid profile following N deprivation and observed morphological changes associated with lipid droplet formation (Figure 6). Following N deprivation, TAG accumulation increased after approximately 12 h, which is also represented in the decline of EPA content. A maximum of 82% of total fatty acids were associated with TAG after 48 hours of N deprivation. Lipid droplets take up a large proportion of the cell's interior during these conditions compared to N-replete cells, in which the plastid is the most prominent cellular structure (Figure 6C, 6D).

Fatty acid synthases (FAS) can be divided into two classes, type I and II [73], [74]. Type I systems occur as large multi-enzyme complexes on one or two large polypeptide chains and are primarily present in animals and fungi. In contrast, in type II systems the FAS proteins are expressed as individual polypeptides from a series of separate genes. Type II FAS occurs in most bacteria and in organelles (chloroplasts/mitochondria) of plants, animals and algae. We identified all the genes encoding enzymes central to fatty acid biosynthesis in plastids, i.e., components of the multimeric acetyl coenzyme A (acetyl-CoA) carboxylase and type II FAS complexes (Table S13). The monomeric cytosolic counterpart of acetyl-CoA carboxylase is also present in the CCMP1779 genome. Except for 3-hydroxyacyl-ACP (acyl carrier protein) dehydratase and enoyl-ACP-reductase, whether the subcellular localization is in the plastid or mitochondrion is difficult to predict for the remainder of the type II FAS components. A thioesterase candidate gene similar to Arabidopsis FatA or FatB was not present, but an ortholog similar to an *Ectocarpus siliculosus* putative thioesterase was identified.

Interestingly, a presumed homolog to type I FAS encoding genes was identified in CCMP1779 similar to FAS from animals. It should be noted that type I FAS enzymes are mechanistically and structurally similar to polyketide synthases [75], and it is not clear what the role of this putative type I FAS complex might be. However, it is possible that Nannochloropsis has both cytosolic and organellar fatty acid synthesis pathways as observed for Euglena [76]. The putative type I fatty acid synthase is possibly the source of the short-chain saturated fatty acids (C14:0) as proposed for the heterotrophic heterokont protist *Schizochytrium sp.* [77]. *Schizochytrium sp.* contains a multi-subunit polyunsaturated fatty acid (PUFA) synthase and a predicted type I FAS, and produces PUFAs (DHA and DPA) and short-chain saturated fatty acids (C14:0 and C16:0).

In plants and green algae, glycerolipids are synthesized by two distinct pathways associated with the chloroplast or the endoplasmic reticulum (ER), referred to as the prokaryotic and eukaryotic pathway respectively [78]. Glycero-3-phosphate serves as the backbone and the activities of a glycerol-3-phosphate-*sn1*-acylACP-acyltransferase (GPAT) and lysophosphatidic acid acyltransferase (LPAT) successively lead to the formation of phosphatidic acid, a central metabolite in glycerolipid metabolism. In CCMP1779, both the eukaryotic and the prokaryotic pathways are likely to operate (Table S13). Similar to *N. gaditana* [21], we identified a set of nine putative GPAT and LPAT candidates, of which only the chloroplast candidates can be unambiguously assigned based on their predicted localization and protein domain organization. The majority of membrane lipids is synthesized from diacylglycerol (DAG) by the addition of the respective activated headgroup, where the typical plastid membrane lipids, the glycoglycerolipids MGDG, DGDG, and SQDG are assembled at the plastid envelope membranes, and the phospholipids PtdCho and PtdEtn along with the betaine lipid DGTS are likely synthesized at the ER. For all necessary enzymes, the respective genes were tentatively identified (Table S13).

**Figure 7:**
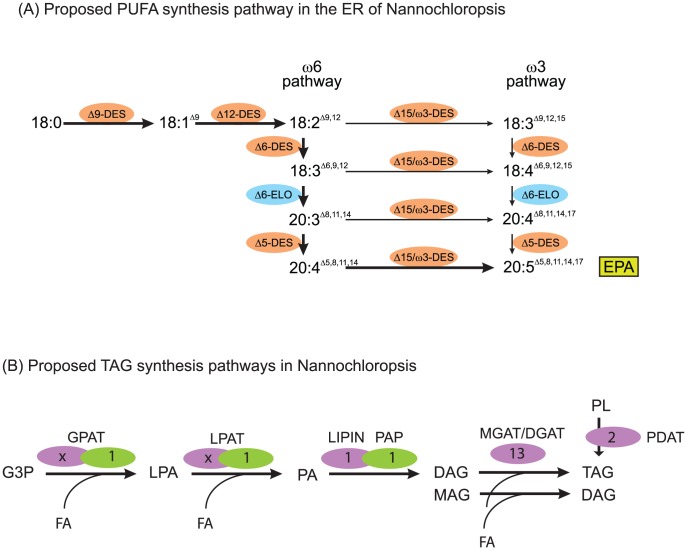
Lipid assembly and modification. (A) Proposed pathway of desaturation and elongation of fatty acyl chains in the ER of Nannochloropsis. EPA, eicosapentaenoic acid (B) Proposed plastid (green) and ER (lilac) pathway and genes putatively involved in the synthesis of TAG in Nannochloropsis. Numbers indicate count of putative genes identified. Number of ER acyltransferases cannot be assigned unambiguously, multiple candidate genes are listed in Table S13. G3P, glycerol-3-phosphate, LPA, lysophosphatidic acid, PA, phosphatidic acid, MAG, monoacylglycerol, DAG, diacylglycerol, TAG, triacylglycerol, PL, polar glycerolipid. GPAT, glycerol-3-phosphate acyltransferase, LPAT, lysophosphatidic acid acyltransferase, PAP, phosphatidic acid phosphatase, LIPIN, Lipin, MGAT/DGAT mono-/diacylglycerolacyltransferase, PDAT, phospholipid-diacylglycerolacyltransferase.

During the assembly of TAG (Figure 7B), which accumulates in specific lipid droplets [25], diacylglcyerol acyltransferase (DGAT) adds a third fatty acid to DAG. Remarkably, a total of 13 putative type 2 DGAT encoding genes were identified in the CCMP1779 genome, out of which only two genes did not have EST support. Only one candidate gene was detected to encode a protein similar to plant type 1 DGAT, containing an MBOAT (membrane bound *O*-acyltransferase) domain. However, there is no EST support for this gene model. Given the generally low redundancy of genes in the relatively small and condensed CCMP1779 genome, this finding seems to support the diversity of TAG metabolism in Nannochloropsis. However, it is impossible to distinguish putative DGATs from monoacylglycerol acyltransferases (MGATs), and the high number of genes for this enzyme class may imply the presence of a monoacylglycerol pathway for TAG synthesis in Nannochloropsis. A third possibility for TAG biosynthesis is the acyl-CoA-independent transfer of a glycerolipid-bound fatty acid to DAG. This pathway is present in most eukaryotes and is performed by a so called phospholipid:DAG acyltransferase (PDAT). We tentatively identified two putative PDAT encoding genes in CCMP1779. Along with the relatively high number of GPAT and LPAT candidates, this likely reflects the complex regulation and control and the importance of TAG metabolism in this algae. A similarly complex set of putative genes for these enzymes has been described for *N. gaditana* [21].

Each of the different glycolipids is characterized by a specific composition of its attached fatty acids (Table S12). In order to synthesize the rather simple set of fatty acids found in CCMP1779, a minimal set of six desaturases and one elongase is required. We tentatively identified all necessary desaturases, and functionally annotated them based on their primary sequence (Figure 7A, Table S13, [79], [80]). Besides genes encoding the soluble plastid acyl-ACP desaturase and the PtdGro specific Δ3*^t^*-desaturase, a complete set of genes encoding membrane-bound ER-localized desaturases, namely putative Δ9-, Δ12-, Δ6-, Δ5- and ω3-desaturases, was identified. This implies the synthesis of EPA occurs exclusively outside of the plastid and is, therefore, in line with the proposed pathway involving PtdCho and PtdEtn [11]. Since the majority of EPA is found inside the plastid esterified to MGDG, this raises interesting questions about the lipid trafficking pathways and, taking into account the enrichment of EPA in DGTS as opposed to PtdCho and PtdEtn, leads to the speculation that the betaine lipid might be involved as a precursor for the formation of MGDG and DGDG inside the plastid. A gene encoding a putative Δ6-elongase was identified based on sequence similarity with characterized elongases [79], out of a total of eleven fatty acid elongase-like genes. Even though not all these genes have been reported for the genome of *N. gaditana* [21], it is likely that orthologs exist, based on its fatty acid composition.

### Metabolism: Lipid and fatty acid degradation

Lipases are enzymes cleaving the carboxyl ester bonds of lipids. They can affect TAG metabolism through either TAG degradation or lipid remodeling, releasing fatty acids from membrane lipids for TAG biosynthesis. In Chlamydomonas, lipase-encoding genes were found to be highly regulated following N deprivation [35]. We probed the CCMP1779 genome for lipase-encoding genes with sequence similarity to those of *S. cerevisiae* and Chlamydomonas. A total of 52 putative lipase encoding genes were retrieved (Table S14). One predicted enzyme is similar to the three major TAG lipases in yeast (TGL3, TGL4, and TGL5) and the major TAG lipase (SDP1) in Arabidopsis [81], [82]. Deleting TGL3 and TGL4 from the yeast genome led to an increase in TAG content [83].

Fatty acids are degraded to acetyl-CoA by β-oxidation. In eukaryotes, β-oxidation can occur in the peroxisome or in the mitochondria. Although the intermediates of mitochondrial and peroxisomal pathways are identical, the reactions are performed by different proteins, and the functions of each pathway are different, when both pathways are present [84]–[86]. The main difference between the two types of β-oxidation is the first dehydrogenation of acyl-CoA. CCMP1779 has a gene encoding a predicted acyl-CoA oxidase similar to characterized plant and animal acyl-CoA oxidases [86], and several genes encoding enzymes similar to characterized human mitochondrial acyl-CoA dehydrogenases [87]. Furthermore, CCMP1779 also has genes encoding predicted peroxisomal and mitochondrial forms of the multifunctional enzyme, which performs both the hydration of the β-carbon and a second dehydrogenation to produce 3-ketoacyl-CoA. We also found genes encoding 4-ketoacyl thiolase enzymes, and several homologs to the enzymes required to degrade unsaturated fatty acids. In most cases, there were one or two genes predicted to encode Nannochloropsis enzymes that were more similar to either their plant peroxisomal homolog or human mitochondrial homolog, than to other Nannochloropsis enzymes that were predicted to perform a similar reaction. However, definitive assignment of some genes in CCMP1779 was limited because many of the β-oxidation enzymes are also very similar to those involved in mitochondrial amino acid degradation.

The specific contributions of the mitochondrial and peroxisomal β-oxidation to fatty acid degradation in heterokonts are unclear. Mammals and some non-yeast fungi have both pathways, whereas plants and yeast fungi (i.e. Saccharomyces) have only the peroxisome type. In addition other heterokont species *Ectocarpus siliculosus* [6] and *Thalassiosira pseudonana* [64] have genes for both types of β-oxidation. In most cells, the acetyl-CoA derived from β-oxidation is used to feed the TCA cycle to provide ATP, and/or it can be used to synthesize carbohydrates through the glyoxylate cycle and gluconeogenesis [84]. Indeed, the presence of genes encoding glyoxylate cycle enzymes, isocitrate lyase and malate synthase in CCMP1779, suggests that acetyl-CoA derived from peroxisomal β-oxidation of stored TAG is used to synthesize carbohydrates as is the major role of β-oxidation in plants [84]. However, this is in contrast to other organisms that have both types of β-oxidation (i.e. animals) where the mitochondrial form metabolizes the majority of the fatty acids and peroxisomes metabolize unusual fatty acids [84].

### Metabolism: Cell walls and polysaccharides

The synthesis pathways for polysaccharides and cell wall components are of special interest, because they markedly contribute to the harvested biomass and can be potentially converted to fuels. To gain insight into the types of polysaccharides synthesized by CCMP1779, neutral glycosyl residue composition analysis was done on alcohol-insoluble residues (AIR) prepared from cell cultures (Table 4). With respect to AIR preparations of terrestrial plants, which contain a diverse array of structural and storage polysaccharides that vary in sugar composition, CCMP1779 AIR is composed mainly of glucose, which accounted for approximately 90% of the neutral monosaccharides liberated by trifluoracetic acid (TFA). Of the remaining 6 neutral monosaccharides measured, ∼3.5% was mannose followed by trace amounts of rhamnose, fucose, arabinose, xylose, and galactose. These results show that Nannochloropsis lacks the polysaccharide diversity associated with land plants or other heterokonts [88]. Glucose can be a component of several types of polysaccharides, including storage polysaccharides, such as starch and laminarin (a β-1,3-glucan), and structural polysaccharides, such as hemicelluloses and cellulose. In two other heterokonts, oomycetes and brown algae, cellulose and laminarin are the main structural and storage polysaccharides, respectively [89]. To determine if laminarin was also present in CCMP1779 and to differentiate it from cellulose, AIR preparations were digested with either EGII (an enzyme specific for β-1,4-glucan) or laminarinase and the glycosyl residue composition of the enzyme-susceptible and –resistant fractions was determined. Results showed that EGII digestion liberated 85% of the glucose found in AIR, while 20% of glucose was liberated from AIR with laminarinase treatment. Therefore, AIR preparations contain mainly cellulose and laminarin and lack other complex polysaccharides associated with other members of the heterokonts.

**Table 4:** Monosaccharide composition analysis of CCMP1779 AIR.

	Monosaccharides (µg mg^−1^ AIR)						
Treatment	Rhamnose	Fucose	Arabinose	Xylose	Mannose	Galactose	Glucose
TFA^1^	1.67±0.31	1.50±0.16	0.25±0.06	0.31±0.54	3.15±0.44	1.53±0.18	76.38±7.08
Saeman's^2^	0.01±0.03	0.09±0.11	0.03±0.01	0.30±0.26	0.16±0.00	0.01±1.16	5.30±1.27

1 AIR preparations were treated with TFA, centrifuged, and the supernatant assayed for neutral sugar content by GC-MS analysis of alditol acetate derivatives.

2 The pellet from TFA-treated AIR were treated via the Saeman's hydrolysis and assayed for neutral sugar content as above.

A search of CCMP1779 genome identified 42 genes from 15 CAZy families that are predicted to encode glycosyltransferases (GT) and 44 genes from 14 CAZy families that are predicted to encode glycoside hydrolases (GH). Of the 86 GT and GH enzymes identified, only a small set is related to GTs and GHs involved in cell wall metabolism (Table S15). Two of the six CAZy family GT2 proteins identified in the survey were annotated as cellulose synthases (CESA) and are more similar to CESAs of cyanobacteria than plants. As expected from composition analysis results, none of the 4 remaining proteins showed similarity to plant cellulose synthase-like (CSL) proteins, some of which have been shown to be involved in plant hemicellulose biosynthesis. There were nine CCMP1779 proteins identified as belonging to CAZy family GH9 with high similarity to plant endoglucanases that are thought to function in cell wall remodeling during growth.

### Metabolism: Nitrogen assimilation and amino acid synthesis

Most eukaryotic photosynthetic organisms including algae are incapable of direct fixation of atmospheric N_2_. Instead they acquire N through a biological process known as N assimilation [90], [91]. Nitrate is one of the N-providing nutrients and the conversion of nitrate to nitrite by nitrate reductase is followed by a reduction to ammonia by nitrite reductase before N is incorporated into organic matter. Ammonia is assimilated into glutamine and glutamate by glutamate synthase [90], [91]. The CCMP1779 genome encodes a set of enzymes and transporters likely involved in the nitrogen assimilation common to other algae such as Ectocarpus [6]. However, only one gene each, encoding a putative nitrite, nitrate and ammonium transporter were identified in the CCMP1779 genome (Table S16). This is in contrast to the *N. gaditana* genome, for which two copies of each of the transporters were described [21], reflecting possible differences in the biology of the two algae.

Most bacteria, archaea, fungi, algae, and plants are capable of *de novo* amino acid biosynthesis. In plants, Asp-derived, aromatic, and branched-chain amino acids are predominantly or exclusively synthesized in the plastid [92]–[94]. The plastid in Nannochloropsis is surrounded by four membranes [95], which adds great complexity. For the biosynthetic pathways of Asp-derived, aromatic, and branched-chain amino acids, Arabidopsis has an average of 2.5 genes per enzyme activity but CCMP1779 an average of 1.3 (Figure S4). A more detailed analysis of the predicted pathways can be found in Figure S5, Tables S16 and S17, Text S1.

### Metabolism: Sulfate assimilation

Pathways for sulfate acquisition and biosynthesis of cysteine (Cys), methionine (Met) and glutathione (GSH) were suggested based on annotation of CCMP1779 to be fairly consistent with those known in vascular plant species [96], although they partly shared characteristics of yeast, heterokonts and bryophytes [97]–[99] (Table S18; Figure S6). A detailed description of predicted transport mechanisms and pathways is included in Text S1.

### Cellular processes: Organelle biogenesis

Plastids in plants and algae evolved from the same original endosymbiotic event [100]. In the heterokont lineage, a secondary endosymbiosis occurred, in which an algal descendant of the original endosymbiosis was engulfed by a second eukaryotic cell. Therefore plastids of Nannochloropsis contain four enveloping membranes; the inner two are equivalent to the envelope membranes of plants, and the outer two are similar to and continuous with the endoplasmic reticulum. Here, we focus on two proteinaceous systems, protein targeting and plastid division.

Plastid protein import is essential in all plastid containing organisms, as the majority of plastid genes are encoded in the nucleus, and these proteins must be imported into the plastid to function [100]. The proteinaceous machineries responsible are referred to as translocon at the outer envelope membrane of plastids (Toc), and translocon at the inner envelope membrane of plastids (Tic), with specific components referenced by molecular weight [101]. In CCMP1779, genes encoding four major components of the Tic complex were identified (Tic110, Tic20, Tic22, Tic62) (Table S19), while genes encoding Tic55, Tic40, and Toc complex members (75, 34, 159, 64) were not found. The gene encoding the stromal processing peptidase, which removes plastid targeting transit peptides [102] was found, but not a gene encoding a type I signal peptidase responsible for removing thylakoid lumen signal sequences [103]. Plastid specific members of the heat shock protein families 70 and 100 have been implicated in plastid protein import in higher plants [102], [104], and several members of each family were identified, though further characterization showed none of these was similar to plastid varieties from plants. Overall, plastid import genes identified are similar to those in other heterokonts such as Thalassiosira [105], which may indicate a conserved mechanism among heterokonts.

Gschloessl et al. developed a protein targeting prediction tool (HECTAR) based primarily on protein data from diatoms [106], specifically designed for the bipartite signal peptides present in heterokonts. To investigate the reliability of HECTAR predictions for Nannochloropsis proteins, we assembled a test set of manually curated proteins of known localization (Dataset S1) for plastid, mitochondrial, nuclear and secretory proteins. Of the plastid proteins a total of 44% were predicted correctly, in 23% of the sequences a signal sequence was detected but no plastid transit peptide and for 30% of proteins the tool failed to predict any type of signal peptide in the Nannochloropsis sequences (Table S20, Table S25). Even though this may indicate substantial differences between the architectures of the different targeting sequences, no false positives have been detected for either plastid or mitochondrial localization prediction making HECTAR a useful tool when positive results are retrieved.

### Cellular processes: Organelle division

Plastids are maintained by binary fission, which is driven by a macromolecular complex that forms at the division site and is derived partly from the cyanobacterial cell division machinery [107], [108]. The composition of the division complex differs in the red and green lineages, but two ubiquitous ring-forming contractile components (at least in organisms bearing primary plastids) are FtsZ and ARC5/DRP5B. Plastid FtsZ is a tubulin-like, stroma-localized protein of cyanobacterial cell division origin that probably constricts the inner envelope membrane. ARC5/DRP5B is a dynamin-related cytosolic protein of eukaryotic origin that constricts the outer envelope membrane.

Two genes encoding FtsZ proteins, both bearing predicted N-terminal ER signal peptides followed by downstream plastid transit peptides [109]–[111] were identified in CCMP1779, suggesting they localize to the plastid (Table S21). Similar bipartite targeting signals have been shown to direct FtsZs to the plastid in other organisms with secondary plastids [112]. Phylogenetic analysis [113] indicates these two proteins are most closely related to the plastidic FtsZs in red and heterokont algae (Figure S7), suggesting a conserved role for the CCMP1779 FtsZs in plastid division. A gene related to ARC5/DRP5B was also identified in CCMP1779 and grouped with other DRP5B protein sequences in phylogenetic analysis (Figure S8). However, it does not have a predicted signal peptide [111] suggesting that, if it plays a role in plastid division, it may function on the cytosolic side of the outermost membrane. A dynamin-related plastid division protein from the Apicomplexan parasite *Toxoplasma gondii* was recently shown to localize similarly [114].

Like cyanobacteria and most other prokaryotes, the α-proteobacterial ancestor of mitochondria also used FtsZ for cell division. Mitochondrial FtsZ has been lost from fungi, animals and plants. However, it has been retained in *Dictyostelium* and diverse algal species, including the red alga *Cyanidioschyzon merolae* and the heterokont alga *Mallomonas splendens*, where it likely functions in mitochondrial division [6], [115], [116]. A third FtsZ identified in CCMP1779 was predicted to bear a mitochondrial targeting sequence [109] and grouped with mitochondrial and α-proteobacterial FtsZs in phylogenetic analysis (Figure S7), suggesting it functions in mitochondrial division. Interestingly, single genes encoding proteins similar to MinC and MinD, which are components of a system that regulates FtsZ ring placement in bacteria [117], were also identified. Related proteins, presumably of cyanobacterial origin, are found in the green lineage, and MinD has been shown to function in plastid division [118], [119]. However, the CCMP1779 MinC- and MinD-like proteins were predicted to be targeted to mitochondria and clustered with proteins from non-cyanobacterial prokaryotes in phylogenetic analysis (Figure S9; Figure S10). The *E. siliculosus* genome appears to encode similar sequences (Figure S9 and S10). As mitochondrial MinC and MinD have not been described in other eukaryotes, these findings suggest a new variation on mitochondrial division that is conserved at least in some heterokonts. No other sequences with similarity to known green-lineage (ARC6, PARC6, PDV1, PDV2, MinE, ARC3, GC1, MCD1 [107] or red-lineage (PDR1 [120]) plastid division proteins, known mitochondrial division proteins (ZED [121], Fis1, MDA1 [122]), or to other bacterial cell division proteins (SulA, DivIVA, FtsW, ZipA, FtsA, Ftn2, Ftn6, SepF, ZapA [123]–[125]) could be identified in CCMP1779, consistent with the absence of these proteins in other heterokonts [126].

### Cellular processes: Light signaling and circadian regulation

Our analyses indicate that Nannochloropsis is likely to perceive blue light but it remains unknown whether this microalga can sense red or green light. We did not identify genes encoding canonical phytochrome or rhodopsin-like proteins. However, we found one gene encoding a protein with HisKA and HATPase_c domains but lacking other known protein domains (Table S22). We found several orthologs encoding likely blue light sensing proteins. We identified a cryptochrome gene (Table S22) encoding a protein that displays strong similarity to the recently characterized diatom CRYPTOCHROME PHOTOLYASE FAMILY PROTEIN 1 (CPF1) [127] (Figure S11, Table S22). CPF1 has both photolyase and transcriptional regulatory activities. We have also identified a gene for a likely CRY-DASH type protein (Figure S11; Table S22). Moreover, genes for three Aureochrome-like proteins were present [128] (Figure S12). Aureochromes of heterokonts are involved in photomorphogenesis under blue light [129]. These proteins contain a light-oxygen-voltage (LOV) domain with a FMN chromophore and a basic region/leucine zipper (bZIP) DNA binding domain, and are able to bind DNA in a blue light-dependent manner. It has been recently shown that Nannochloropsis biomass production is enhanced under blue light [130] and our findings indicate the importance of blue light signaling for this marine microalga.

Diel and circadian signals regulate numerous processes in unicellular algae such as the cell cycle, UV sensitivity and storage compound accumulation [131]–[133], but little is known about the circadian clock in non-green photosynthetic algae. We did not find any obvious candidates encoding proteins similar to plant, animal or bacterial clock proteins in CCMP1779. However, we identified two genes encoding bHLH-PAS proteins (Table S22). These proteins play a key role in the circadian regulation in animals, but are absent from plants [134], [135]. These two proteins appear to be conserved in diatoms but share no significant similarity to proteins in other organisms and only 25.4% identity to each other. We have also identified three genes encoding CCT (CONSTANS, CO-like, and TOC1) domain-containing proteins (Table S22). CCT- proteins are involved in the regulation of light, circadian and photoperiod responses in plants and green algae but are not found in animals [136], [137]. In plants and green algae, CCT domains come associated with either response regulator domains or DNA binding motifs with the CCT being at the C-terminus of the protein. Two of the CCT containing proteins (*No*CCT-1 and *No*CCT-2) predicted for CCMP1779 have the CCT domain at the C terminus, but in CCT-3 this domain found in the middle of the protein. The two proteins *No*CCT-1 and *No*CCT-2 display no similarity to any other proteins and also do not display any similarity to each other outside their CCT domains. In contrast, we find *No*CCT-3 like proteins in diatoms. In summary, this lack of conservation indicates that the Nannochloropsis circadian clock is likely to be different from clocks of plants or animals.

### Transcriptional regulation

Regulation of gene expression is a multi-step process, which occurs from DNA-RNA transcription to post-translational modification of a protein. However, for most genes, transcription is tightly controlled. In both prokaryotes and eukaryotes, a large number of regulatory proteins, including transcription factors (TFs) and other transcriptional co-regulators (TRs), influence the transcription process either positively or negatively. Transcription factors are able to modulate transcription by binding to the *cis*-elements in target genes promoters. Transcriptional co-regulators interact with TFs, assisting in controlling the transcription of specific genes via direct physical interactions with general transcription machinery or indirectly through modification of chromatin structure.

The availability of complete genome sequences facilitates genome-wide identification of transcription factors and transcriptional co-regulator. Computational studies, searching for genes containing conserved DNA binding domains, reported the occurrence of putative TFs and TRs in numerous species, including *Escherichia coli* [138], *Saccharomyces cerevisiae* [139], *Caenorhabditis elegans* [140], *Drosophila melanogaster* [141], *Arabidopsis thaliana* [142], [143], *Mus musculus* [144] and *Homo sapiens* [145]. In efforts to identify and classify all plant transcription regulatory proteins, several plant transcription factor databases have been established (PlnTFDB 3.0, http://plntfdb.bio.uni-potsdam.de/v3.0/; PlantTFDB 2.0, http://planttfdb.cbi.pku.edu.cn; AGRIS, http://arabidopsis.med.ohio-state.edu). These publicly available databases contain approximately 50 species covering the main lineages of the plant kingdom, including red algae, green algae, moss, ferns, gymnosperms, and angiosperms. Currently, more than 50,000 protein models have been collected, which can be catalogued into over 90 genes families.

The presence or absence of one or more characteristic domains (normally signature DNA-binding domains) determines the classification of genes in individual family. Based on the pipeline and basic rules for identification and classification of transcription factors and transcriptional co-regulators adopted by PlnTFDB 3.0 and PlantTFDB 2.0 [146], [147], a comprehensive analysis of CCMP1779 genome sequence was performed. In summary, 224 genes encoding 115 putative TFs and 109 putative TRs were identified, which represent ∼2.0% of the total number of estimated genes in CCMP1779 (Table 5 and Table S23). The CCMP1779 genomic content of TFs and TRs is close to that of *Ostreococcus tauri* and *Chlamydomonas reinhardtii* (2.1% and 1.5%, respectively) [146]. The identified 115 putative TFs belong to 20 transcription factor families and 109 TRs are members of 13 transcriptional co-regulator families. Only two plant-specific TF families (AP2-EREBP and LFY) and no plant-specific TRs were found in CCMP1779.

**Table 5:** Transcription factors in different eukaryotic species.

Family	*N. oceanica*	*C. reinhardtii*	*A. thaliana*	*H. sapiens*	*C. elegans*	*S. cerevisiae*
*ABI3VP1*		1	59			
AP2-EREBP*	6	11	160			
*ARR-B*		1	15			
bHLH	6	4	160	154	50	7
BSD	1	2	12	ND	ND	ND
bZIP	8	7	93	59	39	12
*C2C2-CO-like*		1	19			
*C2C2-Dof*		1	42			
C2C2-GATA		6	30	15	14	10
C2H2 (Zn)	1	5	104	644	150	39
C3H (Zn)	4	15	75	85	40	7
CCAAT	10	8	53	25	12	10
CPP (Zn)	4	1	9	4	2	
CSD		1	4	16	5	
E2F-DP	3	6	11	18	7	
FHA	12	12	17	44	12	14
G2-like		4	48			
HB		1	97	299	107	7
HSF	4	2	23	6	1	5
LFY*	1		1			
MADS		2	122	9	2	4
mTERF	4	1	36	ND	ND	ND
MYB	17	11	161	19	7	3
MYB-related	12	14	90	36	12	12
*PBF-2-like*		1	4			
*PLATZ*		3	13			
*RWP-RK*		14	14			
*SBP*		21	17			
Sigma70-like	5	1	6			
TAZ	2	2	9	4	6	
TIG	2	2		ND	ND	ND
Tub	3	3	12	6	2	
*WRKY*		1	84			
Zn-clus (Zn)	9			ND	ND	ND

Plant-specific TF families are in italics. (Zn) indicates the zinc-coordinating transcription factor families. ND, not determined.

* Plant-Specific TF families found in *N. oceanica*.

The largest family of transcription factors in CCMP1779 is the MYB superfamily. Each member of the MYB superfamily should possess a MYB DNA-binding domain, which is a helix-turn-helix structure of 50–53 amino acids with a central tryptophan cluster formed by three regularly spaced tryptophan residues within the MYB motif. Depending on the number of imperfect repeats of the MYB motif, the members of MYB family can be grouped into three classes: R2R3-MYB with two adjacent MYB repeats, R1R2R3-MYB (or MYB3R) with three adjacent MYB repeats, and MYB-related, a heterogeneous group in which the MYB motif is present either as a single copy or as a repeat [148]. Most of the MYB proteins in plants are of the R2R3-type and R1R2R3-MYB proteins are typical for animals. In Arabidopsis, the MYB superfamily is composed of 198 members, of whom 126 are *R2R3-MYB*, five are *R1R2R3-MYB*, 64 are *MYB-related* and three are atypical *MYB* genes [149]. We identified 29 genes which belong to MYB superfamily in CCMP1779, including 14 *R2R3-MYB* genes, three *R1R2R3-MYB* genes and twelve *MYB-related* genes. It has been suggested that R1R2R3-MYB proteins may have a conserved function in eukaryotes. The function of plant R1R2R3-MYB proteins might be more closely related to those of the MYB proteins in animals, such as controlling the cell cycle [150], [151]. Thus, the R1R2R3-MYB proteins in Nannochloropsis may play essential roles in the similar processes. In CCMP1779, R2R3-MYB proteins are relatively more abundant than R1R2R3-MYB and MYB-related proteins (14 of 29), as well as Arabidopsis (126 of 198) [149] and *C. reinhardtii* (16 of 18) [152]. The R2R3 format in plant MYB proteins has been suggested to be the result of loss of the R1 motif from an R1R2R3 ancestral gene (*pc-myb*-like gene) during evolution [150]. The plant *R2R3-MYB* genes mainly regulate plant-specific processes, such as secondary metabolism, development, determination of cell fate and identity, and responses to environmental stimuli [153].

It should be noted that there is no MADS box transcription factor present in the CCMP1779 genome. The MADS box TF family has been recognized as a large gene family across a variety of species including yeast, plants and humans. Its diverse functions range from controlling cell proliferation and differentiation in animals to regulating all major aspects of development in plants. CCMP1779 is not the first algal species reported without or with a limited number of MADS TFs. There is only one MADS-box TF identified in *Cyanidioschyzon merolae* and *Ostreococcus tauri*, and two found in *C. reinhardtii* [152]. This largely reduced number of MADS-box TFs in algal groups is most likely due to their unicellular identity. In contrast, zinc-coordinating transcription factors constitute a relatively major subset of TFs in CCMP1779 (18 of 115, ∼15.6%). Each zinc-coordinating TF possesses a zinc-finger domain, which has been demonstrated to be recruited in transcriptional regulation in prokaryotes [154]. Zinc-coordinating TFs constitute the largest family of transcription factors in animals and an expansion of zinc-finger domain containing TFs is observed during the evolution of eukaryotic organisms [142].

### Conclusions

The *N. oceanica* CCMP1779 draft genome and its extensive annotation reported here provides a starting point for further exploration of the biology and utility of this species. The primary focus here was on genes and pathways relevant for biofuel production. In addition, we were able to explore cellular and regulatory aspects through the participation of a large number of experts. However, the current manual annotation analysis must be considered work in progress and we would like to encourage the reader to visit the project website at www.bmb.msu.edu/Nannochloropsis.html for further exploration of the data. Comparison of the gene repertoires between *N. oceanica* and *N. gaditana* has indicated that the differences between these two species are comparable in magnitude to those observed between monocotyledonous and dicotyledonous plant species, which diverged from each other 150–200 million years ago. A substantial number of species-specific genes identified may reflect physiological and biochemical differences, that can be explored in future comparative studies. Experimental verification will likely provide insights into adaptations of the respective species to its specific ecological niche, and may also reveal the need for considering species-specific characteristics during genetic engineering for the purpose of biofuels feedstock production. For example possible differences in sets of genes relevant to fatty acid biosynthesis (acetyl-CoA carboxylase) may help us design strategies to maximize oil production in a given strain. Availability of genome sequences of different *Nannochloropsis* species in combination with targeted gene replacement by homologous recombination, which currently has only been documented for an *N. oceanica* strain closely related to CCMP1779 [8], will not only expedite the functional analysis of individual genes in Nannochloropsis, but is a prerequisite for future synthetic biology and engineering efforts focused on developing Nannochloropsis into a versatile feedstock for different industrial purposes.

## Materials and Methods

### Strains and growth conditions

The *Nannochloropsis sp.* strain used was CCMP1779, available from The Provasoli-Guillard National Center for Culture of Marine Phytoplankton (https://ncma.bigelow.org/). The cells were grown in liquid cultures under continuous light (∼80 µmole photons m^−2^ s^−1^). For N-replete growth, f/2 medium with 2.5 mM nitrate (f/2+N) was used [25]. For nitrogen-deprived experiments, N deprivation was applied by growth in f/2+N to 1×10^7^ cells mL^−1^, followed by transfer to f/2 without nitrogen source to 5×10^6^ cells mL^−1^ for an additional 30 hours.

### Nuclear transformation by electroporation

Initial transformation experiments were done with a construct described for nuclear transformation of *C. reinhardtii*, pHyg3 [27], containing a *C. reinhardtii* α-tubulin promoter and the coding sequence of the *Streptomyces hygroscopicus* aph7 gene conferring resistance to Hygromycin B. Subsequently, a plasmid custom made by DNA Cloning Service (http://www.dna-cloning.com) 497pLC-Hpt-SfiI, which contains a 35S promoter region, the aph7 coding sequence and a 35S terminator was digested with restriction endonucleases *Xba*I and *Xho*I to eliminate the promoter region. Additionally, the plasmid contains two *Sfi*I sites to allow directional cloning of further expression cassettes. The native LDSP promoter was amplified from CCMP1779 genomic DNA using the forward and reverse primers 5′-GGCCTAGGTACGTA-GGTCTCTAAGATGGAGTGGATGG-3′ and 5′-TTCAGCTG-TGTTGATGCGGGCTGAGATTGG-3′ and the resulting 790 bp PCR product cloned to the pGEMteasy vector system (Promega, http://www.promega.com) for sequencing resulting in pGEM-pLDSP. The promoter region was released from pGem-pLDSP by *Avr*II and *Pvu*II digest and blunt cloned to the dephosphorylated 497pLC-Hpt-SfiI backbone to result in the selection plasmid pSELECT100.

For transformation cells were harvested at a density of 1–2×10^7^ cells mL^−1^, washed with ice cold 375 mM sorbitol three times and resuspended in a final volume of 0.2 mL to a concentration of 5×10^8^ cells mL^−1^. In addition to 2–10 µg *Sna*BI linearized Plasmid DNA, a 10fold excess of salmon sperm DNA (Invitrogen, http://www.invitrogen.com) was supplied into the 2 mm electroporation cuvette. Electroporation was performed using a Bio-Rad (http://www.bio-rad.com) GenePulser II set to 600 Ώ resistance at a field strength of 11 kV cm^−1^leading to time constants of 20 to 25 ms. After the pulse the cells were resuspended in 5 mL of f/2 media and allowed to recover for 48 h in continuous light with shaking before they were spread on selection agar containing 50 µg ml^−1^ Hygromycin B using warm top agar (f/2 media, 0.05% Phytoblend (Caisson Laboratories, http://www.caissonlabs.com) in 1∶1 dilution (vol∶vol). Resistant colonies were observed as early as 10–14 days after electroporation; colonies were usually transferred after about 3 weeks.

### Southern analysis

For Southern analysis, 10 µg of DNA were digested with *Bam*HI and *Bam*HI/*Xba*I for pHyg3, or *Bam*HI only for the pSELECT100 and separated on an agarose gel (0.9% agarose, 75 Volts, 6 h runtime, 15 cm gel length) before blotting to a Hybond Nylon (Amersham, GE Health Care, http://www.gelifesciences.com) positively charged membrane overnight. Hybridization and detection was performed using the DIG labeling and detection system following the manufacturer's instructions (Roche Applied Sciences, http://www.roche-applied-sciences.com). Hybridization was done in 10 ml ULTRAhyb buffer (Invitrogen) at 68°C for pHyg3 or 42°C for pSelect100. The oligonucleotides for the probe synthesis by PCR were 5′-ACCAACATCTTCGTGGACCT-3′ and 5-‘CTCCTCGAACACCTCGAAGT-3′ for pHyg3 transformed cells and 5′-CGCGCTACTTCGAGCGGAGG-3′ and 5′-GCGCTTCTGCGGGCGATTTG-3′ for pSelect100 transformed cells using the respective plasmid as a template.

### DNA and RNA preparation for sequencing and analysis

For preparation of nuclear DNA a 50 mL cell culture (OD_750_ = 0.4 to 0.5) was harvested by centrifugation (4,500× g, 5 min). The cell pellet was lysed in 2× cetyltrimethylammonium bromide (CTAB) buffer (2% CTAB, 100 mM Tris-HCl pH 8.0, 1.4 M NaCl, and 20 mM EDTA) and incubated at 60°C for 60 min. The lysate was mixed with 1 volume of phenol/chloroform and centrifuged (13,000× g min). Transferred the supernatant to a new tube and repeated this step at least once until there was no white interphase. The DNA was precipitated by 1 volume isopropanol and 70% ethanol. High molecular weight DNA was examined by DNA gel electrophoresis.

To generate material for RNA-sequencing, cells were grown in 200 ml f/2+N to 1×10^7^ cells mL^−1^. The cultures were split in half and cells were collected by centrifugation (4,500× g, 5 min), with one pellet being resuspended in 200 mL f/2+N, and the other in 200 mL f/2-N. After 30 hours, the total RNA was isolated using TRIzol Reagent (Invitrogen) according to manufacturer's instructions. The RNA samples were cleaned up using RNeasy columns (Qiagen, http://www.qiagen.com) following the manufacturer's instruction.

### Assessment of RNA quantity and quality

The evaluation of RNA quantity and quality was done spectrophotometrically by UV absorbance profile. Additional analysis was performed using an RNA 6000 Nano LabChip Kit for microcapillary electrophoresis (Agilent 2100 Bioanalyzer, http://www.home.agilent.com). This eukaryotic total RNA nano-assay generated information about RNA integrity through electropherograms, gel picture, and RIN value (RNA Integrity Number) [155].

### Genome sequencing and hybrid genome assembly

For genome sequencing, two approaches were employed. First, Illumina GS-II was used to generate 55 bp paired-end reads with a 550 bp library and ∼2.3 Gb sequences were generated. The Illumina reads were filtered using FASTX (http://hannonlab.cshl.edu/fastx_toolkit/) with a minimum Phred quality score of 20. Next, Velvet [156] was used to assemble filtered Illumina reads, and a range of *k*-mer length were tested (31, 33, 35, 37, 39, 41, 43, 45, and 47). To determine an optimal k-mer length, 454 reads longer than 500 bp and *de novo* assembled transcripts were mapped to the genome assemblies using GMAP [157]. Based on how well the 454 reads and *de novo* transcripts mapped on to the Illumina assemblies, as well as N50s, numbers of contigs, assembly sizes, and numbers of total reads assembled, *k*-mer length of  = 35 was chosen for generating the final Illumina assembly. Newbler (454 Life Sciences) was used to assemble 454 reads (single-end reads, 449.9 MB sequences) with the “Large Genome Option”.

Illumina and 454 assemblies were combined by iterative Minimus2 [158]. Minimus2 was first run with a minimum identity of 98% among and between Illumina and 454 contigs based on and all-against-all contig similarity searches with BLAST [159]. If one contig had an alignment ≥200 bp and an identity ≥98% with ≥2 other contigs, only the longest contig among the matching contigs was kept and the rest were set aside before re-running Minimus2. This step was performed because such contigs may represent mis-assembled sequences and will confound Minimus2 as to which contigs it should assemble. A similar procedure was used in the assembly of the *Albugo laibachii* genome [160]. In the next iteration, the contigs set aside beforehand were added back to the assembly and Minimus2 was run again. After another three iterations of Minimus2 run, an optimized assembly was generated.

To assess assembly quality, long 454 reads with high Phred scores were mapped to the genome assembly. First, 454 reads were trimmed from the 3′ end with a minimum Phred score of 20. Then, sequences longer than 200 bp were aligned to the genome using BLAST to determine if a 454 read was broken up in >1 contigs. We also used *de novo* transcript assemblies (see next section) to assess genome assembly quality. The genomic sequence data are deposited in NCBI SRA (SRP013753).

### Transcript assembly and differential expression analysis

*De novo* transcript assemblies were generated from 55 bp directional single-end Illumina reads of N-replete and N-depleted conditions (NCBI/GEO GSE36959) using Oases (http://www.ebi.ac.uk/~zerbino/oases/). First, Oases was run for *k*-mer lengths of 23, 25, 27, 29, 31, 33, 35, and 37, and the results were compiled. To identify a set of high confidence transcripts from the *de novo* assemblies, proteins from six sequenced heterokont genomes, including *Ectocarpus siliculosus* [6], *Pythium ultimum* [161], *Phytophthora sojae* [162], *Phytophthora ramorum* [162], *Thalassiosira Pseudonana* [64], and *Phaeodactylum tricornutum* [4], were aligned to the *de novo* transcripts and only those with significant matches to known proteins were kept. These transcripts with cross-genome matches were mapped back to the Illumina genome assemblies to evaluate genome assembly quality. In addition to *de novo* transcript assembly, we generated a genome-based transcript assembly.

Transcriptomic reads from N-replete and N-depleted conditions were separately mapped to the hybrid genome assembly using Tophat [160] (parameters: -I 10 –I 3000 –library-type fr-unstranded –g 1). The mapped reads were assembled into transcripts using Cufflinks [163] (-I 3000 –library-type fr-secondstrand) and a set of transcripts was generated for each condition.

### Genome annotation

The MAKER genome annotation pipeline [28] was used to annotate the genome. The first run of MAKER was performed using the est2genome option in the absence of a trained gene predictor. Transcripts from both N-replete and N-deprived growth conditions were provided to MAKER along with protein sequences from the above mentioned six sequenced heterokonts. Gene models obtained from the first run were used to train *ab initi*o gene prediction programs SNAP [164] and Augustus [165]. With the trained models, MAKER was rerun. The gene models from the rerun were used for training SNAP and Augustus again. The second round training models were provided to run MAKER for the third time to generate the final annotations. The protein sequences were searched for Pfam domain Hidden Markov Models using HMMER3 [166] with trusted cutoffs. CEGMA was run on the genome assembly using default settings [30]. A total of 11,973 genes (12,012 protein models considering alternative splice forms) were recovered with an average AED score of 0.555. During the course of the study, a new version of MAKER was released. Thus we conducted a second annotation run with the most recent MAKER version, a more recent repeat library, and a larger protein evidence dataset. Given that the AED distributions were highly similar between these two annotation datasets (Figure S12A, S12B) only annotation results from the first set of analysis were used throughout.

InterProScan [167] was used to identify Pfam protein domains within the predicted protein sets from *Nannochloropsis oceanica* CCMP1779 and six other heterokonts. Protein families were identified by grouping proteins with identical protein domains, and the number of proteins from each species that were classified into each protein family was tallied. Figure S2 shows the percentages of proteins that have at least one InterPro domain, and those that have none, of each species.

### Functional annotation and determination of differential expression

Blast2GO [32] (http://blast2go.com/b2ghome) was used for functional annotation of predicted protein models with the default settings for the mapping and annotation step. The initial BLAST [159] search was performed with an e-value cut-off of 10^−5^ and a maximum of 20 blast hits. This results in Gene Ontology (GO) annotations of 5,980 *N. oceanica* genes (in 4,012 GOs) and 3,008 *N. gaditana* genes (in 3,205 GOs). Fisher's exact test was used to assess if either the number of conserved or species-specific genes are over-represented in any GO category.

Cuffdiff from Cufflinks package [163] was used to analyze the differential gene expression under N-replete and N-deprived growth conditions. Fisher's exact tests were performed to determine the enrichment of each GO category in up- and down-regulated gene clusters and at the 1% significance level based on p-values.

### Comparison of *Nannochloropsis* genomes

OrthoMCL [168] was used to identify Orthologous Groups (OGs) of genes in *N. gaditana*, *N. oceanica* CCMP1779, and *E. siliculosus* (run parameters: percentMatchCutoff = 50, evalueExponentCutoff = −5). BLAST [169] was used to identify significant matches of lineage-specific genes across species. A significant match was defined as identity ≥47.04% (5 percentile in the identity distribution of one-to-one orthologs between *N. gaditana* and *N. oceanica*), Expect value≤10^−5^, alignment length ≥30 amino acids, and ≥50% of the protein sequence covered in the alignment. The orthologous group assignments as well as lists of species-specific genes are detailed in Table S5.

### Database tools

To allow easy access to the CCMP1779 genome data, we released a public version of the genome browser along with a basic BLAST tool to search nucleotide and protein databases, accessible at www.bmb.msu.edu/nannochloropsis.html. The genome browser contains EST data aligned to the latest genome assembly as well as alternative gene models in addition to the final models retrieved from the MAKER gene annotation pipeline described above.

### Collection and identification of repetitive sequences

Repetitive sequences were first collected with RECON (version 1.06, [170], http://www.repeatmasker.org/), with a cutoff of 5 copies. This resulted in a total of 175 repetitive sequences. Two sequences matching non-transposase proteins were considered to represent gene families and were excluded. Thereafter, repetitive sequences with more than 10 copies were manually curated to verify their identity, individuality and 5′/3′ boundaries. This was achieved by pair wise comparison of sequence contigs containing the relevant repeats using the “gap” program available from the GCG package (version 11.0, Accelrys Inc., San Diego, CA). A boundary was defined as the position to which sequence homology is conserved between the aligned sequences, and sequences flanking the boundary of the putative element were compared with that of a known transposable element (TE). Furthermore, the sequences immediately flanking the element boundaries were examined for the possible presence of target site duplication, which is created during transposition. Each transposon family has unique terminal sequences and target site duplication, which can aid in the identification of a specific transposon [171]. For some large transposable elements, fragmented sequences identified by RECON were joined to derive a compete sequence.

To recover transposable elements that are less than 5 copies, the assembled sequence was masked using the repeat library generated by RECON. Thereafter, the masked sequence was used to search against known transposons at the protein level (BLASTX E<10^−5^, RepBase14.12) (http://www.girinst.org/repbase/). To further eliminate fortuitous matches between the genomic sequence and known transposase, a custom script was used to exclude matches where two amino acids contribute to more than 50% of the identity. Sequences matching known transposons were considered to be transposon sequences and were included in the repeat library, together with the sequences collected using RECON. The repeat library was then used to mask the entire genome using RepeatMasker (RepeatMasker-open-3-2-7, http://www.repeatmasker.org/) with default settings. The copy number and genome fraction of each group of transposon was obtained from the summary table provided in the RepeatMasker output.

### Phylogenetic analysis

Unless stated otherwise, protein sequences, derived from the gene models, were used as BLAST queries to search the NCBI database. Sequences from the *N. oceanica* CCMP1779 gene models and related protein sequences from various organisms obtained from NCBI were used to generate a multiple sequence alignment using the Molecular Evolutionary Genetics Analysis 5 (MEGA5) program [113] and the Multiple Sequence Comparison by Log- Expectation (MUSCLE) algorithm [172]. The alignment file was used to create a Maximum Likelihood phylogenetic tree with 1000 rounds of bootstrapping.

### Lipid analysis

Lipids were extracted from lyophilized materials following the Folch method [173] with modifications as previously described [174]. Lipids were analyzed by a combination of thin layer and gas liquid chromatography as described in [174].

### Electron microscopy

For electronic microscopy analysis, 400 mL of log phase N-replete and 48 h N-deprived cultures were centrifuged (4,500× g, 5 min) and resuspended in 260 mM NaCl in order to maintain the same osmolarity as in the F/2 medium (510 to 530 mOsm/Kg). Cells were then fixed in 2% glutaraldehyde buffered by 75 mM NaCl and 100 mM PIPES (pH 8.5) for 3 h at 4°C. After fixation cells were washed with 100 mM PIPES for five times and kept at 4°C before infiltration and embedding. Sections were analyzed by transmission electron microscopy using a JEOL100 CXII instrument (Japan Electron Optics Laboratories, http://www.jeol.com/).

### Hydrogen production assay

H_2_ production was measured as described previously [175]. Briefly, a culture of cells was concentrated to 75 µg chlorophyll/mL in 2 mL of anaerobic F/2 nutrient media, sealed in 13-mL vials, and shaken at 100 rpm at 22°C in the dark to induce anaerobiosis. In parallel, aerobic cells were incubated under similar conditions, but exposed to the atmosphere. H_2_ evolution was measured by incubating 0.1 mL of either aerobically- or anaerobically-induced cells with 1.9 mL of H_2_ evolution assay solution (F/2 nutrient media, 100 mM sodium dithionite, 10 mM methyl viologen) in a 13.0 mL serum vial at 22°C in the dark with continuous shaking. At fixed time points, 20 µL of headspace gas was injected into a TRACE GC Ultra Gas Chromatograph (Thermo Scientific, http://www.thermoscientific.com) using a 100 µL syringe. H_2_ accumulation was measured by comparison of the peak area against a standard curve.

### Cell wall analysis

For cell wall analysis, cell cultures of CCMP1779 were pelleted, washed three times with distilled water, and lyophilized. Alcohol insoluble residues (AIR) were prepared from lyophilized CCMP1779 samples and subjected to neutral glycosyl residue composition analysis according to procedures described in Cavalier et al. [176]. To differentiate between cellulose and laminarin, AIR samples were digested with either 10 U of EGII or 1 U of laminarinase in 50 mM sodium acetate buffer (pH 5.0) at 37°C for 48 hours. Reactions terminated with the addition of ethanol to a final concentration of 70%, heated at 95°C for 10 minutes, and microfuged for 15 min at 14 k× g to pellet enzyme-resistant polysaccharides. The respective supernatants and pellets were separated and monosaccharide composition analysis performed on each fraction as described above.

## Supporting Information

Dataset S1 HECTAR test set protein sequences.
(TXT)

Figure S1 Nuclear Transformation. Southern Hybridization of CCMP 1779 transgenic clones transformed pSelect100 plasmid. C, DNA digested with BamHI restriction endonuclease, U, DNA probed undigested. Lower panel depicts a schematic map of the SnaBI linearized plasmids with the basic features indicated. P LDSP, Promoter region of LDSP (NannoCCMP1779_4188), ORF aphVII, open reading frame of, T 35S, terminator sequence of 35S.
(EPS)

Figure S2 Gene Annotation. (A) Annotation Edit Distance (AED) distribution of gene models in the first annotation set after eliminating entries with AED = 1. (B). AED distribution of gene models in the second annotation after eliminating entries with AED = 1. (C) Proportion of gene models with protein domain hits in different heterokonts (abbreviated as indicated in Materials and Methods).
(EPS)

Figure S3 AED distributions of conserved and *N. oceanica*-specific genes. (A) AED distribution of *N. oceanica* genes in a conserved OG; (B) AED distribution of *N. oceanica*-specific genes.
(EPS)

Figure S4 Comparison of biosynthetic pathways of Asp-derived, aromatic and branched-chain amino acids between Arabidopsis and *N. oceanica*. (A) biosynthesis of Asp-derived amino acids Lys, Met and Thr; (B) biosynthesis of aromatic amino acids Phe, Tyr and Trp; (C) biosynthesis of branched-chain amino acids Ile, Leu and Val. The first number (red) in parentheses are numbers of genes per activity in Arabidopsis; the second number (blue) in parentheses are predicted numbers of genes per activity in *N. oceanica*. A value of 0.5 indicates incomplete or partial gene sequence. Because there are a large number of aminotransferases in *N. oceanica* (and Arabidopsis) and because substrate specificity of these aminotransferases hasn't been experimentally determined, the number of PAT genes in N. oceanica is not proposed in this figure (indicated by the asterisk). ADH, arogenate dehydrogenase; ADT, arogenate dehydratase; AHAS, acetohydroxyacid synthase; AK, Asp kinase; ASA, anthranilate synthase alpha subunit; ASB, anthranilate synthase beta subunit; ASD, Asp semialdehyde dehydrogenase; BCAT, branched-chain aminotransferase; CBL, cystathionine beta lyase; CGS, cystathionine gamma synthase; CM, chorismate mutase; CS, chorismate synthase; DAHPS, 3-deoxy-D-arabino-heptulosonate-7-phosphate synthase; DAPAT, diaminopimelate aminotransferase; DAPDC, diaminopimelate decarboxylase; DAPE, diaminopimelate epimerase; DAQDH, dehydroquinate dehydratase; DAQS, dehydroquinate synthase; DHDPR, dihydrodipicolinate reductase; DHAD, dihydroxyacid dehydratase; DHDPS, dihydrodipicolinate synthase; EPSPS, 5-enolpyruvylshikimate-3-phosphate synthase; HMT, homocysteine S-methyltransferase; HSDH, homoserine dehydrogenase; HSK, homoserine kinase; IGPS, indole-3-glycerol phosphate synthase; IMD, isopropylmalate dehydrogenase; IPMS, isopropylmalate synthase; IPMIL, isopropylmalate isomerase large subunit; IPMIS, isopropylmalate isomerase small subunit; KARI, ketolacid reductoisomerase; MetH, cobalamin-dependent Met synthase; MS, cobalamin-independent Met synthase; PAI, phosphoribosylanthranilate isomerase; PAT, prephenate aminotransferase; PRT, anthranilate phosphoribosyltransferase; SDH, shikimate dehydrogenase; SK, shikimate kinase; TD, Thr deaminase; TS, Thr synthase; TSA, Trp synthase alpha subunit; TSB, Trp synthase beta subunit.
(EPS)

Figure S5 Fused genes in essential amino acid biosynthesis in *N. oceanica* genome. (A), Asp kinase and homoserine kinase genes; (B), dehydroquinate dehydratase and shikimate dehydrogenase genes; (C), arogenate dehydratase and arogenate dehydrogenase genes; (D), anthranilate synthase alpha and beta subunit genes; (E), indole-3-glycerol phosphate synthase and phosphoribosylanthranilate isomerase genes; (F), Trp synthase alpha and beta subunit genes. Red and blue arrows represent genes in the biosynthetic pathways of Asp-derived and aromatic amino acids, respectively.
(EPS)

Figure S6 Sulfur metabolism. (A) Domain structure of putative PAPS synthetase. (B) Pathways for sulfate reduction and PAPS biosynthesis. (C) Cys, Met and GSH biosynthesis and metabolism. Abbreviations of metabolites: APS, adenosine 5′-phosphosulfate; γ-GluCys, γ-glutamylcysteine; GSH, glutathione; PAPS, 3′-phosphoadenosine 5′-phosphosulfate; SAH, S-adenosylhomocysteine; SAM, S-adenosylmethionine. Abbreviations of enzymes: APK, adenosine 5′-phosphosulfate kinase; APR-B, type-B adenosine 5′-phosphosulfate reductase; ATPS, ATP sulfurylase; CBL, cystathionine β-lyase; CBS, cystathionine β-synthase; CGL, cystathionine γ-lyase; CGS, cystathionine γ-synthase; CS, cysteine synthase; γ-ECS, γ-glutamylcysteine synthetase; GSHS, glutathione synthetase; HCS, homocysteine synthase; HSAT, homoserine acetyltransferase; MS, methionine synthase; SAHH, S-adenosylhomocysteine hydrolase; SAMS, S-adenosylmethionine synthetase; SAT, serine acetyltransferase; SiR, sulfite reductase.
(EPS)

Figure S7 Phylogenetic analysis of FtsZ proteins. FtsZ sequences from plants, algae, and bacteria were aligned in MEGA5 [113] using the Multiple Sequence Comparison by Log- Expectation (MUSCLE) algorithm [172]. The multiple sequence alignment was then used to generate a Maximum Likelihood phylogenetic tree with 1,000 rounds of bootstrapping. Bootstrap values are shown at branch points. Protein accession numbers for each sequence are shown in parentheses. * This gene model is from augustus or snap gene annotation and was found superior to the final maker annotation after manual examination.
(EPS)

Figure S8 Phylogenetic analysis of DRP proteins. Eukaryotic DRP sequences were aligned in MEGA5 [113] using the Multiple Sequence Comparison by Log- Expectation (MUSCLE) algorithm [172]. The multiple sequence alignment was then used to generate a Maximum Likelihood phylogenetic tree with 1,000 rounds of bootstrapping. Bootstrap values are shown at branch points. Protein accession numbers for each sequence are shown in parentheses.
(EPS)

Figure S9 Phylogenetic analysis of MinC proteins. MinC sequences from algae and bacteria were aligned in MEGA5 [113] using the Multiple Sequence Comparison by Log- Expectation (MUSCLE) algorithm [172]. The multiple sequence alignment was then used to generate a Maximum Likelihood phylogenetic tree with 1,000 rounds of bootstrapping. Bootstrap values are shown at branch points. Protein accession numbers for each sequence are shown in parentheses.
(EPS)

Figure S10 Phylogenetic analysis of MinD proteins. MinD sequences from plants, algae, and bacteria were aligned in MEGA5 [113] using the Multiple Sequence Comparison by Log- Expectation (MUSCLE) algorithm [172]. The multiple sequence alignment was then used to generate a Maximum Likelihood phylogenetic tree with 1,000 rounds of bootstrapping. Bootstrap values are shown at branch points. Protein accession numbers for each sequence are shown in parentheses.
(EPS)

Figure S11 Phylogenetic analysis of Nannochloropsis cryptochrome/photolyase proteins. Phylogenetic analysis using the neighbor-joining method of a ClustalW alignment of 44 proteins was performed in MEGA 5. The percentage of replicate trees in which the associated taxa clustered together in the bootstrap test (1000 replicates) is shown next to the branches. The evolutionary distances were computed using the JTT matrix-based method. Scale bar, 0.5 substitutions per site. The ID numbers for the diatom proteins are according to the annotated genomes at http://genome.jgi-psf.org/. * This gene model is from augustus or snap gene annotation and was found superior to the final maker annotation after manual examination.
(EPS)

Figure S12 Phylogenetic analysis of Nannochloropsis AUREO proteins. Phylogenetic analysis using the neighbor-joining method of a ClustalW alignment of 18 proteins was performed in MEGA 5. The percentage of replicate trees in which the associated taxa clustered together in the bootstrap test (1000 replicates) is shown next to the branches. The evolutionary distances were computed using the JTT matrix-based method. Scale bar, 0.2 substitutions per site.
(EPS)

Table S1 Comparison of the effect of selected antibiotics on different Nannochloropsis species. Shown are the lethal doses in µg/mL determined by plating dilutions of cell suspensions on half salinity f/2 agar plates. ‘>’ indicates the highest concentration of the respective antibiotic tested and no detectable impact on cell growth observed. All of the Nannochloropsis strains listed here were found to be resistant to the following antibiotics with the respective concentrations in µg/mL given in parenthesis: Rifampicin (10), Benomyl (5), Nystatin (5), Spectinomycin (100), Ampicillin (200), Chloramphenicol (100).
(DOCX)

Table S2 Growth parameters of *N. oceanica* CCMP1779 in f/2 medium using different supplements. V = f/2 Vitamine mix, Gl = Glucose, Fr = Fructose, curves have been determined in triplicates based on cell density and fitted to a sigmoidal logistic function type 1 individually using OriginPro software (y = a/1+exp(−k*(x*x_c_))). Parameters a (Amplitude, here: max. cell density in cell/ml), x_c_ (time of ½a in d) and k (coefficient, intrinsic growth rate d^−1^) are arithmetic means with standard deviation.
(DOCX)

Table S3 Number of resistant colonies achieved by electroporation of *N. oceanica* CCMP1779 cells in the presence of linearized pHyg3, pSelect100 plasmids per µg linearized plasmid DNA and transformation rates. Arithmetic means are given from three (pSelect100) or four (pHyg3 and no plasmid control) independent experiments with standard deviation. All transformation reactions contained denatured salmon sperm DNA in 10-fold excess compared to plasmid DNA.
(DOCX)

Table S4 Enriched GO categories in up- and down-regulated genes during N-deprived versus N-replete conditions based on RNAseq data.
(DOCX)

Table S5 Comparison of *N. gaditana* and *N. oceanica* CCMP1779 protein sets.
(XLS)

Table S6 Enriched GO categories in conserved OGs and *N. oceanica* CCMP1779-specific and *N. gaditana*-specific genes.
(DOCX)

Table S7 Putative genes identified to be involved in photosynthetic electron transport in CCMP1779. In cases where no gene model was structurally annotated, genome coordinates are given.
(DOCX)

Table S8 Genes predicted to encode for Violaxanthin-Chlorophyll binding proteins (VCP) in CCMP1779 genome and there designation in the phylogenetic tree (Fig. 6).
(DOCX)

Table S9 Putative genes identified to be involved in xanthophyll synthesis.
(DOCX)

Table S10 Genes putatively involved in central carbon metabolism and possible carbon concentrating mechanism.
(DOCX)

Table S11 Functional annotation of putative genes involved in H_2_ metabolism and oxidative phosphorylation identified in the CCMP1779 genome.
(DOCX)

Table S12 Fatty acid composition of the major glycerolipids of Nannochloropsis CCMP1779. Averages are presented (n = 3) with standard deviation in parenthesis.
(DOCX)

Table S13 Functional annotation of putative genes involved in fatty acid and glycerolipid biosynthesis.
(DOCX)

Table S14 Genes predicted to encode enzymes putatively involved in fatty acid mobilization and degradation.
(DOCX)

Table S15 Genes predicted to encode enzymes putatively involved in cell wall metabolism.
(DOCX)

Table S16 Predicted genes in the biosynthetic pathways of Asp-derived, aromatic and branched-chain amino acids and in nitrogen assimilation in CCMP1779.
(DOCX)

Table S17 Presence of fused genes in essential amino acid biosynthesis in representative bacteria, cyanobacteria, green algae, diatoms, Nannochloropsis and higher plants.
(DOCX)

Table S18 Putative Nannochloropsis genes involved in sulfate assimilation and metabolism.
(DOCX)

Table S19 Putative chloroplast protein import related genes identified in the CCMP1779 genomic sequence.
(DOCX)

Table S20 Summary of testing the HECTAR heterokont protein localization prediction tool. Detailed information on the tested sequences and results is available in Table S25.
(DOCX)

Table S21 Predicted genes involved in organelle division.
(DOCX)

Table S22 Genes predicted to be involved in light signaling.
(DOCX)

Table S23 Putative transcription factors and transcriptional regulators.
(DOCX)

Table S24 Protein domain search results for 6 different heterokonts.
(XLSX)

Table S25 Predicted subcellular localization of proteins.
(XLSX)

Text S1 Supplemental results and discussion. Additional annotation is provided for genes predicted to be involved in ROS scavenging systems, oxidative phosphorylation, amino acid biosynthesis, degradation of branched chain amino acids, sulfate uptake and metabolism, and histones and histone variants.
(DOC)
