# Correction: Genome, Functional Gene Annotation, and Nuclear Transformation of the Heterokont Oleaginous Alga *Nannochloropsis oceanica* CCMP1779

**DOI:** 10.1371/journal.pgen.1006802

**Published:** 2017-05-23

**Authors:** Astrid Vieler, Guangxi Wu, Chia-Hong Tsai, Blair Bullard, Adam J. Cornish, Christopher Harvey, Ida-Barbara Reca, Chelsea Thornburg, Rujira Achawanantakun, Christopher J. Buehl, Michael S. Campbell, David Cavalier, Kevin L. Childs, Teresa J. Clark, Rahul Deshpande, Erika Erickson, Ann Armenia Ferguson, Witawas Handee, Que Kong, Xiaobo Li, Bensheng Liu, Steven Lundback, Cheng Peng, Rebecca L. Roston, Sanjaya, Jeffrey P. Simpson, Allan TerBush, Jaruswan Warakanont, Simone Zäuner, Eva M. Farre, Eric L. Hegg, Ning Jiang, Min-Hao Kuo, Yan Lu, Krishna K. Niyogi, John Ohlrogge, Katherine W. Osteryoung, Yair Shachar-Hill, Barbara B. Sears, Yanni Sun, Hideki Takahashi, Mark Yandell, Shin-Han Shiu, Christoph Benning

S2 Fig. is a duplicate of Fig. 2. The authors have provided the correct version of S2 Fig. which can be viewed below.

## Supporting information

S2 Fig Gene annotation. (A) Annotation Edit Distance (AED) distribution of gene models in the first annotation set after eliminating entries with AED = 1. (B). AED distribution of gene models in the second annotation after eliminating entries with AED = 1. (C) Proportion of gene models with protein domain hits in different heterokonts (abbreviated as indicated in Materials and Methods).
(JPG)
