# Supplementary material for: Genome, Functional Gene Annotation, and Nuclear Transformation of the Heterokont Oleaginous Alga Nannochloropsis oceanica CCMP1779
Source: PLoS Genet. 2012 Nov 15;8(11):e1003064. doi: 10.1371/journal.pgen.1003064 (PMC3499364; doi:10.1371/journal.pgen.1003064)
Supplement: Table S1 — Comparison of the effect of selected antibiotics on different Nannochloropsis species. Shown are the lethal doses in µg/mL determined by plating dilutions of cell suspensions on half salinity f/2 agar plates. ‘>’ indicates the highest concentration of the respective antibiotic tested and no detectable impact on cell growth observed. All of the Nannochloropsis strains listed here were found to be resistant to the following antibiotics with the respective concentrations in µg/mL given in parenthesis: Rifampicin (10), Benomyl (5), Nystatin (5), Spectinomycin (100), Ampicillin (200), Chloramphenicol (100). (DOCX) [file pgen.1003064.s014.docx]

**Table S1.** Comparison of the effect of selected antibiotics on different Nannochloropsis species. Shown are the lethal doses in µg/mL determined by plating dilutions of cell suspensions on half salinity f/2 agar plates. ‘>’ indicates the highest concentration of the respective antibiotic tested and no detectable impact on cell growth observed. All of the Nannochloropsis strains listed here were found to be resistant to the following antibiotics with the respective concentrations in µg/mL given in parenthesis: Rifampicin (10), Benomyl (5), Nystatin (5), Spectinomycin (100), Ampicillin (200), Chloramphenicol (100).

| Species | Strain | Zeocin | Paromomycin | Hygromycin B | Spectinomycin |
| --- | --- | --- | --- | --- | --- |
| *N. oceanica* | ccmp1779 | 5 | 5 | 25 | >100 |
|  | ccmp531 | 5 | 10 | 50 | >100 |
| *N. granulata* | ccmp529 | 5 | >200 | 100 | >100 |
| *N. salina* | ccmp369 | 5 | >200 | >100 | >100 |
| *N. gaditana* | ccmp1775 | 5 | >100 | 100 | >100 |
| *N. gaditana* | ccmp536 | 5 | >100 | >100 | >100 |
