# Supplementary material for: Genome, Functional Gene Annotation, and Nuclear Transformation of the Heterokont Oleaginous Alga Nannochloropsis oceanica CCMP1779
Source: PLoS Genet. 2012 Nov 15;8(11):e1003064. doi: 10.1371/journal.pgen.1003064 (PMC3499364; doi:10.1371/journal.pgen.1003064)
Supplement: Table S2 — Growth parameters of N. oceanica CCMP1779 in f/2 medium using different supplements. V = f/2 Vitamine mix, Gl = Glucose, Fr = Fructose, curves have been determined in triplicates based on cell density and fitted to a sigmoidal logistic function type 1 individually using OriginPro software (y = a/1+exp(−k*(x*xc))). Parameters a (Amplitude, here: max. cell density in cell/ml), xc (time of ½a in d) and k (coefficient, intrinsic growth rate d−1) are arithmetic means with standard deviation. (DOCX) [file pgen.1003064.s015.docx]

**Table S2.** Growth parameters of *N. oceanica* CCMP1779 in f/2 medium using different supplements. V= f/2 Vitamine mix, Gl = Glucose, Fr=Fructose, curves have been determined in triplicates based on cell density and fitted to a sigmoidal logistic function type 1 individually using OriginPro software (y=a/1+exp(-k*(x*x_c_))). Parameters a (Amplitude, here: max. cell density in cell/ml), x_c_ (time of ½a in d) and k (coefficient, intrinsic growth rate d^-1^) are arithmetic means with standard deviation.

|  | **f/2** |  | **f/2+V** |  | **f/2+Gl** |  | **f/2+Fr** |  |
| --- | --- | --- | --- | --- | --- | --- | --- | --- |
| a | 6.3E7 | +/-8.5E6 | 6.4E7 | +/-8.3E6 | 8.7E+07 | +/-1.7E7 | 1.5E8 | +/-7.4E6 |
| x_c_ | 13.65 | +/-1.50 | 11.47 | +/-0.79 | 11.01 | +/-0.90 | 12.86 | +/-0.12 |
| k | 0.66 | +/-0.17 | 0.63 | +/-0.15 | 0.61 | +/-0.07 | 0.45 | +/-0.01 |
