# Supplementary material for: Genome, Functional Gene Annotation, and Nuclear Transformation of the Heterokont Oleaginous Alga Nannochloropsis oceanica CCMP1779
Source: PLoS Genet. 2012 Nov 15;8(11):e1003064. doi: 10.1371/journal.pgen.1003064 (PMC3499364; doi:10.1371/journal.pgen.1003064)
Supplement: Table S3 — Number of resistant colonies achieved by electroporation of N. oceanica CCMP1779 cells in the presence of linearized pHyg3, pSelect100 plasmids per µg linearized plasmid DNA and transformation rates. Arithmetic means are given from three (pSelect100) or four (pHyg3 and no plasmid control) independent experiments with standard deviation. All transformation reactions contained denatured salmon sperm DNA in 10-fold excess compared to plasmid DNA. (DOCX) [file pgen.1003064.s016.docx]

**Table S3**: Number of resistant colonies achieved by electroporation of *N. oceanica* CCMP1779 cells in the presence of linearized pHyg3, pSelect100 plasmids per µg linearized plasmid DNA and transformation rates. Arithmetic means are given from three (pSelect100) or four (pHyg3 and no plasmid control) independent experiments with standard deviation. All transformation reactions contained denatured salmon sperm DNA in 10-fold excess compared to plasmid DNA.

|  | # resistant colonies per µg plasmid DNA | | transformation rate (integrations per cell) | |
| --- | --- | --- | --- | --- |
| pHyg3 | 7.68 | +/-6.38 | 1/13,029,316 | +/-6.38E-08 |
| pSelect100 | 125 | +/-6.01 | 1/802,998 | +/-6.01E-08 |
| no plasmid ^1^ | 5.5 | +/-8.41 | 1/160,000,000 | +/-8.20E-09 |

^1^for the no plasmid transformation, the average number of resistant colonies per 100 000 000 cells and the rate of spontaneous resistance occurring is shown.
