# Supplementary material for: Genome, Functional Gene Annotation, and Nuclear Transformation of the Heterokont Oleaginous Alga Nannochloropsis oceanica CCMP1779
Source: PLoS Genet. 2012 Nov 15;8(11):e1003064. doi: 10.1371/journal.pgen.1003064 (PMC3499364; doi:10.1371/journal.pgen.1003064)
Supplement: Table S4 — Enriched GO categories in up- and down-regulated genes during N-deprived versus N-replete conditions based on RNAseq data. (DOCX) [file pgen.1003064.s017.docx]

**Table S4**. Enriched GO categories in up- and down-regulated genes during N-deprived versus N-replete conditions based on RNAseq data.

| \| **GO** \| **GO R^a^** \| **No GO R^b^** \| **GO U^c^** \| **No GO U^d^** \| **P^e^** \| **Annotation** \| \| --- \| --- \| --- \| --- \| --- \| --- \| --- \| \| **down-regulated** \|  \|  \|  \|  \|  \|  \| \| GO:0006096 bp^f^ \| 20 \| 645 \| 38 \| 5237 \| 2.15E-06 \| glycolysis \| \| GO:0015979 bp \| 10 \| 655 \| 7 \| 5268 \| 2.70E-06 \| photosynthesis \| \| GO:0015995 bp \| 9 \| 656 \| 5 \| 5270 \| 3.12E-06 \| chlorophyll biosynthetic process \| \| GO:0006094 bp \| 19 \| 646 \| 42 \| 5233 \| 2.07E-05 \| gluconeogenesis \| \| GO:0006012 bp \| 8 \| 657 \| 6 \| 5269 \| 3.84E-05 \| galactose metabolic process \| \| GO:0010007 cc^g^ \| 5 \| 660 \| 1 \| 5274 \| 9.45E-05 \| magnesium chelatase complex \| \| GO:0016851 mf^h^ \| 5 \| 660 \| 1 \| 5274 \| 9.45E-05 \| magnesium chelatase activity \| \| GO:0000084 bp \| 8 \| 657 \| 8 \| 5267 \| 0.000134 \| S phase of mitotic cell cycle \| \| GO:0015976 bp \| 12 \| 653 \| 23 \| 5252 \| 0.000256 \| carbon utilization \| \| GO:0000216 bp \| 7 \| 658 \| 7 \| 5268 \| 0.000361 \| M/G1 transition of mitotic cell cycle \| \| GO:0006098 bp \| 11 \| 654 \| 22 \| 5253 \| 0.000608 \| pentose-phosphate shunt \| \| GO:0006888 bp \| 7 \| 658 \| 9 \| 5266 \| 0.000984 \| ER to Golgi vesicle-mediated transport \| \| GO:0003980 mf \| 3 \| 662 \| 0 \| 5275 \| 0.001398 \| UDP-glucose:glycoprotein glucosyltransferase activity \| \| GO:0009773 bp \| 3 \| 662 \| 0 \| 5275 \| 0.001398 \| photosynthetic electron transport in photosystem I \| \| GO:0042132 mf \| 3 \| 662 \| 0 \| 5275 \| 0.001398 \| fructose 1,6-bisphosphate 1-phosphatase activity \| \| GO:0030604 mf \| 3 \| 662 \| 0 \| 5275 \| 0.001398 \| 1-deoxy-D-xylulose-5-phosphate reductoisomerase activity \| \| GO:0006000 bp \| 8 \| 657 \| 14 \| 5261 \| 0.001805 \| fructose metabolic process \| \| GO:0006271 bp \| 4 \| 661 \| 2 \| 5273 \| 0.00194 \| DNA strand elongation involved in DNA replication \| \| GO:0005985 bp \| 6 \| 659 \| 8 \| 5267 \| 0.00262 \| sucrose metabolic process \| \| GO:0006694 bp \| 7 \| 658 \| 12 \| 5263 \| 0.00321 \| steroid biosynthetic process \| \| GO:0030127 cc \| 4 \| 661 \| 3 \| 5272 \| 0.004127 \| COPII vesicle coat \| \| GO:0003755 mf \| 9 \| 656 \| 21 \| 5254 \| 0.004272 \| peptidyl-prolyl cis-trans isomerase activity \| \| GO:0006270 bp \| 5 \| 660 \| 6 \| 5269 \| 0.004493 \| DNA-dependent DNA replication initiation \| \| GO:0007018 bp \| 10 \| 655 \| 26 \| 5249 \| 0.004883 \| microtubule-based movement \| \| GO:0070402 mf \| 3 \| 662 \| 1 \| 5274 \| 0.005123 \| NADPH binding \| \| GO:0042277 mf \| 3 \| 662 \| 1 \| 5274 \| 0.005123 \| peptide binding \| \| GO:0019872 bp \| 5 \| 660 \| 7 \| 5268 \| 0.007 \| streptomycin biosynthetic process \| \| **up-regulated** \|  \|  \|  \|  \|  \|  \| \| GO:0009308 bp \| 5 \| 111 \| 1 \| 5823 \| 1.54E-08 \| amine metabolic process \| \| GO:0008131 mf \| 5 \| 111 \| 1 \| 5823 \| 1.54E-08 \| primary amine oxidase activity \| \| GO:0048038 mf \| 5 \| 111 \| 3 \| 5821 \| 1.39E-07 \| quinone binding \| \| GO:0005507 mf \| 5 \| 111 \| 23 \| 5801 \| 0.000179 \| copper ion binding \| \| GO:0008146 mf \| 3 \| 113 \| 7 \| 5817 \| 0.000788 \| sulfotransferase activity \| \| GO:0004190 mf \| 3 \| 113 \| 13 \| 5811 \| 0.003376 \| aspartic-type endopeptidase activity \| \| GO:0016887 mf \| 7 \| 109 \| 94 \| 5730 \| 0.003423 \| ATPase activity \| |
| --- | --- | --- | --- | --- | --- | --- | --- | --- | --- | --- | --- | --- | --- | --- | --- | --- | --- | --- | --- | --- | --- | --- | --- | --- | --- | --- | --- | --- | --- | --- | --- | --- | --- | --- | --- | --- | --- | --- | --- | --- | --- | --- | --- | --- | --- | --- | --- | --- | --- | --- | --- | --- | --- | --- | --- | --- | --- | --- | --- | --- | --- | --- | --- | --- | --- | --- | --- | --- | --- | --- | --- | --- | --- | --- | --- | --- | --- | --- | --- | --- | --- | --- | --- | --- | --- | --- | --- | --- | --- | --- | --- | --- | --- | --- | --- | --- | --- | --- | --- | --- | --- | --- | --- | --- | --- | --- | --- | --- | --- | --- | --- | --- | --- | --- | --- | --- | --- | --- | --- | --- | --- | --- | --- | --- | --- | --- | --- | --- | --- | --- | --- | --- | --- | --- | --- | --- | --- | --- | --- | --- | --- | --- | --- | --- | --- | --- | --- | --- | --- | --- | --- | --- | --- | --- | --- | --- | --- | --- | --- | --- | --- | --- | --- | --- | --- | --- | --- | --- | --- | --- | --- | --- | --- | --- | --- | --- | --- | --- | --- | --- | --- | --- | --- | --- | --- | --- | --- | --- | --- | --- | --- | --- | --- | --- | --- | --- | --- | --- | --- | --- | --- | --- | --- | --- | --- | --- | --- | --- | --- | --- | --- | --- | --- | --- | --- | --- | --- | --- | --- | --- | --- | --- | --- | --- | --- | --- | --- | --- | --- | --- | --- | --- | --- | --- | --- | --- | --- | --- | --- | --- | --- | --- | --- | --- | --- | --- | --- | --- | --- | --- | --- | --- | --- | --- | --- | --- | --- | --- | --- |
|  |

^a^ GO R, Number of significantly up- or down-regulated (R) genes with the GO annotation in question.

^b^ No GO R, Number of significantly up- or down-regulated genes without the GO annotation.

^c^ GO U, Number of genes without significant expression change with the GO annotation.

^d^ No GO U, Number of genes with no significant expression change that do not have the GO annotation.

^e^ Fisher’s exact test P value.

^f^ bp, Biological process.

^g^ cc, Cellular component.

^h^ mf, Molecular function.
