# Supplementary material for: Genome, Functional Gene Annotation, and Nuclear Transformation of the Heterokont Oleaginous Alga Nannochloropsis oceanica CCMP1779
Source: PLoS Genet. 2012 Nov 15;8(11):e1003064. doi: 10.1371/journal.pgen.1003064 (PMC3499364; doi:10.1371/journal.pgen.1003064)
Supplement: Table S6 — Enriched GO categories in conserved OGs and N. oceanica CCMP1779-specific and N. gaditana-specific genes. (DOCX) [file pgen.1003064.s019.docx]

**Table S6:** Enriched GO categories in conserved OGs and *N. oceanica* CCMP1779*-*specific and *N. gaditana*-specific genes.

| **GO** | **GO G** | **GO No G** | **No GO G** | **No GO No G** | **p** | **Annotation** |
| --- | --- | --- | --- | --- | --- | --- |
| **Conserved OGs** | | | | | | |
| GO:0006412 bp | 89 | 21 | 3288 | 2582 | 8.6E-08 | translation |
| GO:0009507 cc | 112 | 33 | 3265 | 2570 | 1.6E-07 | chloroplast |
| GO:0003735 mf | 90 | 24 | 3287 | 2579 | 4.8E-07 | structural constituent of ribosome |
| GO:0005739 cc | 157 | 61 | 3220 | 2542 | 1.9E-06 | mitochondrion |
| GO:0042254 bp | 92 | 31 | 3285 | 2572 | 2.9E-05 | ribosome biogenesis |
| GO:0005840 cc | 95 | 33 | 3282 | 2570 | 3.0E-05 | ribosome |
| GO:0015979 bp | 17 | 0 | 3360 | 2603 | 7.6E-05 | photosynthesis |
| GO:0005743 cc | 32 | 6 | 3345 | 2597 | 4.2E-04 | mitochondrial inner membrane |
| GO:0005737 cc | 206 | 113 | 3171 | 2490 | 3.0E-03 | cytoplasm |
| GO:0006977 bp | 11 | 0 | 3366 | 2603 | 3.5E-03 | DNA damage response, signal transduction by p53 class mediator resulting in cell cycle arrest |
| GO:0006098 bp | 27 | 6 | 3350 | 2597 | 4.1E-03 | pentose-phosphate shunt |
| GO:0055114 bp | 285 | 170 | 3092 | 2433 | 5.9E-03 | oxidation-reduction process |
| GO:0015992 bp | 25 | 6 | 3352 | 2597 | 6.1E-03 | proton transport |
| GO:0051603 bp | 9 | 0 | 3368 | 2603 | 6.4E-03 | proteolysis involved in cellular protein catabolic process |
| GO:0016117 bp | 9 | 0 | 3368 | 2603 | 6.4E-03 | carotenoid biosynthetic process |
| GO:0008565 mf | 20 | 4 | 3357 | 2599 | 7.1E-03 | protein transporter activity |
| GO:0004298 mf | 21 | 4 | 3356 | 2599 | 7.2E-03 | threonine-type endopeptidase activity |
| GO:0051536 mf | 37 | 12 | 3340 | 2591 | 8.5E-03 | iron-sulfur cluster binding |
| GO:0006118 bp | 94 | 46 | 3283 | 2557 | 9.7E-03 | electron transport |
| ***N. oceanica* specific genes** | | | | | | |
| GO:0004871 mf | 15 | 18 | 1071 | 4876 | 2.7E-04 | signal transducer activity |
| GO:0008131 mf | 5 | 1 | 1081 | 4893 | 1.0E-03 | primary amine oxidase activity |
| GO:0009308 bp | 5 | 1 | 1081 | 4893 | 1.0E-03 | amine metabolic process |
| GO:0046429 mf | 4 | 0 | 1082 | 4894 | 1.1E-03 | 4-hydroxy-3-methylbut-2-en-1-yl diphosphate synthase activity |
| GO:0030604 mf | 3 | 0 | 1083 | 4894 | 6.0E-03 | 1-deoxy-D-xylulose-5-phosphate reductoisomerase activity |
| GO:0004375 mf | 3 | 0 | 1083 | 4894 | 6.0E-03 | glycine dehydrogenase (decarboxylating) activity |
| GO:0005622 cc | 42 | 114 | 1044 | 4780 | 6.0E-03 | intracellular |
| GO:0048038 mf | 5 | 3 | 1081 | 4891 | 6.7E-03 | quinone binding |
| GO:0003989 mf | 6 | 5 | 1080 | 4889 | 7.1E-03 | acetyl-CoA carboxylase activity |
| GO:0003676 mf | 64 | 197 | 1022 | 4697 | 8.4E-03 | nucleic acid binding |
| ***N. gaditana s*pecific genes** | | | | | | |
| GO:0009507 cc | 25 | 107 | 228 | 2648 | 7.31E-05 | chloroplast |
| GO:0016791 mf | 5 | 2 | 248 | 2753 | 7.39E-05 | phosphatase activity |
| GO:0019843 mf | 7 | 10 | 246 | 2745 | 2.56E-04 | rRNA binding |
| GO:0016730 mf | 3 | 0 | 250 | 2755 | 5.89E-04 | oxidoreductase activity, acting on iron-sulfur proteins as donors |
| GO:0019685 bp | 3 | 0 | 250 | 2755 | 5.89E-04 | photosynthesis, dark reaction |
| GO:0005840 cc | 11 | 38 | 242 | 2717 | 1.95E-03 | ribosome |
| GO:0036068 bp | 3 | 1 | 250 | 2754 | 2.21E-03 | light-independent chlorophyll biosynthetic process |
| GO:0009535 cc | 11 | 42 | 242 | 2713 | 3.76E-03 | chloroplast thylakoid membrane |
| GO:0031347 bp | 2 | 0 | 251 | 2755 | 7.05E-03 | regulation of defense response |
| GO:0009539 cc | 2 | 0 | 251 | 2755 | 7.05E-03 | photosystem II reaction center |
| GO:0030071 bp | 2 | 0 | 251 | 2755 | 7.05E-03 | regulation of mitotic metaphase/anaphase transition |
| GO:0004722 mf | 3 | 3 | 250 | 2752 | 9.71E-03 | protein serine/threonine phosphatase activity |
| GO:0009772 bp | 3 | 3 | 250 | 2752 | 9.71E-03 | photosynthetic electron transport in photosystem II |
| GO:0030076 cc | 3 | 3 | 250 | 2752 | 9.71E-03 | light-harvesting complex |
| GO:0009982 mf | 3 | 3 | 250 | 2752 | 9.71 E-03 | pseudouridine synthase activity |

GO G, Number of genes with the GO annotation in the conserved OGs or species- specific groups of genes.

No GO G, Number of genes without the GO annotation in conserved OGs or species- specific groups of genes.

GO No G, Number of genes with the GO annotation not in conserved OGs or species- specific groups of genes.

No GO No G, Number of genes without the GO annotation not in conserved OGs or species- specific groups of genes.

p Fisher’s exact test P value.

bp, Biological process.

mf, molecular function.

cc, Cellular component.
