# Supplementary material for: Genome, Functional Gene Annotation, and Nuclear Transformation of the Heterokont Oleaginous Alga Nannochloropsis oceanica CCMP1779
Source: PLoS Genet. 2012 Nov 15;8(11):e1003064. doi: 10.1371/journal.pgen.1003064 (PMC3499364; doi:10.1371/journal.pgen.1003064)
Supplement: Table S7 — Putative genes identified to be involved in photosynthetic electron transport in CCMP1779. In cases where no gene model was structurally annotated, genome coordinates are given. (DOCX) [file pgen.1003064.s020.docx]

**Table S7:** Putative genes identified to be involved in photosynthetic electron transport in CCMP1779. In cases where no gene model was structurally annotated, genome coordinates are given.

| **Description** | **Gene Name** | **ID** |
| --- | --- | --- |
| **Cytochrome b6/f Complex** |  |  |
| Rieske Fe-S subunit of Cytochrome b6-f Complex | PETC | CCMP1779_1077-mRNA-1 |
| Cytochrome b6-f Complex subunit IV | PETD1 | nanno_2528:785..1264 ^1,2^ |
|  | PETD2 | nanno_6709:10149..10859 ^2^ |
| Cytochrome b6-f Complex subunit | PETM | nanno_24:8196..7864^1,2^ |
| Cytochrome b6-f Complex subunit VIII | PETN | Not Found |
| **Soluble Electron Carriers** |  |  |
| Plastocyanin | PETE1 | nanno_963:17795..18553 ^2^ |
| Ferredoxin | PETF1 | NannoCCMP1779_7894-mRNA-1 |
|  | PETF2 | NannoCCMP1779_3054-mRNA-1 |
|  | PETF3 | NannoCCMP1779_2102-mRNA-1 |
| Cytochrome c553 |  | Not Found |
| Ferredoxin-NADP oxidoreductase | FNR1 | nanno_1036:17495..20160 ^2^ |
|  | FNR2 | nanno_427:12789..11620 ^2^ |
|  | FNR3 | snap_masked-nanno_87-abinit-gene-0.5 ^4^ |
|  | FNR4 | NannoCCMP1779_2013-mRNA-1 |
|  | FNR5 | augustus_masked-nanno_3186-abinit-gene-0.3 ^4^ |
|  | FNR6 | CCMP1779_5041-mRNA-1 |
| **Photosystem I** |  |  |
| PSI subunit II | PSAD1 | nanno_727:57421..58384 ^2^ |
|  | PSAD2 | NannoCCMP1779_1336-mRNA-1 |
| PSI subunit IV | PSAE | nanno_2655:6951..6687 ^2^ |
| PSI subunit III | PSAF | nanno_2570:15982..19244 ^2^ |
| PSI subunit V | PSAG | Not Found |
| PSI subunit VI | PSAH | nanno_798:86361..86944 ^2^ |
| PSI subunit X |  | Not Found |
| PSI subunit XI | PSAL | nanno_1707:204..719 ^2^ |
| PSI subunit N | PSAN | nanno_812:37141..36722 ^2^ |
| **Photosystem II** |  |  |
| PSII subunit M | PSBM | NannoCCMP1779_10463-mRNA-1 |
| PSII Manganese-Stabilizing Protein | PSBO | augustus_masked-nanno_6323-abinit-gene-0.1^4^ |
| PSII Oxygen-Evolving Complex 23 | PSBP | NannoCCMP1779_11420-mRNA-1 |
| PSII Oxygen-Evolving Complex Enhancer Protein 3 | PSBQ | Not Found |
| PSII subunit R | PSBR | Not Found |
| PSII 12KDa Extrinsic Protein | PSBU | CCMP1779_6260-mRNA-1 |
| PSII 13KDa Protein | PSBW | CCMP1779_10160-mRNA-1 |
| PSII subunit X | PSBX | nanno_243:28102..28984 ^2,3^ |
| Thylakoid Lumenal 17.4KDa Protein | p17.4 | CCMP1779_4019-mRNA-1 |
| **ATP Synthase** |  |  |
| ATP Synthase CF1 γ chain | ATPC1 | augustus_masked-nanno_5244-abinit-gene-0.0 ^4^ |
|  | ATPC2 | NannoCCMP1779_6999-mRNA-1 |
|  | ATPC3 | augustus_masked-nanno_1453-abinit-gene-0.0 ^4^ |
| ATP Synthase CF1 δ subunit | ATPD1 | NannoCCMP1779_10955-mRNA-1 |
|  | ATPD2 | nanno_6106:166..811 ^2^ |
| ATP Synthase CF0 subunit B' | ATPG | Not Found |

^1^ partial sequence,

^2^ ambiguous structural annotation, coordinates are given (contigID:start..stop)

^3^ ambiguous functional annotation,

^4^ this gene model is from augustus or snap gene annotation and was found superior to the final maker annotation after manual examination
