# Supplementary material for: Genome, Functional Gene Annotation, and Nuclear Transformation of the Heterokont Oleaginous Alga Nannochloropsis oceanica CCMP1779
Source: PLoS Genet. 2012 Nov 15;8(11):e1003064. doi: 10.1371/journal.pgen.1003064 (PMC3499364; doi:10.1371/journal.pgen.1003064)
Supplement: Table S8 — Genes predicted to encode for Violaxanthin-Chlorophyll binding proteins (VCP) in CCMP1779 genome and there designation in the phylogenetic tree (Fig. 6). (DOCX) [file pgen.1003064.s021.docx]

**Table S8:** Genes predicted to encode for Violaxanthin-Chlorophyll binding proteins (VCP) in CCMP1779 genome and there designation in the phylogenetic tree (Fig. 6).

| **Name in Tree**  **(Fig. 6)** | **ID** | **LHC-Type** |
| --- | --- | --- |
| 1 | CCMP1779_8367-mRNA-1 | F-type |
| 2 | CCMP1779_4699-mRNA-1 | F-type |
| 3 | CCMP1779_3700-mRNA-1 | R-type |
| 4 | CCMP1779_10562-mRNA-1 | R-type |
| 5 | CCMP1779_10564-mRNA-1 | F-type |
| 6 | CCMP1779_9414-mRNA-1 | R-type |
| 7 | CCMP1779_11954-mRNA-1 | R-type |
| 8 | CCMP1779_6809-mRNA-1 | LHCSR-like |
| 9 | CCMP1779_6828-mRNA-1 | R-type |
| 10 | CCMP1779_11487-mRNA-1 | R-type |
| 11 | CCMP1779_8196-mRNA-1 | R-type |
| 12 | CCMP1779_11886-mRNA-1 | R-type |
| 13 | CCMP1779_8305-mRNA-1 | R-type |
| 14 | CCMP1779_6226-mRNA-1 | LHCSR-like |
| 15 | CCMP1779_35-mRNA-1 | R-type |
| 16 | CCMP1779_904-mRNA-1 | R-type |
| 17 | CCMP1779_10420-mRNA-1 | R-type |
| 18 | CCMP1779_5333-mRNA-1 | R-type |
| 19 | CCMP1779_6137-mRNA-1 | F-type |
| 20 | nanno_220:23958..25565 ^1^ | R-type |

^1^ ambiguous structural annotation, genome coordinates given
