# Supplementary material for: Genome, Functional Gene Annotation, and Nuclear Transformation of the Heterokont Oleaginous Alga Nannochloropsis oceanica CCMP1779
Source: PLoS Genet. 2012 Nov 15;8(11):e1003064. doi: 10.1371/journal.pgen.1003064 (PMC3499364; doi:10.1371/journal.pgen.1003064)
Supplement: Table S9 — Putative genes identified to be involved in xanthophyll synthesis. (DOCX) [file pgen.1003064.s022.docx]

**Table S9:** Putative genes identified to be involved in xanthophyll synthesis

| **Description** | **Gene Name** | **ID** |
| --- | --- | --- |
| **Carotenoid Biosysnthesis** |  |  |
| Geranylgeranyl Pyrophosphate Synthase | GGPS1 | CCMP1779_9014-mRNA-1 |
|  | GGPS2 | CCMP1779_378-mRNA-1 |
|  | GGPS3 | CCMP1779_3156-mRNA-1 |
|  | GGPS4 | CCMP1779_3532-mRNA-1 |
| Phytoene Syntase | PSY | CCMP1779_10288-mRNA-1 |
| Phytoene Desaturase | PDS | CCMP1779_4801-mRNA-1 |
| ζ-Carotene Isomerase | ZISO | maker-nanno_856-snap-gene-0.25-mRNA-1 ^1^ |
| ζ-Carotene Desaturase | ZDS | CCMP1779_501-mRNA-1 |
| Carotene Isomerase |  | Not found |
| Lycopene β-Cyclase | LCYB | CCMP1779_4993-mRNA1 |
| Di-iron Carotene Hydroxylase |  | Not found |
| Zeaxanthin Epoxidase | ZEP | CCMP1779_6822-mRNA-1 |
| Violaxanthin DeEpoxidase | VDE ^2^ | CCMP1779_11477-mRNA-1  CCMP1779_11475-mRNA-1 |
| Neoxanthin Synthase | NXS | CCMP1779_11764-mRNA-1 |

^1^ this gene model is from augustus or snap gene annotation and was found superior to the final maker annotation after manual examination,

^2^ gene on two contigs, incomplete model
