# Supplementary material for: Genome, Functional Gene Annotation, and Nuclear Transformation of the Heterokont Oleaginous Alga Nannochloropsis oceanica CCMP1779
Source: PLoS Genet. 2012 Nov 15;8(11):e1003064. doi: 10.1371/journal.pgen.1003064 (PMC3499364; doi:10.1371/journal.pgen.1003064)
Supplement: Table S10 — Genes putatively involved in central carbon metabolism and possible carbon concentrating mechanism. (DOCX) [file pgen.1003064.s023.docx]

**Table S10:** Genes putatively involved in central carbon metabolism and possible carbon concentrating mechanism.

| **Description** | **Name** | | | **ID** | |  |
| --- | --- | --- | --- | --- | --- | --- |
| **GLYCOLYSIS** | | | | | |  |
| Glucokinase | HK | | | CCMP1779_11432-mRNA-1 | |  |
| Glucokinase | HK | | | CCMP1779_11432-mRNA-1 | |  |
| Phosphoglucoisomerase | PGI | | | CCMP1779_11835-mRNA-1 | |  |
| Phosphoglucoisomerase | PGI | | | CCMP1779_2243-mRNA-1 | |  |
| Phosphofructokinase | PFK | | | CCMP1779_6373-mRNA-1 | |  |
| Phosphofructokinase | PFK | | | CCMP1779_10476-mRNA-1 | |  |
| Phosphofructokinase | PFK | | | CCMP1779_5677-mRNA-1 | |  |
| Fructosebisphosphate Aldolase | Glycolytic | | | CCMP1779_135-mRNA-1 | |  |
| Fructosebisphosphate Aldolase | Glycolytic | | | CCMP1779_567-mRNA-1 | |  |
| Fructosebisphosphate Aldolase | Glycolytic | | | CCMP1779_7930-mRNA-1 | |  |
| Triose phosphate isomerase | TIM | | | CCMP1779_3017-mRNA-1 | |  |
| Triose phosphate isomerase | TIM | | | CCMP1779_3017-mRNA-1 | |  |
| Triose phosphate isomerase | TIM | | | CCMP1779_10440-mRNA-1 | |  |
| Triose phosphate isomerase | TIM | | | CCMP1779_5281-mRNA-1 | |  |
| Glyceraldehydephosphate dehydrogenase | G3PDH | | | CCMP1779_2231-mRNA-1 | |  |
| Glyceraldehydephosphate dehydrogenase | G3PDH | | | CCMP1779_4900-mRNA-1 | |  |
| Glyceraldehydephosphate dehydrogenase | G3PDH | | | CCMP1779_3236-mRNA-1 | |  |
| Phosphoglycerokinase | PGK | | | CCMP1779_9105-mRNA-1 | |  |
| Phosphoglycerokinase | PGK | | | CCMP1779_3273-mRNA-1 | |  |
| Phosphoglycerokinase | PGK | | | CCMP1779_3273-mRNA-1 | |  |
| Phosphoglycerokinase | PGK | | | CCMP1779_4644-mRNA-1 | |  |
| Phosphoglycerate mutase | PGM | | | CCMP1779_7187-mRNA-1 | |  |
| Phosphoglycerate mutase | PGM | | | CCMP1779_7012-mRNA-1 | |  |
| Phosphoglycerate mutase | PGM | | | CCMP1779_1942-mRNA-1 | |  |
| Phosphoglycerate mutase | PGM | | | CCMP1779_7187-mRNA-1 | |  |
| Phosphoglycerate mutase | PGM | | | CCMP1779_4572-mRNA-1 | |  |
| Phosphoglycerate mutase | PGM | | | CCMP1779_214-mRNA-1 | |  |
| Enolase | Enolase | | | CCMP1779_7147-mRNA-1 | |  |
| Enolase | Enolase | | | CCMP1779_7147-mRNA-1 | |  |
| Pyruvate Kinase | PK | | | CCMP1779_497-mRNA-1 | |  |
| Pyruvate Kinase | PK | | | CCMP1779_5759-mRNA-1 | |  |
| Pyruvate Kinase | PK | | | CCMP1779_10510-mRNA-1 | |  |
| Pyruvate Kinase | PK | | | CCMP1779_8741-mRNA-1 | |  |
| Phosphoenolpyruvate Carboxylase | PEPcase | | | CCMP1779_3970-mRNA-1 | |  |
| Carbonic Anhydrase | Pro_CA | | | CCMP1779_11263-mRNA-1 | |  |
| Carbonic Anhydrase | Pro_CA | | | CCMP1779_6698-mRNA-1 | |  |
| **GLUCONEOGENESIS** | | | | | |  |
| Fructose-1,6-bisphosphatase | FBPase | | | CCMP1779_1856-mRNA-1 | |  |
| Fructose-1,6-bisphosphatase | FBPase | | | CCMP1779_7187-mRNA-1 | |  |
| Fructose-1,6-bisphosphatase | FBPase | | | CCMP1779_5277-mRNA-1 | |  |
| Fructose-1,6-bisphosphatase | FBPase | | | CCMP1779_1856-mRNA-1 | |  |
| Fructose-1,6-bisphosphatase | FBPase | | | CCMP1779_4791-mRNA-1 | |  |
| Phosphoenolpyruvate Carboxykinase | PEPCK | | | CCMP1779_6030-mRNA-1 | |  |
| Glucose-6-phosphatase | G6PC | | | CCMP1779_6325-mRNA-1 | |  |
| Pyruvate Carboxylase | PC | | | CCMP1779_6175-mRNA-1 | |  |
| Pyruvate Carboxylase | PC | | | CCMP1779_7693-mRNA-1 | |  |
| Pyruvate Carboxylase | PC | | | CCMP1779_4504-mRNA-1 | |  |
| Pyruvate Carboxylase | PC | | | CCMP1779_7838-mRNA-1 | |  |
| **TCA CYCLE** | | | | | |  |
| Pyruvate Dehydrogenase | E1 | | CCMP1779_6658-mRNA-1 | |  |  |
| Pyruvate Dehydrogenase | E1 | | CCMP1779_667-mRNA-1 | |  |  |
| dihydrolipoyl acetyltransferase | E2 | | CCMP1779_1097-mRNA-1 | |  |  |
| dihydrolipoyl acetyltransferase | E2 | | CCMP1779_7562-mRNA-1 | |  |  |
| dihydrolipoyl acetyltransferase | E2 | | CCMP1779_6573-mRNA-1 | |  |  |
| dihydrolipoyl dehydrogenase | E3 | | CCMP1779_196-mRNA-1 | |  |  |
| Pyruvate Dehydrogenase kinase | PDK | | CCMP1779_9282-mRNA-1 | |  |  |
| Pyruvate Dehydrogenase kinase | PDK | | CCMP1779_9282-mRNA-1 | |  |  |
| Citrate Synthase | CS | | CCMP1779_10757-mRNA-1 | |  |  |
| Citrate Synthase | CS | | CCMP1779_10051-mRNA-1 | |  |  |
| Citrate Synthase | CS | | CCMP1779_7201-mRNA-1 | |  |  |
| Aconitase | Aconitase | | CCMP1779_9186-mRNA-1 | |  |  |
| Isocitrate Dehydrogenase | IDH | | CCMP1779_3495-mRNA-1 | |  |  |
| Alphaketoglutarate Dehydrogenase | OGDH | | CCMP1779_826-mRNA-1 | |  |  |
| Alphaketoglutarate Dehydrogenase | OGDH | | CCMP1779_826-mRNA-1 | |  |  |
| Dihydrolipoyl Succinyltransferase | DLST | | CCMP1779_1597-mRNA-1 | |  |  |
| Succinyl CoA Synthatase | SCS | | CCMP1779_8141-mRNA-1 | |  |  |
| Succinate Dehydrogenase | SQR | | CCMP1779_9122-mRNA-1 | |  |  |
| Succinate Dehydrogenase | SQR | | CCMP1779_3454-mRNA-1 | |  |  |
| Succinate Dehydrogenase | SQR | | CCMP1779_10056-mRNA-1 | |  |  |
| Fumerase | Fumarase | | CCMP1779_9178-mRNA-1 | |  |  |
| Fumerase | Fumarase | | CCMP1779_9178-mRNA-1 | |  |  |
| Malate Dehydrogenase | MDH | | CCMP1779_4869-mRNA-1 | |  |  |
| Malate Dehydrogenase | MDH | | CCMP1779_7762-mRNA-1 | |  |  |
| Malate Dehydrogenase | MDH | | CCMP1779_2456-mRNA-1 | |  |  |
| **OPPP and RPPP** | | | | | |  |
| Glucose-6-phosphate dehydrogenase | G6PD | CCMP1779_640-mRNA-1 | | |  |  |
| Glucose-6-phosphate dehydrogenase | G6PD | CCMP1779_2822-mRNA-1 | | |  |  |
| 6-phosphogluconate dehydrogenase | 6PGD | CCMP1779_734-mRNA-1 | | |  |  |
| 6-phosphogluconate dehydrogenase | 6PGD | CCMP1779_6395-mRNA-1 | | |  |  |
| 6-phosphogluconate dehydrogenase | 6PGD | CCMP1779_2147-mRNA-1 | | |  |  |
| 6-phosphogluconate dehydrogenase | 6PGD | CCMP1779_11127-mRNA-1 | | |  |  |
| Ribose 5-phosphate isomerase | Rpi | CCMP1779_5917-mRNA-1 | | |  |  |
| Ribulose-phosphate Epimerase | Ribul_P_3_epim | CCMP1779_2948-mRNA-1 | | |  |  |
| Ribulose-phosphate Epimerase | Ribul_P_3_epim | CCMP1779_5327-mRNA-1 | | |  |  |
| Transketolase | TKT | CCMP1779_6748-mRNA-1 | | |  |  |
| Transketolase | TKT | CCMP1779_852-mRNA-1 | | |  |  |
| Transketolase | TKT | CCMP1779_667-mRNA-1 | | |  |  |
| Transaldolase | TALDO1 | CCMP1779_7418-mRNA-1 | | |  |  |
| Transaldolase | TALDO2 | CCMP1779_6166-mRNA-1 | | |  |  |
| RuBisco Small Subunit | RuBisCO_small | CCMP1779_10920-mRNA-1 | | |  |  |
| RuBisco Large Subunit | RuBisCO_large | CCMP1779_6968-mRNA-1 | | |  |  |
| RuBisco Small Subunit | RuBisCO_small | CCMP1779_10920-mRNA-1 | | |  |  |
| RuBisco Large Subunit | RuBisCO_large | CCMP1779_4877-mRNA-1 | | |  |  |
| RuBisco Large Subunit | RuBisCO_large | CCMP1779_11329-mRNA-1 | | |  |  |
| phosphoglycerate kinase | PGK | CCMP1779_3273-mRNA-1 | | |  |  |
| phosphoglycerate kinase | PGK | CCMP1779_9105-mRNA-1 | | |  |  |
| Phosphoglycerate kinase | PGK | CCMP1779_3273-mRNA-1 | | |  |  |
| Phosphoglycerate kinase | PGK | CCMP1779_4644-mRNA-1 | | |  |  |
| Glyceraldehyde 3-phosphate dehydrogenase | GAPDH | CCMP1779_2231-mRNA-1 | | |  |  |
| Glyceraldehyde 3-phosphate dehydrogenase | GAPDH | CCMP1779_4900-mRNA-1 | | |  |  |
| Glyceraldehyde 3-phosphate dehydrogenase | GAPDH | CCMP1779_3236-mRNA-1 | | |  |  |
| Phosphoribulokinase | PRK | CCMP1779_11364-mRNA-1 | | |  |  |
| Phosphoribulokinase | PRK | CCMP1779_9418-mRNA-1 | | |  |  |
| gluconolactonase | GNL | CCMP1779_1682-mRNA-1 | | |  |  |
| Sedoheptulose bisphosphatase | SBPase | CCMP1779_2947-mRNA-1 | | |  |  |
| Sedoheptulose bisphosphatase | SBPase | CCMP1779_4791-mRNA-1 | | |  |  |
| **GLYOXYLATE CYCLE** | | | | | |  |
| Malate Synthase | Malate_synthase | CCMP1779_6945-mRNA-1 | | | |  |
| Malate Synthase | Malate_synthase | CCMP1779_11836-mRNA-1 | | | |  |
| Isocitrate Lyase | ICL | CCMP1779_1574-mRNA-1 | | |  |  |
| Isocitrate Lyase | ICL | CCMP1779_4636-mRNA-1 | | |  |  |
| alanine-glyoxylate aminotransferase | AGT | CCMP1779_9944-mRNA-1 | | |  |  |
| alanine-glyoxylate aminotransferase | AGT | CCMP1779_3460-mRNA-1 | | |  |  |
| glycine decarboxylase | GLDC | CCMP1779_6047-mRNA-1 | | |  |  |
| glycine decarboxylase | GLDC | CCMP1779_5912-mRNA-1 | | |  |  |
| glycine dehydrogenase | GLDC | CCMP1779_2342-mRNA-1 | | |  |  |
|  | | | | | | |
| **CCM related genes** | | | | | | |
| α-type carbonic anhydrase | CAH1 | CCMP1779_6698-mRNA-1 | | | | |
| β-type carbonic anhydrase | CAH2 | CCMP1779_11263-mRNA-1 | | | | |
| chloroplast envelope located, low CO2 inducible proteins | CCP | CCMP1779_7325-mRNA-1 | | | | |
| Formate/Nitrite Transporter family, incuded under limiting-CO2 condition | LCIA | CCMP1779_6536-mRNA-1 | | | | |
|  | | | | | | |
| **C4 related genes** | | | | | | |
| Phosphoenolpyruvate carboxylase | PEPCase | CCMP1779_3970-mRNA-1 | | | | |
| Phosphoenolpyruvate carboxkinase | PEPCK | no homolog | | | | |
| Pyruvate phosphate dikinase | PPDK | CCMP1779_2768-mRNA-1 | | | | |
|  | PPDK | CCMP1779_1520-mRNA-1 | | | | |
| Malic enzyme | ME | CCMP1779_9004-mRNA-1 | | | | |
|  | ME | CCMP1779_4675-mRNA-1 | | | | |
| Malate dehydrogenase | MDH | CCMP1779_4869-mRNA-1 | | | | |
|  | MDH | CCMP1779_7762-mRNA-1 | | | | |
|  | MDH | CCMP1779_2456-mRNA-1 | | | | |
