# Supplementary material for: Genome, Functional Gene Annotation, and Nuclear Transformation of the Heterokont Oleaginous Alga Nannochloropsis oceanica CCMP1779
Source: PLoS Genet. 2012 Nov 15;8(11):e1003064. doi: 10.1371/journal.pgen.1003064 (PMC3499364; doi:10.1371/journal.pgen.1003064)
Supplement: Table S11 — Functional annotation of putative genes involved in H2 metabolism and oxidative phosphorylation identified in the CCMP1779 genome. (DOCX) [file pgen.1003064.s024.docx]

**Table S11:** Functional annotation of putative genes involved in H_2_ metabolism and oxidative phosphorylation identified in the CCMP1779 genome.

|  |  |  |
| --- | --- | --- |
| **Description** | **Name** | **ID** |
|  | | |
| **HYDROGEN** | | |
| [FeFe]-hydrogenase | HydA1 | CCMP1779_5967-mRNA-1 |
| [FeFe]-hydrogenase maturation factor HydG | HydG | augustus_masked-nanno_856-abinit-gene-0.2-mRNA-1 ^1^ |
| [FeFe]-hydrogenase maturation factor HydF | HydF | CCMP1779_5970-mRNA-1 |
| [FeFe]-hydrogenase maturation factor HydE | HydE | augustus_masked-nanno_856-abinit-gene-0.1-mRNA-1 ^1^ |
|  | | |
| **RESPIRATION** | | |
| succinate dehydrogenase flavoprotein subunit | Sdh2 | CCMP1779_10056-mRNA-1 |
| succinate dehydrogenase subunit A | Sdh1 | CCMP1779_10769-mRNA-1 |
| succinate dehydrogenase cytochrome b subunit | Sdh3 | CCMP1779_9122-mRNA-1 |
| succinate dehydrogenase subunit D | Sdh4 | CCMP1779_3454-mRNA-1 |
| heme O synthase | Cox10 | augustus_masked-nanno_1077-abinit-gene-1.10-mRNA-1 ^1^ |
| cytochrome *c* oxidase subunit 6b | Cox12 | augustus_masked-nanno_604-abinit-gene-0.21-mRNA-1 ^1^ |
| heme A synthase | Cox15 | CCMP1779_925-mRNA-1 |
| cytochrome *c* oxidase assembly protein | Cox11 | CCMP1779_4145-mRNA-1 |
| cytochrome *c* oxidase assembly protein | Shy1 | CCMP1779_22-mRNA-1 |
| cytochrome *c* oxidase assembly protein | Sco1 | CCMP1779_6751-mRNA-1 |
| cytochrome *c* oxidase copper chaperone | Cox17 | CCMP1779_9640-mRNA-1 |
| cytochrome *c* oxidase molecular chaperone/heat shock protein | Ssc1 | CCMP1779_8242-mRNA-1 |
| cytochrome *c* oxidase subunit II | Cox2 | CUFF.84573.1+ ^2^ |
| cytochrome *c* oxidase subunit I | Cox1 | CUFF.95393.1+ ^2^ |
| cytochrome *c* oxidase subunit III | Cox3 | CUFF.95359.1+ ^2^ |
| alternative oxidase 2 | Aox2 | augustus_masked-nanno_2785-abinit-gene-0.2-mRNA-1 ^1^ |
| alternative oxidase 2 | Aox1 | augustus_masked-nanno_1051-abinit-gene-0.9-mRNA-1 ^1^ |
| alternative oxidase 3 | Aox3 | augustus_masked-nanno_3659-abinit-gene-0.0-mRNA-1 ^1^ |
| NADH:ubiquinone oxidoreductase subunit 10 | Nuo10 | CCMP1779_10235-mRNA-1 |
| NADH:ubiquinone oxidoreductase subunit 17 | Nuo17 | CCMP1779_11866-mRNA-1 |
| NADH:ubiquinone oxidoreductase subunit E | NuoE | CCMP1779_9179-mRNA-1 |
| NADH:ubiquinone oxidoreductase subunit F | NuoF | CCMP1779_4574-mRNA-1 |
| NADH:ubiquinone oxidoreductase subunit I | NuoI | CCMP1779_7640-mRNA-1 |
| NADH:ubiquinone oxidoreductase subunit 9 | Nuo9 | CCMP1779_6889-mRNA-1 |
| NADH:ubiquinone oxidoreductase 18 kDa subunit | Nuo18k | CCMP1779_11055-mRNA-1 |
| NADH:ubiquinone oxidoreductase subunit B14 | NuoB14 | CCMP1779_6453-mRNA-1 |
| NADH:ubiquinone oxidoreductase subunit B16 | NuoB16 | CCMP1779_9016-mRNA-1 |
| NADH:ubiquinone oxidoreductase subunit B18 | NuoB18 | CCMP1779_211-mRNA-1 |
| NADH:ubiquinone oxidoreductase subunit 11 | Nuo11 | augustus_masked-nanno_224-abinit-gene-1.7-mRNA-1 ^1^ |
| NADH:ubiquinone oxidoreductase subunit S4 | NuoS4 | CCMP1779_2896-mRNA-1 |
| NADH:ubiquinone oxidoreductase subunit S5 | NuoS5 | CCMP1779_10934-mRNA-1 |
| NADH dehydrogenase subunit 4 | Nuo4 | CUFF.95343.1+ ^2^ |
| NADH dehydrogenase subunit 5 | Nuo5 | CUFF.84531.1+ ^2^ |
| NADH dehydrogenase subunit 1 | Nuo1 | CUFF.95403.1+ ^2^ |
| NADH:ubiquinone oxidoreductase subunit 7 | Nuo7 | CUFF.95391.1+ ^2^ |
| NADH dehydrogenase subunit 2 | Nuo2 | CUFF.95399.1+ ^2^ |
| NADH dehydrogenase subunit 3 | Nuo3 | CUFF.95361.1+ ^2^ |
| F1F0 ATP synthase gamma subunit | Atp3 | augustus_masked-nanno_5442-abinit-gene-0.2-mRNA-1 ^1^ |
| ATP synthase O subunit | Atp5C1 | CCMP1779_10955-mRNA-1 |
| F0F1 ATP synthase subunit alpha | Atp5A1 | CCMP1779_10718-mRNA-1 |
| F-type H-ATPase beta subunit | Atp5B | CCMP1779_9984-mRNA-1 |
| ATP synthase F0 subunit 6 | Atp6 | CUFF.95401.1+ ^2^ |
| ATP synthase mitochondrial F1 complex assembly factor 2 | AtpAF2 | CCMP1779_10134-mRNA-1 |
| ATP synthase mitochondrial F1 complex assembly factor 2 | AtpAF1 | CCMP1779_6914-mRNA-1 |
| ubiquinol cytochrome c reductase cytochrome c1 | CytC1 | CCMP1779_9301-mRNA-1 |
| ubiquinol cytochrome c reductase 14kDa subunit | Qcr7 | CCMP1779_9021-mRNA-1 |
| cytochrome b-c1 complex subunit Rieske | RipI | CCMP1779_8327-mRNA-1 |
| cytochrome b-c1 complex 50 kDa subunit 1 | Qcr1 | CCMP1779_635-mRNA-1 |
| Ubiquinol-cytochrome c reductase iron-sulfur subunit 1 | Risp1 | CCMP1779_8327-mRNA-1 |
| Cytochrome b | CytB | CUFF.95357.1+ ^2^ |
|  | | |
| **FERREDOXINS** | | |
| 2Fe-2S ferredoxin | Fdx | CCMP1779_688-mRNA-1 |
| ferredoxin, adrenodoxin-like protein | Mfdx1 | CCMP1779_10471-mRNA-1 |
| putative chloroplast ferredoxin 1 | Fdx1 | CCMP1779_7894-mRNA-1 |
| putative chloroplast ferredoxin 6 | Fdx6 | CCMP1779_3054-mRNA-1 |
| rieske [2Fe2S] ferredoxin 1 | rFDX1 | CCMP1779_7488-mRNA-1 |
| rieske [2Fe2S] ferredoxin 2 | rFDX2 | CCMP1779_4457-mRNA-1 |
| putative chloroplast ferredoxin 2 | Fdx2 | CCMP1779_2103-mRNA-1 |
| putative chloroplast ferredoxin 5 | Fdx5 | CCMP1779_7881-mRNA-1 |
| putative chloroplast ferredoxin 3 | Fdx3 | CCMP1779_1904-mRNA-1 |
| putative chloroplast ferredoxin 3 | Fdx3 | CCMP1779_1904-mRNA-1 |
|  | | |
| **IRON SULFUR CLUSTER BIOSYNTHESIS** | | |
| iron-sulfur cluster assembly protein | IscA 1 | CCMP1779_11149-mRNA-1 |
| iron-sulfur cluster assembly protein | IscA 2 | CCMP1779_6581-mRNA-1 |
| iron-sulfur cluster biosynthesis protein | Nar1 | CCMP1779_4004-mRNA-1 |
|  | | |
| **OXIDOREDUCTASES** | | |
| ferric reductase oxidase | Fro1 | CCMP1779_969-mRNA-1 |
|  | | |
| **ROS SCAVENGING** | | |
| glutathione synthase | GST | CCMP1779_1871-mRNA-1 |
| Microsomal glutathione S-transferase | mGST | CCMP1779_9028-mRNA-1 |
| superoxide dismutase | SOD | CCMP1779_6610-mRNA-1 |
| glutathione peroxidase | GPX | CCMP1779_10092-mRNA-1 |

^1^ this gene model is from augustus or snap gene annotation and was found superior to the final maker annotation after manual examination

^2^ transcript from EST data, no final gene model available
