# Supplementary material for: Genome, Functional Gene Annotation, and Nuclear Transformation of the Heterokont Oleaginous Alga Nannochloropsis oceanica CCMP1779
Source: PLoS Genet. 2012 Nov 15;8(11):e1003064. doi: 10.1371/journal.pgen.1003064 (PMC3499364; doi:10.1371/journal.pgen.1003064)
Supplement: Table S12 — Fatty acid composition of the major glycerolipids of Nannochloropsis CCMP1779. Averages are presented (n = 3) with standard deviation in parenthesis. (DOCX) [file pgen.1003064.s025.docx]

**Table S12:** Fatty acid composition of the major glycerolipids of Nannochloropsis CCMP1779. Averages are presented (n=3) with standard deviation in parenthesis.

|  | **MGDG** | | **DGDG** | | **SQDG** | | **DGTS** | | **PtdCho** | | **PtdEtn/**  **PtdGro** | | **TAG** | |
| --- | --- | --- | --- | --- | --- | --- | --- | --- | --- | --- | --- | --- | --- | --- |
| **14:0** | 13.0 | (0.79) | 6.7 | (0.31) | 5.2 | (0.28) | 4.8 | (0.8) | 1.4 | (0.23) | 1.5 | (0.12) | 7.4 | (0.7) |
| **16:0** | 14.7 | (0.67) | 35.8 | (2.12) | 50.1 | (0.47) | 18.8 | (0.99) | 22.0 | (1.39) | 41.6 | (0.51) | 45.9 | (3.11) |
| **16:1 ^1^** | 17.7 | (0.68) | 33.8 | (3.05) | 40.6 | (0.7) | 23.9 | (1.6) | 42.1 | (3.86) | 18.6 | (0.58) | 41.2 | (3.74) |
| **16:2** | 4.5 | (0.49) | 2.4 | (0.73) | 3.7 | (0.15) | 6.0 | (0.55) | 3.1 | (0.21) | 5.6 | (1.28) | 5.5 | (0.55) |
| **18:0** | 1.5 | (0.03) | n.d. |  | n.d. |  | 5.7 | (0.27) | 2.7 | (0.24) | 8.0 | (0.44) | n.d. |  |
| **18:1 ^2^** | 0.3 | (0.42) | 1.0 | (0.16) | n.d. |  | 0.4 | (0.52) | n.d. |  | 0.4 | (0.56) | n.d. |  |
| **18:2** | 1.0 | (0.2) | 1.8 | (0.38) | n.d. |  | 2.2 | (0.47) | 21.7 | (2.71) | 0.8 | (0.58) | n.d. |  |
| **20:4** | 1.4 | (0.02) | n.d. |  | n.d. |  | 5.3 | (0.25) | 2.5 | (0.22) | 7.4 | (0.41) | n.d. |  |
| **20:5** | 46.1 | (1.09) | 18.5 | (0.35) | 0.4 | (0.57) | 33.0 | (2.34) | 4.4 | (0.56) | 16.1 | (0.41) | n.d. |  |

^1^includes both 16:1Δ9 and 16:1Δ3*t*,

^2^includes both 18:1Δ9 and 18:1Δ11, n.d. not detectable
