# Supplementary material for: Genome, Functional Gene Annotation, and Nuclear Transformation of the Heterokont Oleaginous Alga Nannochloropsis oceanica CCMP1779
Source: PLoS Genet. 2012 Nov 15;8(11):e1003064. doi: 10.1371/journal.pgen.1003064 (PMC3499364; doi:10.1371/journal.pgen.1003064)
Supplement: Table S13 — Functional annotation of putative genes involved in fatty acid and glycerolipid biosynthesis. (DOCX) [file pgen.1003064.s026.docx]

**Table S13:** Functional annotation of putative genes involved in fatty acid and glycerolipid biosynthesis.

|  | **Description** | **Name** | **ID** |
| --- | --- | --- | --- |
| **Fatty Acid Synthesis** |  |  |  |
| PLASTID |  |  |  |
| **Acetyl-CoA carboxylase components** | Alpha-carboxyltransferase ^1,2^ | ACX1 | CCMP1779_4504-mRNA-1 |
|  |  |  | CCMP1779_7838-mRNA-1 |
|  | Beta-carboxyltransferase ^1,2^ | BCX | CCMP1779_2192-mRNA-1 |
|  | Biotin carboxylase | BCR1 | CCMP1779_1453-mRNA-1 |
|  |  | BCR2 | CCMP1779_5784-mRNA-1 |
|  | Biotin carboxyl carrier protein | BXP1 | CCMP1779_2363-mRNA-1 |
|  | Acyl carrier protein ^3^ | ACP1 | CCMP1779_9114-mRNA-1 |
|  | Malonyl-CoA : ACP Malonyltransferase | MCT | CCMP1779_6562-mRNA-1 |
| **Type II fatty acid synthase components** | 3-Ketoacyl-ACP synthase ^1^ | KAS1 | CCMP1779_3502-mRNA-1 |
|  | 3-Ketoacyl-ACP synthase ^1^ | KAS2 | No homolog |
|  | 3-Ketoacyl-ACP synthase III | KAS3 | CCMP1779_2094-mRNA-1 |
|  | 3-Ketoacyl-ACP reductase ^3^ | KAR1 | CCMP1779_512-mRNA-1 |
|  | 3-Hydroxyacyl-ACP dehydratase | HAD1 | CCMP1779_4800-mRNA-1 |
|  | Enoyl-ACP-reductase | ENR1 | CCMP1779_1026-mRNA-1 |
|  | Acyl-ACP thiolase | FAT | CCMP1779_2468-mRNA-1 |
| MITOCHONDRIA |  |  |  |
| **Type II fatty acid synthase components** | 3-Ketoacyl-ACP synthase ^4^ | KAS4 | CCMP1779_10584-mRNA-1 |
|  | 3-Ketoacyl-ACP reductase ^3^ | KAR2 | No homolog |
|  | 3-Hydroxyacyl-ACP dehydratase | HAD2 | CCMP1779_1544-mRNA-1 |
|  | Enoyl-ACP-reductase | ENR2 | CCMP1779_10010-mRNA-1 |
|  | Acyl carrier protein ^3^ | ACP2 | CCMP1779_9114-mRNA-1 |
| CYTOSOL |  |  |  |
|  | Acetyl-CoA carboxylase | ACC1 | CCMP1779_9552-mRNA-1 |
|  | Type I fatty acid synthase / PKS |  | CCMP1779_6720-mRNA-1 |
|  |  |  | CCMP1779_3502-mRNA-1 |
|  |  |  | CCMP1779_1983-mRNA-1 |
| **Glycerolipid Synthesis** |  |  |  |
| PLASTID PATHWAY |  |  |  |
|  | Glycerol-3-phosphate acyltransferase | GPAT1 | CCMP1779_4533-mRNA-1 |
|  | 1-sn-acyl-glycerol-3-phosphate acyltransferase | LPAT1 | CCMP1779_2512-mRNA-1 |
|  | Phosphatidate phosphatase | PAP | CCMP1779_4742-mRNA-1 |
|  | Phosphatidate cytidylstransferase | CDS1 | CCMP1779_6827-mRNA-1 |
|  | CPD-DAG-phosphotranferase |  | CCMP1779_3242-mRNA-1 |
|  | Phosphatidylglycerol synthase |  | CCMP1779_3242-mRNA-1 |
|  | UDP-sulfoquinovose synthase | SQD1 | augustus_masked-nanno_1008-abinit-gene-0.5-mRNA-1 ^4^ |
|  | Sulfolipid synthase | SQD2 | CCMP1779_4348-mRNA-1 |
|  | Monogalactosyl-1,2sn-diacylglycerol synthase | MGD1 | augustus_masked-nanno_3246-abinit-gene-0.1-mRNA-1 ^4^ |
|  | Digalactosyl-1,2,sn-diacyl-glycerol synthase | DGD1 | augustus_masked-nanno_564-abinit-gene-1.9-mRNA-1 ^4^ |
|  | Phosphatidylglycerol synthase |  | CCMP1779_3242-mRNA-1 |
| ER PATHWAY |  |  |  |
|  | Glycerol-3-phosphate acyltransferase, ER | GPAT2 | CCMP1779_11761-mRNA-1 |
|  | 1-sn-acyl-glycerol-3-phosphate acyltranserase like ^1^ |  | CCMP1779_10982-mRNA-1 |
|  |  |  | CCMP1779_1680-mRNA-1 |
|  |  |  | CCMP1779_10774-mRNA-1 |
|  |  |  | CCMP1779_11588-mRNA-1 |
|  |  |  | CCMP1779_7589-mRNA-1 |
|  |  |  | CCMP1779_8524-mRNA-1 |
|  | Lysocardiolipin acyltransferase, may be active on other lysophospholipids |  | CCMP1779_10166-mRNA-1 |
|  | Lipin like/ Phosphatidate phosphatase | LIPIN | CCMP1779_161-mRNA-1 |
|  | Cytidinediphosphate diacylglycerol synthase |  | augustus_masked-nanno_776-abinit-gene-0.6-mRNA-1 ^4^ |
|  | Betainlipid synthase | BTA1 | CCMP1779_10012-mRNA-1 |
|  | CDP-alcohol-DAG phosphatidyltransferase |  | CCMP1779_6627-mRNA-1 |
|  | CDP-alcohol-DAG phosphatidyltransferase |  | CCMP1779_665-mRNA-1 |
|  | Ethanolamine/choline kinase |  | CCMP1779_7806-mRNA-1 |
|  | Choline/ethanolamine kinase |  | augustus_masked-nanno_6752-abinit-gene-0.5-mRNA-1 ^4^ |
|  | CTP-phosphoethanolamine cytidyltransferase |  | CCMP1779_146-mRNA-1 |
|  | CTP-phosphocholine cytityltransferase |  | CCMP1779_7924-mRNA-1 |
|  | Inositol-3-phosphate synthase | INO1 | CCMP1779_10088-mRNA-1 |
|  | CDP-DAG inositol-3-phosphate phosphatidyltranferase | PIS1 | CCMP1779_7121-mRNA-1 |
| MITOCHONDRIA |  |  |  |
|  | Cardiolipin-Synthase | CLS | CCMP1779_245-mRNA-1 |
| **TAG Synthesis** |  |  |  |
|  | Diacylglycerol acyltransferase type2 | DGAT1 | CCMP1779_4340-mRNA-1 |
|  | Mono- or Diacylglycerol acyltransferase type2 | DGAT2 | CCMP1779_3705-mRNA-1 |
|  | Mono- or diacylglycerol acyltransferase type2 | DGAT3 | CCMP1779_7206-mRNA-1 |
|  | Mono- or diacylglycerol acyltransferase type2 | DGAT4 | CCMP1779_9929-mRNA-1 |
|  | Mono- or diacylglycerol acyltransferase type2 | DGAT5 | CCMP1779_3915-mRNA-1 |
|  | Mono- or diacylglycerol acyltransferase type2 | DGAT6 | CCMP1779_9590-mRNA-1 |
|  | Mono- or diacylglycerol acyltransferase type2 | DGAT7 | CCMP1779_3159-mRNA-1 |
|  | Mono- or diacylglycerol acyltransferase type2 | DGAT8 | CCMP1779_358-mRNA-1 |
|  | Mono- or diacylglycerol acyltransferase type2 | DGAT9 | CCMP1779_10272-mRNA-1 |
|  | Mono- or diacylglycerol acyltransferase type2 | DGAT10 | CCMP1779_3159-mRNA-1 |
|  | Mono- or diacylglycerol acyltransferase type2 | DGAT11 | CCMP1779_5368-mRNA-1 |
|  | Mono- or diacylglycerol acyltransferase type2 | DGAT12 | snap_masked-nanno_3592-abinit-gene-0.9-mRNA-1 ^4^ |
|  | Mono- or diacylglycerol acyltransferase type2 | DGAT13 | CCMP1779_5368-mRNA-1 |
|  | Phospholipid:DAG Acyltranferase | PDAT1 | CCMP1779_2212-mRNA-1 |
|  | Phospholipid:DAG Acyltranferase | PDAT2 | CCMP1779_8602-mRNA-1 |
|  | Diacylglycerol acyltransferase type1 |  | CCMP1779_3520-mRNA-1 |
| **Desaturases** |  |  |  |
|  | Acyl-ACP desaturase, plastid |  | CCMP1779_11506-mRNA-1 |
|  | Palmitoyl-Δ3*t*-desaturase | DES3*t* | CCMP1779_1787-mRNA-1 |
|  | Microsomal Δ9-desaturase | DES9 | CCMP1779_11542-mRNA-1 |
|  | Microsomal Δ12-desaturase | DES12 | CCMP1779_10636-mRNA-1 |
|  | Microsomal Δ6- desaturase | DES6 | CCMP1779_2179-mRNA-1 |
|  | Microsomal Δ5-desaturase | DES5 | CCMP1779_5794-mRNA-1 |
|  | Sterol desaturase |  | CCMP1779_8307-mRNA-1 |
|  | ω3-desaturase, partial sequence, N-terminal |  | CCMP1779_6416-mRNA-1 |
| **Elongases** |  |  |  |
|  | 18C-Δ9-elongase | ELO | CCMP1779_6306-mRNA-1 |
|  | Δ6-elongase |  | augustus_masked-nanno_776-abinit-gene-0.4-mRNA-1 ^4^ |
|  | Elongase |  | CCMP1779_11733-mRNA-1 |
|  | Elongase |  | CCMP1779_5130-mRNA-1 |
|  | Elongase |  | CCMP1779_1345-mRNA-1 |
|  | Elongase |  | CCMP1779_7311-mRNA-1 |
|  | Elongase |  | CCMP1779_10883-mRNA-1 |
|  | Elongase |  | CCMP1779_10919-mRNA-1 |
|  | Elongase |  | CCMP1779_1559-mRNA-1 |
|  | Elongase |  | CCMP1779_8604-mRNA-1 |
|  | Elongase |  | CCMP1779_7313-mRNA-1 |

^1^ isoforms cannot be unambiguously annotated

^2^ on plastid genome,

^3^ subcellular localization cannot be unambiguously predicted

^4^ this gene model is from augustus or snap gene annotation and was found superior to the final maker annotation after manual examination
