# Supplementary material for: Genome, Functional Gene Annotation, and Nuclear Transformation of the Heterokont Oleaginous Alga Nannochloropsis oceanica CCMP1779
Source: PLoS Genet. 2012 Nov 15;8(11):e1003064. doi: 10.1371/journal.pgen.1003064 (PMC3499364; doi:10.1371/journal.pgen.1003064)
Supplement: Table S14 — Genes predicted to encode enzymes putatively involved in fatty acid mobilization and degradation. (DOCX) [file pgen.1003064.s027.docx]

**Table S14**. Genes predicted to encode enzymes putatively involved in fatty acid mobilization and degradation.

| **Description** | **Name** | | ID |
| --- | --- | --- | --- |
| **β-Oxidation** | | | |
| **Long Chain Fatty Acyl CoA Synthetases** | | | |
| Acyl- synthetase long-chain family member | LACS1 | | CCMP1779_4001-mRNA-1 |
| AMP-dependent synthetase and ligase | LACS2 | | CCMP1779_3049-mRNA-1 |
| Acyl- synthetase (bubblegum family member 2) | LACS3 | | CCMP1779_4677-mRNA-1 |
| AMP-dependent synthetase and ligase | LACS4 | | CCMP1779_8795-mRNA-1 |
| Fatty-acid- ligase fadd9 | LACS5 | | CCMP1779_8752-mRNA-1 |
| AMP-dependent synthetase and ligase | LACS6 | | CCMP1779_10945-mRNA-1 |
| Acetyl-coenzyme a synthetase | LACS7 | | CCMP1779_956-mRNA-1 |
| AMP-dependent synthetase and ligase | LACS8 | | CCMP1779_1243-mRNA-1 |
| AMP-dependent synthetase and ligase | LACS9 | | CCMP1779_11454-mRNA-1 |
| **Mitochondrial acyl-CoA dehydrogenases** |  | |  |
| Acyl-CoA-dehydrogenase |  | | CCMP1779_6983-mRNA-1 |
| Acyl-CoA dehydrogenase |  | | CCMP1779_7415-mRNA-1 |
| Acyl-CoA-dehydrogenase |  | | CCMP1779_9617-mRNA-1 |
| **Peroxisomal acyl-CoA-oxidases** |  | |  |
| Acyl-CoA-oxidase |  | | CCMP1779_10648-mRNA-1 |
| **Mitochondrial trifunctional enzyme** |  | |  |
| Enoyl-CoA hydratase/3-hydroxyacyl-CoA dehydrogenase alpha-subunit of trifunctional enzyme |  | | CCMP1779_4030-mRNA-1 |
| Acetyl-CoA acyltransferase |  | | CCMP17792420-mRNA-1 |
| **Peroxisomal multifunctional enzymes** |  | |  |
| Enoyl-CoA hydratase and Δ3-cis- Δ2-trans-enoyl-CoA isomerase |  | | CCMP1779_6411-mRNA-1 |
| **Thiolases (localization uncertain)** |  | |  |
| Acetyl-CoA acyltransferase |  | | CCMP1779_11662-mRNA-1 |
| Acetyl-CoA acyltransferase |  | | CCMP1779_10515-mRNA-1 |
| Acetyl-CoA acyltransferase |  | | CCMP1779_3591-mRNA-1 |
| **Auxiliary enzymes for unsaturated fatty acid oxidation (localization uncertain)** |  | |  |
| Δ(3,5)- Δ (2,4)-dienoyl-CoA isomerase |  | | CCMP1779_5886-mRNA-1 |
| Enoyl-CoAhydratase/isomerase- |  | | CCMP1779_9049-mRNA-1 |
| Enoyl-CoA hydratase |  | | CCMP1779_657-mRNA-1 |
| Enoyl-CoA hydratase |  | | CCMP1779_776-mRNA-1 |
| Dehydratase/epimerase |  | | CCMP1779_8830-mRNA-1 |
| 2,4-dienoyl- reductase |  | | CCMP1779_10142-mRNA-1 |
| 2,4-dienoyl- reductase |  | | CCMP1779_5594-mRNA-1 |
| 2,4-dienoyl- reductase |  | | CCMP1779_8840-mRNA-1 |
| **Lipases** | | | |
| Lysophospholipase II, hydrolase |  | CCMP1779_8605-mRNA-1 | |
| Lysophospholipase-like 1 F, hydrolase |  | CCMP1779_5357-mRNA-1 | |
| Similar to Cr Phospholipase A3 |  | CCMP1779_7920-mRNA-1 | |
| Patatin-like domain-containing protein |  | CCMP1779_4454-mRNA-1 | |
| Patatin-like |  | CCMP1779_11320-mRNA-1 | |
| Patatin-like phospholipase, metabolic process, triglyceride lipase |  | CCMP1779_10681-mRNA-1 | |
| similar to sn1-specific diacylglycerol lipase alpha, triglyceride lipase |  | CCMP1779_5711-mRNA-1 | |
| Triacylglycerol lipase, protein binding |  | CCMP1779_10796-mRNA-1 | |
| Triglyceride lipase |  | CCMP1779_853-mRNA-1 | |
| Triglyceride lipase |  | CCMP1779_5911-mRNA-1 | |
| Triglyceride lipase |  | CCMP1779_8929-mRNA-1 | |
| Lipase class 3 family protein, triglyceride lipase |  | CCMP1779_1271-mRNA-1 | |
| Lipase family protein, triglyceride Lipase |  | CCMP1779_733-mRNA-1 | |
| Lipase, active site, triglyceride lipase |  | CCMP1779_10426-mRNA-1 | |
| similar to Cr Alpha/beta hydrolase fold, Esterase/lipase/thioesterase |  | CCMP1779_10868-mRNA-1 | |
| similar to Cr Alpha/beta hydrolase fold, Esterase/lipase/thioesterase |  | CCMP1779_938-mRNA-1 | |
| similar to Cr Alpha/beta hydrolase fold, Esterase/lipase/thioesterase |  | CCMP1779_5940-mRNA-1 | |
| alpha beta fold family protein, hydrolase |  | CCMP1779_373-mRNA-1 | |
| alpha beta fold family protein, hydrolase |  | CCMP1779_422-mRNA-1 | |
| similar to Cr Esterase/lipase/thioesterase |  | CCMP1779_11505-mRNA-1 | |
| similar to Cr Esterase/lipase/thioesterase |  | CCMP1779_11217-mRNA-1 | |
| similar to Cr Esterase/lipase/thioesterase |  | CCMP1779_10684-mRNA-1 | |
| similar to Cr Esterase/lipase/thioesterase |  | CCMP1779_6531-mRNA-1 | |
| similar to Cr Ubiquitin interacting motif, Esterase/lipase/thioesterase |  | CCMP1779_4982-mRNA-1 | |
| similar to Cr Ubiquitin interacting motif, Esterase/lipase/thioesterase |  | CCMP1779_4871-mRNA-1 | |
| Putative lipase |  | CCMP1779_7009-mRNA-1 | |
| Putative lipase |  | CCMP1779_1337-mRNA-1 | |
| Putative lipase |  | CCMP1779_5684-mRNA-1 | |
| Putative lipase |  | CCMP1779_4309-mRNA-1 | |
| Putative lipase |  | CCMP1779_10823-mRNA-1 | |
| Putative lipase |  | CCMP1779_11068-mRNA-1 | |
| Putative lipase |  | CCMP1779_4494-mRNA-1 | |
| Putative lipase |  | CCMP1779_2197-mRNA-1 | |
| Putative lipase |  | CCMP1779_7700-mRNA-1 | |
| Putative lipase |  | CCMP1779_6907-mRNA-1 | |
| Putative lipase |  | CCMP1779_10607-mRNA-1 | |
| Putative lipase |  | CCMP1779_7890-mRNA-1 | |
| Putative lipase |  | CCMP1779_7890-mRNA-1 | |
| Putative lipase |  | CCMP1779_2504-mRNA-1 | |
| Putative lipase |  | CCMP1779_10798-mRNA-1 | |
| Putative lipase |  | CCMP1779_10149-mRNA-1 | |
| Putative lipase |  | CCMP1779_9696-mRNA-1 | |
| Putative lipase |  | CCMP1779_7353-mRNA-1 | |
| Putative lipase |  | CCMP1779_8379-mRNA-1 | |
| Putative lipase |  | CCMP1779_9039-mRNA-1 | |
| Putative lipase |  | CCMP1779_7705-mRNA-1 | |
| Putative lipase |  | CCMP1779_10811-mRNA-1 | |
| Putative lipase |  | CCMP1779_3390-mRNA-1 | |
| Putative lipase |  | CCMP1779_10339-mRNA-1 | |
| Putative lipase |  | CCMP1779_5488-mRNA-1 | |
| Putative lipase |  | CCMP1779_3546-mRNA-1 | |
