# Supplementary material for: Genome, Functional Gene Annotation, and Nuclear Transformation of the Heterokont Oleaginous Alga Nannochloropsis oceanica CCMP1779
Source: PLoS Genet. 2012 Nov 15;8(11):e1003064. doi: 10.1371/journal.pgen.1003064 (PMC3499364; doi:10.1371/journal.pgen.1003064)
Supplement: Table S15 — Genes predicted to encode enzymes putatively involved in cell wall metabolism. (DOCX) [file pgen.1003064.s028.docx]

**Table S15**. Genes predicted to encode enzymes putatively involved in cell wall metabolism

| **Description** | **CAZy Family** | **ID** |
| --- | --- | --- |
| Cellulose synthase | GT2 | CCMP1779_5780-mRNA-1 |
| Cellulose synthase | GT2 | CCMP1779_7935-mRNA-1 |
| Endoglucanase | GH9 | CCMP1779_8534-mRNA-1 |
| Endoglucanase | GH9 | CCMP1779_8535-mRNA-1 |
| Endoglucanase | GH9 | CCMP1779_8356-mRNA-1 |
| Endoglucanase | GH9 | CCMP1779_11399-mRNA-1 |
| Endoglucanase | GH9 | CCMP1779_163-mRNA-1 |
| Endoglucanase | GH9 | CCMP1779_5548-mRNA-1 |
