# Supplementary material for: Genome, Functional Gene Annotation, and Nuclear Transformation of the Heterokont Oleaginous Alga Nannochloropsis oceanica CCMP1779
Source: PLoS Genet. 2012 Nov 15;8(11):e1003064. doi: 10.1371/journal.pgen.1003064 (PMC3499364; doi:10.1371/journal.pgen.1003064)
Supplement: Table S16 — Predicted genes in the biosynthetic pathways of Asp-derived, aromatic and branched-chain amino acids and in nitrogen assimilation in CCMP1779. (DOCX) [file pgen.1003064.s029.docx]

**Table S16**. Predicted genes in the biosynthetic pathways of Asp-derived, aromatic and branched-chain amino acids and in nitrogen assimilation in CCMP1779

| **Description** | **Name** | **ID** |
| --- | --- | --- |
| **Biosynthesis of Asp-derived amino acids** |  |  |
| Asp kinase | AK | CCMP1779_3150 |
| Asp kinase - homoserine dehydrogenase | AK-HSDH^1^ | CCMP1779_10446 |
| Homoserine dehydrogenase | HSDH | augustus_masked-nanno_5085-abinit-gene-0.2 ^5^ |
| Asp semialdehyde dehydrogenase | ASD | CCMP1779_11914 |
| Asp semialdehyde dehydrogenase | ASD | CCMP1779_6330 |
| Dihydrodipicolinate synthase | DHDPS | CCMP1779_11357 |
| Dihydrodipicolinate reductase | DHDPR^2^ | CCMP1779_4610 |
| Diaminopimelate aminotransferase | DAPAT | augustus_masked-nanno_2338-abinit-gene-0.3 ^5^ |
| Diaminopimelate epimerase | DAPE^2^ | CCMP1779_10579 |
| Diaminopimelate decarboxylase | DAPDC | CCMP1779_2505 |
| Diaminopimelate decarboxylase | DAPDC | CCMP1779_8817 |
| Homoserine kinase | HSK | CCMP1779_8751 |
| Cystathionine γ synthase | CGS | CCMP1779_10900 |
| Cystathionine γ synthase | CGS | CCMP1779_4094 |
| Cystathionine β lyase | CBL | CCMP1779_9018 |
| Cystathionine β lyase | CBL | CCMP1779_9158 |
| Cystathionine β lyase | CBL | CCMP1779_3085 |
| Homocysteine S-methyltransferase | HMT | CCMP1779_1690 |
| Cobalamin-dependent Met synthase | MetH | CCMP1779_2985 |
| Cobalamin-independent Met synthase | MS | CCMP1779_5185 |
| Cobalamin-independent Met synthase | MS | CCMP1779_8229 |
| Thr synthase | TS^3^ | CCMP1779_10068 |
| Thr synthase | TS^2^ | CCMP1779_4990 |
| **Biosynthesis of aromatic amino acids** |  |  |
| 3-Deoxy-D-arabino-heptulosonate-7-phosphate synthase | DAHPS | CCMP1779_7898 |
| Dehydroquinate synthase | DHQS | augustus_masked-nanno_505-abinit-gene-0.2-mRNA-1 ^5^ |
| Dehydroquinate dehydratase - shikimate dehydrogenase | DHQDH-SDH^1^ | CCMP1779_5703 |
| Shikimate kinase | SK | CCMP1779_10591 |
| 5-Enolpyruvylshikimate-3-phosphate synthase | EPSPS | CCMP1779_6038 |
| Chorismate synthase | CS^3^ | CCMP1779_8941 |
| Chorismate synthase | CS^3^ | CCMP1779_2899 |
| Chorismate mutase | CM | CCMP1779_234 |
| Arogenate dehydratase - arogenate dehydrogenase | ADT-ADH^1^ | CCMP1779_11837 |
| Anthranilate synthase α and β subunits | ASA-ASB | CCMP1779_4984 |
| Anthranilate phosphoribosyltransferase | PRT | CCMP1779_4197 |
| Indole-3-glycerol phosphate synthase - phosphoribosylanthranilate isomerase | IGPS-PAI^1^ | CCMP1779_5546 |
| Indole-3-glycerol phosphate synthase | IGPS^4^ | CCMP1779_398 |
| Trp synthase α and β subunits | TSA-TSB^1^ | CCMP1779_102 |
| **Biosynthesis of branched-chain amino acids** |  |  |
| Thr deaminase | TD | CCMP1779_7883 |
| Acetohydroxyacid synthase | AHAS | CCMP1779_3427 |
| Ketolacid reductoisomerase | KARI | CCMP1779_6007 |
| Dihydroxyacid dehydratase | DHAD | CCMP1779_4633 |
| Dihydroxyacid dehydratase | DHAD^3^ | CCMP1779_7539 |
| Isopropylmalate synthase | IPMS^3^ | CCMP1779_4567 |
| Isopropylmalate isomerase large subunit | IPMIL | CCMP1779_2773 |
| Isopropylmalate isomerase small subunit | IPMIS^3^ | CCMP1779_5262 |
| Isopropylmalate dehydrogenase | IMD | CCMP1779_7869 |
| Branched-chain aminotransferase | BCAT | CCMP1779_8799 |
| Branched-chain aminotransferase | BCAT^3^ | CCMP1779_5665 |
| Branched-chain aminotransferase | BCAT^2^ | CCMP1779_10881 |
| **Nitrogen Assimilation** |  |  |
| High affinity nitrate transporter | NRT2:1 | CCMP1779_438 |
| Nitrite transporter | NAR1 | CCMP1779_6536 |
| Ammonium transporter | AMT1:1 | CCMP1779_5503 |
| NAD(P)H:Nitrite reductase | NIR | CCMP1779_6877 |
| Ferredoxin nitrite reductase | NII | CCMP1779_9262 |
| NAD(P)H-Nitrate reductase | NIA | CCMP1779_443 |

Genes in the biosynthetic pathways of Asp-derived, aromatic and branched-chain amino acids were manually annotated. For each *N. oceanica* locus, the gene function was predicted based on BLAST similarity to homologous genes in Arabidopsis. The accuracy of gene function prediction was evaluated by PFAM domain support and reverse blast searches of Arabidopsis homologs with *N. oceanica* genes.

^1^These genes are predicted to be fused with other genes in the same pathway in *N. oceanica*.

^2^These predicted genes are missing the start codon.

^3^These predicted genes are missing the stop codon.

^4^This IPGS gene appears to be fused with some unrelated gene(s).

^5^this gene model is from augustus or snap gene annotation and was found superior to the final maker annotation after manual examination.
