# Supplementary material for: Genome, Functional Gene Annotation, and Nuclear Transformation of the Heterokont Oleaginous Alga Nannochloropsis oceanica CCMP1779
Source: PLoS Genet. 2012 Nov 15;8(11):e1003064. doi: 10.1371/journal.pgen.1003064 (PMC3499364; doi:10.1371/journal.pgen.1003064)
Supplement: Table S17 — Presence of fused genes in essential amino acid biosynthesis in representative bacteria, cyanobacteria, green algae, diatoms, Nannochloropsis and higher plants. (DOCX) [file pgen.1003064.s030.docx]

**Table S17.** Presence of fused genes in essential amino acid biosynthesis in representative bacteria, cyanobacteria, green algae, diatoms, Nannochloropsis and higher plants

| Gene pairs | *E. coli* | Synechocystis *sp.* | *C. reinhardtii* | *P. tricornutum* | *T. pseudonana* | *N. oceanica* | Arabidopsis |
| --- | --- | --- | --- | --- | --- | --- | --- |
| *AK-HSDH* | + | - | + | + | + | + | + |
| *DHQDH-SDH* | - ^1^ | - | + | + | + | + | + |
| *ADT-ADH* | - | - | - | + | + | + | - |
| *ASA-ASB* | - ^2^ | - ^3^ | + | + | + | + | + |
| *IGPS-PAI* | + | - | - | + | + | + | - |
| *TSA-TSB* | - | - | - | + | + | + | - |

^1^Although fused *DHQDH-SDH* was not found in *E. coli*, it is present in other bacteria, such as *Acidobacterium sp.* and *Planctomyces maris.*

^2^Although fused *ASA-ASB* was not found in *E. coli*, it is present in other bacteria, such *Legionella longbeachae* and *Sorangium cellulosum*.

^3^Although fused *ASA-ASB* was not found in Synechocystis *sp.*, it is present in other cyanobacteria, such as *Anabaena variabilis* and *Nostoc sp*.*.*

The accession numbers for AK-HSDH homologs in *E. coli*, *C. reinhardtii*, *P. tricornutum*, *T. pseudonana*, and Arabidopsis are ZP_08372323, XP_001695256, XP_002182284, XP_002296299, At1g31230, and At4g19710. The accession numbers for DHQDH-SDH homologs in *Acidobacterium sp.*, *P. maris*, *C. reinhardtii*, *P. tricornutum*, *T. pseudonana*, and Arabidopsis are ZP_07032873, ZP_01854559, XP_001694346, XP_002179655, XP_002289031, and At3g06350. ADT-ADH homologs in *P. tricornutum* and *T. pseudonana* were obtained using tBLASTn at [www.jgi.doe.gov](http://www.jgi.doe.gov); the corresponding accession numbers are estExt_fgenesh1_pg.C_chr_130260 and estExt_fgenesh1_pg.C_chr_10420. The accession numbers for ASA-ASB homologs in *L. longbeachae*, *S. cellulosum*, *A. variabilis*, *Nostoc sp.* *C. reinhardtii*, *P. tricornutum*, *T. pseudonana*, and Arabidopsis are YP_003455732, YP_001615687, YP_325382, NP_484458, XP_001702943, XP_002177062, XP_002287912, and At2g28880. The accession number for IGPS-PAI homolog in *E. coli* is ZP_06938008. IGPS-PAI homologs in *P. tricornutum* and *T. pseudonana* were obtained using tBLASTn at [www.jgi.doe.gov](http://www.jgi.doe.gov); the corresponding accession numbers are estExt_fgenesh1_pg.C_chr_60121 (from DOE-JGI) and fgenesh1_pg.C_chr_10000275. The accession numbers for TSA-TSB homologs in *P. tricornutum* and *T. pseudonana* are XP_002176877 and XP_002294706.
