# Supplementary material for: Genome, Functional Gene Annotation, and Nuclear Transformation of the Heterokont Oleaginous Alga Nannochloropsis oceanica CCMP1779
Source: PLoS Genet. 2012 Nov 15;8(11):e1003064. doi: 10.1371/journal.pgen.1003064 (PMC3499364; doi:10.1371/journal.pgen.1003064)
Supplement: Table S18 — Putative Nannochloropsis genes involved in sulfate assimilation and metabolism. (DOCX) [file pgen.1003064.s031.docx]

**Table S18.** Putative Nannochloropsis genes involved in sulfate assimilation and metabolism

| **Description** | **NAME** | **ID** |
| --- | --- | --- |
| **Sulfate assimilation and metabolism** |  |  |
| ATP sulfurylase | ATPS | CCMP1779_3861-mRNA-1 |
| PAPS synthetase | APK | CCMP1779_7885-mRNA-1 |
| Type-B APS reductase | APR | CCMP1779_4709-mRNA-1 |
| Sulfite reductase | SIR | CCMP1779_1994-mRNA-1 |
| Sulfite reductase | SIR | CCMP1779_9362-mRNA-1 |
| Sulfite reductase | SIR | CCMP1779_9697-mRNA-1 |
| Serine acetyltransferase | SAT ^1^ | CCMP1779_1186-mRNA-1 |
| Cysteine synthase | CS | CCMP1779_10055-mRNA-1 |
| Cystathionine γ -synthase | CGS | CCMP1779_4094-mRNA-1 |
| Cystathionine β -lyase | CBL | CCMP1779_9158-mRNA-1 |
| Cobalamin-dependent methionine synthase | MS | CCMP1779_2985-mRNA-1 |
| Cobalamin-independent methionine synthase | MS | CCMP1779_5185-mRNA-1 |
| S-adenosylmethionine synthetase | SAM | CCMP1779_9387-mRNA-1 |
| Homoserine acetyltransferase | HSAT | CCMP1779_8402-mRNA-1 |
| Homocysteine synthase | HCS | CCMP1779_10900-mRNA-1 |
| Cystathionine β-synthase | CBS | CCMP1779_3275-mRNA-1 |
| Cystathionine γ-lyase | CGL | CCMP1779_9018-mRNA-1 |
| γ-glutamylcysteine synthetase | γ-ECS ^2^ | CCMP1779_10993-mRNA-1 |
| Glutathione synthetase | GSHS | CCMP1779_1871-mRNA-1 |
| Glutathione synthetase | GSHS | CCMP1779_6638-mRNA-1 |
| Phytochelatin synthetase | PCS | CCMP1779_214-mRNA-1 |
| Phytochelatin synthetase | PCS | CCMP1779_8531-mRNA-1 |
| Sulfate transporter | SULTR | CCMP1779_6884-mRNA-1 |
| Sulfate transporter | SULTR | CCMP1779_4464-mRNA-1 |
| Sulfate transporter | SULTR | CCMP1779_2237-mRNA-1 |
| Sulfate transporter | SULTR | CCMP1779_610-mRNA-2 |
| Sulfate transporter | SULTR | CCMP1779_2945-mRNA-1 |
| Sulfate transporter | SULTR | CCMP1779_430-mRNA-1 |
| Sulfate transporter | SULTR | CCMP1779_4159-mRNA-1 |
| Sulfate transporter | SULTR | CCMP1779_3645-mRNA-1 |
| Sulfate transporter | SULTR | CCMP1779_6348-mRNA-1 |
| diphosphonucleoside phosphatase (bisphosphate nucleotidase) | DPNP | CCMP1779_2677-mRNA-1 |
| PAPS transporter | PAPST | CCMP1779_6951-mRNA-1 |
| PAPS transporter | PAPST | CCMP1779_11941-mRNA-1 |
| Aryl/alkyl sulfatase | ARS | CCMP1779_11904-mRNA-1 |
| S-adenosylhomocysteine hydrolase | SAHH ^3^ | CCMP1779_11453-mRNA-1 |
| gamma-glutamyltranspeptidase | GGT | CCMP1779_2736-mRNA-1 |
| glutathione S-transferase | GST | CCMP1779_10820-mRNA-1 |
| glutathione S-transferase | GST | CCMP1779_7124-mRNA-1 |
| glutathione S-transferase | GST | CCMP1779_4979-mRNA-1 |
| glutathione S-transferase | GST | CCMP1779_9177-mRNA-1 |
| glutathione S-transferase | GST | CCMP1779_2705-mRNA-1 |
| glutathione S-transferase | GST | CCMP1779_7676-mRNA-1 |
| glutathione S-transferase | GST | CCMP1779_1827-mRNA-1 |
| glutathione S-transferase | GST | CCMP1779_2416-mRNA-1 |
| glutathione S-transferase | GST | CCMP1779_6045-mRNA-1 |

^1^ C-terminus has an unusual long extension

^2^ this gene model encodes the N-terminal part of the protein

^3^ fused to aspartate aminotransferase
