# Supplementary material for: Genome, Functional Gene Annotation, and Nuclear Transformation of the Heterokont Oleaginous Alga Nannochloropsis oceanica CCMP1779
Source: PLoS Genet. 2012 Nov 15;8(11):e1003064. doi: 10.1371/journal.pgen.1003064 (PMC3499364; doi:10.1371/journal.pgen.1003064)
Supplement: Table S19 — Putative chloroplast protein import related genes identified in the CCMP1779 genomic sequence. (DOCX) [file pgen.1003064.s032.docx]

**Table S19.** Putative chloroplast protein import related genes identified in the CCMP1779 genomic sequence.

| **Description** | **NAME** | **ID** |
| --- | --- | --- |
| **Chloroplast protein import related genes** |  |  |
| Translocon at the inner-envelope-membrane of chloroplasts, 110 kDa | Tic110 | CCMP1779_1420-mRNA-1 |
| Translocon at the inner-envelope-membrane of chloroplasts, 20 kDa | Tic20 | CCMP1779_10508-mRNA-1 |
| Translocon at the inner-envelope-membrane of chloroplasts, 22 kDa | Tic22 | CCMP1779_5216-mRNA-1 |
| Translocon at the inner-envelope-membrane of chloroplasts, 62 kDa | Tic62 | CCMP1779_10312-mRNA-1 |
| Stromal processing peptidase | SPP | CCMP1779_5777-mRNA-1 |
| **Non-chloroplastic genes of similar families** |  |  |
| Heat shock protein 100 family gene | Hsp100 | CCMP1779_2758-mRNA-1 |
| Heat shock protein 100 family gene | Hsp100 | CCMP1779_7349-mRNA-1 |
| Heat shock protein 100 family gene | Hsp100 | CCMP1779_7491-mRNA-1 |
| Heat shock protein 70 family gene-mitochondrial type | Hsp70 | CCMP1779_8242-mRNA-1 |
| Heat shock protein 70 family gene-lumenal type | Hsp70 | CCMP1779_5150-mRNA-1 |
| Type I signal peptidase family gene | SPaseI | CCMP1779_1061-mRNA-1 |
