# Supplementary material for: Genome, Functional Gene Annotation, and Nuclear Transformation of the Heterokont Oleaginous Alga Nannochloropsis oceanica CCMP1779
Source: PLoS Genet. 2012 Nov 15;8(11):e1003064. doi: 10.1371/journal.pgen.1003064 (PMC3499364; doi:10.1371/journal.pgen.1003064)
Supplement: Table S20 — Summary of testing the HECTAR heterokont protein localization prediction tool. Detailed information on the tested sequences and results is available in Table S25. (DOCX) [file pgen.1003064.s033.docx]

**Table S20.** Summary of testing the HECTAR heterokont protein localization prediction tool. Detailed information on the tested sequences and results is available in Table S25.

|  |  |  |  | **Prediction** | | | | |
| --- | --- | --- | --- | --- | --- | --- | --- | --- |
| **Test Set** | # of sequences | % of correct prediction | % of false positives total | Chloro-plast | Mito-chondria | Type II Signal Anchor | Signal Peptide | no Signal Peptide |
| Chloroplast | 44 | 43 | 0 | 19 | 0 | 2 | 10 | 13 |
| Mitochondria | 25 | 52 | 0 | 0 | 13 | 0 | 3 | 9 |
| Secretory | 20 | 15 | 14 | 0 | 0 | 1 | 2 | 17 |
| Nucleus/ no SP | 18 | 100 | 36 | 0 | 0 | 0 | 0 | 18 |
