# Supplementary material for: Genome, Functional Gene Annotation, and Nuclear Transformation of the Heterokont Oleaginous Alga Nannochloropsis oceanica CCMP1779
Source: PLoS Genet. 2012 Nov 15;8(11):e1003064. doi: 10.1371/journal.pgen.1003064 (PMC3499364; doi:10.1371/journal.pgen.1003064)
Supplement: Table S21 — Predicted genes involved in organelle division. (DOCX) [file pgen.1003064.s034.docx]

**Table S21.** Predicted genes involved in organelle division

| **Description** | **NAME** | **ID** |
| --- | --- | --- |
| Tubulin-like GTPase | FtsZ ^1^ | augustus_masked-nanno_396-abinit-gene-1.13-mRNA-1^4^ |
| Tubulin-like GTPase | FtsZ ^1^ | augustus_masked-nanno_312-abinit-gene-0.12-mRNA-1^4^ |
| Tubulin-like GTPase | FtsZ ^2^ | augustus_masked-nanno_1064-abinit-gene-0.6-mRNA-1^4^ |
| Dynamin-like protein | ARC5/DRP5B ^3^ | CCMP1779_7886-mRNA-1 |
| Septum site-determining protein MinD | MinD ^2^ | CCMP1779_5101-mRNA-1 |
| Septum site-determining protein MinC | MinC ^2^ | CCMP1779_9799-mRNA-1 |

^1^ predicted to be localized to the chloroplast

^2^ predicted to be localized to the mitochondria

^3^ predicted to be localized to the cytosol

^4^ this gene model is from augustus or snap gene annotation and was found superior to the final maker annotation after manual examination
