# Supplementary material for: Genome, Functional Gene Annotation, and Nuclear Transformation of the Heterokont Oleaginous Alga Nannochloropsis oceanica CCMP1779
Source: PLoS Genet. 2012 Nov 15;8(11):e1003064. doi: 10.1371/journal.pgen.1003064 (PMC3499364; doi:10.1371/journal.pgen.1003064)
Supplement: Table S22 — Genes predicted to be involved in light signaling. (DOCX) [file pgen.1003064.s035.docx]

**Table S22.** Genes predicted to be involved in light signaling

| **Description** | **NAME** | **ID** |
| --- | --- | --- |
| Cryptochrome/photolyase family protein | NoCPF1 | CCMP1779_4809-mRNA-1 |
| Cryptochrome/photolyase family protein | NoCPF2 | augustus_masked-nanno_1311-abinit-gene-0.9-mRNA-1 ^1^ |
| Aureochrome like protein | NoAUREO2 | CCMP1779_10112-mRNA-1 |
| Aureochrome like protein | NoAUREO3 | CCMP1779_10447-mRNA-1 |
| Aureochrome like protein | NoAUREO4 | CCMP1779_5385-mRNA-1 |

^1^ this gene model is from augustus or snap gene annotation and was found superior to the final maker annotation after manual examination
