# Supplementary material for: Genome, Functional Gene Annotation, and Nuclear Transformation of the Heterokont Oleaginous Alga Nannochloropsis oceanica CCMP1779
Source: PLoS Genet. 2012 Nov 15;8(11):e1003064. doi: 10.1371/journal.pgen.1003064 (PMC3499364; doi:10.1371/journal.pgen.1003064)
Supplement: Table S23 — Putative transcription factors and transcriptional regulators. (DOCX) [file pgen.1003064.s036.docx]

**Table S23**. Putative transcription factors and transcriptional regulators

| **Family** | **Domain** | **PF#** | | **ID** |
| --- | --- | --- | --- | --- |
| **TRANSCRIPTION FACTORS** | | | | |
| **AP2-EREBP (6)** | AP2 | PF00847.13 | CCMP1779_10810-mRNA-1 | |
|  | AP2 | PF00847.13 | CCMP1779_7582-mRNA-1 | |
|  | AP2 | PF00847.13 | CCMP1779_11218-mRNA-1 | |
|  | AP2 | PF00847.13 | CCMP1779_5575-mRNA-1 | |
|  | AP2 | PF00847.13 | CCMP1779_6908-mRNA-1 | |
|  | AP2 | PF00847.13 | CCMP1779_4174-mRNA-1 | |
| **bHLH (6)** | HLH | PF00010.19 | CCMP1779_1117-mRNA-1 | |
|  | HLH | PF00010.19 | CCMP1779_841-mRNA-1 | |
|  | HLH | PF00010.19 | CCMP1779_1117-mRNA-1 | |
|  | HLH | PF00010.19 | CCMP1779_5887-mRNA-1 | |
|  | HLH | PF00010.19 | CCMP1779_9983-mRNA-1 | |
|  | HLH | PF00010.19 | CCMP1779_1600-mRNA-1 | |
| **BSD (1)** | BSD | PF03909.10 | CCMP1779_3614-mRNA-1 | |
| **bZIP (8)** | bZIP_1 & bZIP_2 | PF00170.14 | snap_masked-nanno_1035-abinit-gene-0.56-mRNA-1^1^ | |
|  | bZIP_1 & bZIP_2 | PF00170.14 | CCMP1779_10112-mRNA-1 | |
|  | bZIP_1 & bZIP_2 | PF00170.14 | CCMP1779_10986-mRNA-1 | |
|  | bZIP_1 & bZIP_2 | PF00170.14 | CCMP1779_6318-mRNA-1 | |
|  | bZIP_1 & bZIP_2 | PF00170.14 | CCMP1779_10268-mRNA-1 | |
|  | bZIP_1 & bZIP_2 | PF00170.14 | CCMP1779_10512-mRNA-1 | |
|  | bZIP_1 & bZIP_2 | PF00170.14 | CCMP1779_10447-mRNA-1 | |
|  | bZIP_2 | PF07716.8 | CCMP1779_5385-mRNA-1 | |
| **C2H2 (1)** | zf-C2H2 | PF00096.19 | CCMP1779_4418-mRNA-1 | |
| **C3H (4)** | zf-CCCH | PF00642.17 | CCMP1779_9066-mRNA-1 | |
|  | zf-CCCH | PF00642.17 | CCMP1779_3417-mRNA-1 | |
|  | zf-CCCH | PF00642.17 | CCMP1779_11131-mRNA-1 | |
|  | zf-CCCH | PF00642.17 | CCMP1779_2132-mRNA-1 | |
| **CCAAT (10)** | CBFB_NFYA | PF02045.8 | CCMP1779_4505-mRNA-1 | |
|  | CBFD_NFYB_HMF | PF00808.16 | CCMP1779_10692-mRNA-1 | |
|  | CBFD_NFYB_HMF | PF00808.16 | CCMP1779_10337-mRNA-1 | |
|  | CBFD_NFYB_HMF | PF00808.16 | CCMP1779_11634-mRNA-1 | |
|  | CBFD_NFYB_HMF | PF00808.16 | CCMP1779_3419-mRNA-1 | |
|  | CBFD_NFYB_HMF | PF00808.16 | CCMP1779_1612-mRNA-1 | |
|  | CBFD_NFYB_HMF | PF00808.16 | CCMP1779_7528-mRNA-1 | |
|  | CBFD_NFYB_HMF | PF00808.16 | CCMP1779_10337-mRNA-1 | |
|  | CBFD_NFYB_HMF | PF00808.16 | CCMP1779_5926-mRNA-1 | |
|  | CBFD_NFYB_HMF | PF00808.16 | CCMP1779_197-mRNA-1 | |
| **CPP (4)** | CXC | PF03638.8 | CCMP1779_7252-mRNA-1 | |
|  | CXC | PF03638.8 | CCMP1779_7741-mRNA-1 | |
|  | CXC | PF03638.8 | CCMP1779_10961-mRNA-1 | |
|  | CXC | PF03638.8 | CCMP1779_1125-mRNA-1 | |
| **E2F-DP (3)** | E2F_TDP | PF02319.13 | CCMP1779_11267-mRNA-1 | |
|  | E2F_TDP | PF02319.13 | CCMP1779_11267-mRNA-1 | |
|  | E2F_TDP | PF02319.13 | CCMP1779_3316-mRNA-1 | |
| **FHA (12)** | FHA | PF00498.19 | CCMP1779_10364-mRNA-1 | |
|  | FHA | PF00498.19 | CCMP1779_5309-mRNA-1 | |
|  | FHA | PF00498.19 | CCMP1779_5580-mRNA-1 | |
|  | FHA | PF00498.19 | CCMP1779_1474-mRNA-1 | |
|  | FHA | PF00498.19 | CCMP1779_4898-mRNA-1 | |
|  | FHA | PF00498.19 | CCMP1779_2684-mRNA-1 | |
|  | FHA | PF00498.19 | CCMP1779_4063-mRNA-1 | |
|  | FHA | PF00498.19 | CCMP1779_8961-mRNA-1 | |
|  | FHA | PF00498.19 | CCMP1779_5552-mRNA-1 | |
|  | FHA | PF00498.19 | CCMP1779_927-mRNA-1 | |
|  | FHA | PF00498.19 | CCMP1779_49-mRNA-1 | |
|  | FHA | PF00498.19 | CCMP1779_10201-mRNA-1 | |
| **HSF (4)** | HSF_DNA-bind | PF00447.10 | CCMP1779_10669-mRNA-1 | |
|  | HSF_DNA-bind | PF00447.10 | CCMP1779_10518-mRNA-1 | |
|  | HSF_DNA-bind | PF00447.10 | CCMP1779_7703-mRNA-1 | |
|  | HSF_DNA-bind | PF00447.10 | CCMP1779_6661-mRNA-1 | |
| **LFY (1)** | FLO_LFY | PF01698.9 | CCMP1779_7745-mRNA-1 | |
| **mTERF (4)** | mTERF | PF02536.7 | CCMP1779_11895-mRNA-1 | |
|  | mTERF | PF02536.7 | CCMP1779_11964-mRNA-1 | |
|  | mTERF | PF02536.7 | CCMP1779_8337-mRNA-1 | |
|  | mTERF | PF02536.7 | CCMP1779_2960-mRNA-1 | |
| **MYB (17)** | Myb_DNA-binding | PF00249.24 | CCMP1779_5656-mRNA-1 | |
|  | Myb_DNA-binding | PF00249.24 | CCMP1779_9716-mRNA-1 | |
|  | Myb_DNA-binding | PF00249.24 | CCMP1779_7538-mRNA-1 | |
|  | Myb_DNA-binding | PF00249.24 | CCMP1779_5043-mRNA-1 | |
|  | Myb_DNA-binding | PF00249.24 | CCMP1779_10103-mRNA-1 | |
|  | Myb_DNA-binding | PF00249.24 | CCMP1779_6319-mRNA-1 | |
|  | Myb_DNA-binding | PF00249.24 | CCMP1779_10103-mRNA-1 | |
|  | Myb_DNA-binding | PF00249.24 | CCMP1779_7733-mRNA-1 | |
|  | Myb_DNA-binding | PF00249.24 | CCMP1779_6952-mRNA-1 | |
|  | Myb_DNA-binding | PF00249.24 | CCMP1779_11879-mRNA-1 | |
|  | Myb_DNA-binding | PF00249.24 | CCMP1779_3822-mRNA-1 | |
|  | Myb_DNA-binding | PF00249.24 | CCMP1779_5907-mRNA-1 | |
|  | Myb_DNA-binding | PF00249.24 | CCMP1779_3344-mRNA-1 | |
|  | Myb_DNA-binding | PF00249.24 | CCMP1779_11942-mRNA-1 | |
|  | Myb_DNA-binding | PF00249.24 | CCMP1779_7356-mRNA-1 | |
|  | Myb_DNA-binding | PF00249.24 | CCMP1779_2770-mRNA-1 | |
|  | Myb_DNA-binding | PF00249.24 | CCMP1779_6210-mRNA-1 | |
| **MYB-related (12)** | Myb_DNA-binding | PF00249.24 | CCMP1779_5047-mRNA-1 | |
|  | Myb_DNA-binding | PF00249.24 | CCMP1779_10304-mRNA-1 | |
|  | Myb_DNA-binding | PF00249.24 | CCMP1779_5384-mRNA-1 | |
|  | Myb_DNA-binding | PF00249.24 | CCMP1779_2459-mRNA-1 | |
|  | Myb_DNA-binding | PF00249.24 | CCMP1779_3609-mRNA-1 | |
|  | Myb_DNA-binding | PF00249.24 | CCMP1779_6384-mRNA-1 | |
|  | Myb_DNA-binding | PF00249.24 | CCMP1779_2331-mRNA-1 | |
|  | Myb_DNA-binding | PF00249.24 | CCMP1779_11780-mRNA-1 | |
|  | Myb_DNA-binding | PF00249.24 | CCMP1779_6076-mRNA-1 | |
|  | Myb_DNA-binding | PF00249.24 | CCMP1779_9814-mRNA-1 | |
|  | Myb_DNA-binding | PF00249.24 | CCMP1779_3496-mRNA-1 | |
|  | Myb_DNA-binding | PF00249.24 | CCMP1779_2129-mRNA-1 | |
| **Sigma70-like (5)** | Sigma70_r2 & Sigma70_r4 | PF04542.7 | CCMP1779_5163-mRNA-1 | |
|  | Sigma70_r2 | PF04542.7 | CCMP1779_10506-mRNA-1 | |
|  | Sigma70_r2 | PF04542.7 | CCMP1779_8411-mRNA-1 | |
|  | Sigma70_r2 & Sigma70_r4 | PF04542.7 | CCMP1779_5163-mRNA-1 | |
|  | Sigma70_r2 | PF04542.7 | CCMP1779_8114-mRNA-1 | |
|  | Sigma70_r2 & Sigma70_r3 | PF04542.7 | CCMP1779_1521-mRNA-1 | |
| **TAZ (2)** | zf-TAZ | PF02135.9 | CCMP1779_6432-mRNA-1 | |
|  | zf-TAZ | PF02135.9 | CCMP1779_298-mRNA-2 | |
| **TIG (2)** | TIG | PF01833.17 | CCMP1779_11906-mRNA-1 | |
|  | TIG | PF01833.17 | CCMP1779_11619-mRNA-1 | |
| **Tub (3)** | Tub | PF01167.11 | CCMP1779_4350-mRNA-1 | |
|  | Tub | PF01167.11 | CCMP1779_279-mRNA-1 | |
|  | Tub | PF01167.11 | CCMP1779_279-mRNA-1 | |
| **Zn-clus (9)** | Zn_clus | PF00172.11 | CCMP1779_4311-mRNA-1 | |
|  | Zn_clus | PF00172.11 | CCMP1779_2380-mRNA-1 | |
|  | Zn_clus | PF00172.11 | CCMP1779_10368-mRNA-1 | |
|  | Zn_clus | PF00172.11 | CCMP1779_4023-mRNA-1 | |
|  | Zn_clus | PF00172.11 | CCMP1779_747-mRNA-1 | |
|  | Zn_clus | PF00172.11 | CCMP1779_8711-mRNA-1 | |
|  | Zn_clus | PF00172.11 | CCMP1779_8712-mRNA-1 | |
|  | Zn_clus | PF00172.11 | CCMP1779_7006-mRNA-1 | |
|  | Zn_clus | PF00172.11 | CCMP1779_10456-mRNA-1 | |
| **TRANSCRIPTIONAL REGULATORS** | | | | |
| **GNAT (21)** | Acetyltransf_1 | PF00583.17 | | CCMP1779_9273-mRNA-1 |
|  | Acetyltransf_1 | PF00583.17 | | CCMP1779_6048-mRNA-1 |
|  | Acetyltransf_1 | PF00583.17 | | CCMP1779_5286-mRNA-1 |
|  | Acetyltransf_1 | PF00583.17 | | CCMP1779_9314-mRNA-1 |
|  | Acetyltransf_1 | PF00583.17 | | CCMP1779_2709-mRNA-1 |
|  | Acetyltransf_1 | PF00583.17 | | CCMP1779_1641-mRNA-1 |
|  | Acetyltransf_1 | PF00583.17 | | CCMP1779_10759-mRNA-1 |
|  | Acetyltransf_1 | PF00583.17 | | CCMP1779_1663-mRNA-1 |
|  | Acetyltransf_1 | PF00583.17 | | CCMP1779_5094-mRNA-1 |
|  | Acetyltransf_1 | PF00583.17 | | CCMP1779_11751-mRNA-1 |
|  | Acetyltransf_1 | PF00583.17 | | CCMP1779_2433-mRNA-1 |
|  | Acetyltransf_1 | PF00583.17 | | CCMP1779_7749-mRNA-1 |
|  | Acetyltransf_1 | PF00583.17 | | CCMP1779_319-mRNA-1 |
|  | Acetyltransf_1 | PF00583.17 | | CCMP1779_11616-mRNA-1 |
|  | Acetyltransf_1 | PF00583.17 | | CCMP1779_3987-mRNA-1 |
|  | Acetyltransf_1 | PF00583.17 | | CCMP1779_9314-mRNA-1 |
|  | Acetyltransf_1 | PF00583.17 | | CCMP1779_3433-mRNA-1 |
|  | Acetyltransf_1 | PF00583.17 | | CCMP1779_3337-mRNA-1 |
|  | Acetyltransf_1 | PF00583.17 | | CCMP1779_5587-mRNA-1 |
|  | Acetyltransf_1 | PF00583.17 | | CCMP1779_10255-mRNA-1 |
|  | Acetyltransf_1 | PF00583.17 | | CCMP1779_8927-mRNA-1 |
| **HMG (9)** | HMG_box | PF00505.12 | | CCMP1779_4336-mRNA-1 |
|  | HMG_box | PF00505.12 | | CCMP1779_10709-mRNA-1 |
|  | HMG_box | PF00505.12 | | CCMP1779_9684-mRNA-1 |
|  | HMG_box | PF00505.12 | | CCMP1779_10709-mRNA-1 |
|  | HMG_box | PF00505.12 | | CCMP1779_7887-mRNA-1 |
|  | HMG_box | PF00505.12 | | CCMP1779_10477-mRNA-1 |
|  | HMG_box | PF00505.12 | | CCMP1779_8679-mRNA-1 |
|  | HMG_box | PF00505.12 | | CCMP1779_6026-mRNA-1 |
|  | HMG_box | PF00505.12 | | CCMP1779_1523-mRNA-1 |
| **IWS1 (5)** | Med26 | PF08711.4 | | CCMP1779_6574-mRNA-1 |
|  | Med26 | PF08711.4 | | CCMP1779_10080-mRNA-1 |
|  | Med26 | PF08711.4 | | CCMP1779_5876-mRNA-1 |
|  | Med26 | PF08711.4 | | CCMP1779_7879-mRNA-1 |
|  | Med26 | PF08711.4 | | CCMP1779_936-mRNA-1 |
| **Jumonji (7)** | JmjC | PF02373.15 | | CCMP1779_2861-mRNA-1 |
|  | JmjC | PF02373.15 | | CCMP1779_6636-mRNA-1 |
|  | JmjC & JmjN | PF02373.15 | | CCMP1779_1872-mRNA-1 |
|  | JmjC | PF02373.15 | | CCMP1779_2998-mRNA-1 |
|  | JmjC | PF02373.15 | | CCMP1779_3078-mRNA-1 |
|  | JmjC & JmjN | PF02373.15 | | CCMP1779_8742-mRNA-1 |
|  | JmjN | PF02375.10 | | CCMP1779_3082-mRNA-1 |
| **MED6 (1)** | Med6 | PF04934.7 | | CCMP1779_859-mRNA-1 |
| **PHD (14)** | PHD | PF00628.22 | | CCMP1779_7595-mRNA-1 |
|  | PHD | PF00628.22 | | CCMP1779_6575-mRNA-1 |
|  | PHD | PF00628.22 | | CCMP1779_5003-mRNA-1 |
|  | PHD | PF00628.22 | | CCMP1779_4733-mRNA-1 |
|  | PHD | PF00628.22 | | CCMP1779_10758-mRNA-1 |
|  | PHD | PF00628.22 | | CCMP1779_5699-mRNA-1 |
|  | PHD | PF00628.22 | | CCMP1779_3127-mRNA-1 |
|  | PHD | PF00628.22 | | CCMP1779_10304-mRNA-1 |
|  | PHD | PF00628.22 | | CCMP1779_444-mRNA-1 |
|  | PHD | PF00628.22 | | CCMP1779_621-mRNA-1 |
|  | PHD | PF00628.22 | | CCMP1779_1367-mRNA-1 |
|  | PHD | PF00628.22 | | CCMP1779_239-mRNA-1 |
|  | PHD | PF00628.22 | | CCMP1779_34-mRNA-1 |
|  | PHD | PF00628.22 | | CCMP1779_4655-mRNA-1 |
| **Rcd1-like (1)** | Rcd1 | PF04078.6 | | CCMP1779_6941-mRNA-1 |
| **SET (15)** | SET | PF00856.21 | | CCMP1779_9932-mRNA-1 |
|  | SET | PF00856.21 | | CCMP1779_4870-mRNA-1 |
|  | SET | PF00856.21 | | CCMP1779_10404-mRNA-1 |
|  | SET | PF00856.21 | | CCMP1779_9619-mRNA-1 |
|  | SET | PF00856.21 | | CCMP1779_9932-mRNA-1 |
|  | SET | PF00856.21 | | CCMP1779_10920-mRNA-1 |
|  | SET | PF00856.21 | | CCMP1779_7213-mRNA-1 |
|  | SET | PF00856.21 | | CCMP1779_7423-mRNA-1 |
|  | SET | PF00856.21 | | CCMP1779_10920-mRNA-1 |
|  | SET | PF00856.21 | | CCMP1779_620-mRNA-1 |
|  | SET | PF00856.21 | | CCMP1779_7328-mRNA-1 |
|  | SET | PF00856.21 | | CCMP1779_3578-mRNA-1 |
|  | SET | PF00856.21 | | CCMP1779_8516-mRNA-1 |
|  | SET | PF00856.21 | | CCMP1779_2174-mRNA-1 |
|  | SET | PF00856.21 | | CCMP1779_4387-mRNA-1 |
| **SNF2 (27)** | SNF2_N | PF00176.16 | | CCMP1779_5791-mRNA-1 |
|  | SNF2_N | PF00176.16 | | CCMP1779_5046-mRNA-1 |
|  | SNF2_N | PF00176.16 | | CCMP1779_386-mRNA-1 |
|  | SNF2_N | PF00176.16 | | CCMP1779_7577-mRNA-1 |
|  | SNF2_N | PF00176.16 | | CCMP1779_7826-mRNA-1 |
|  | SNF2_N | PF00176.16 | | CCMP1779_4163-mRNA-1 |
|  | SNF2_N | PF00176.16 | | CCMP1779_558-mRNA-1 |
|  | SNF2_N | PF00176.16 | | CCMP1779_5892-mRNA-1 |
|  | SNF2_N | PF00176.16 | | CCMP1779_8560-mRNA-1 |
|  | SNF2_N | PF00176.16 | | CCMP1779_10795-mRNA-1 |
|  | SNF2_N | PF00176.16 | | augustus_masked-nanno_727-abinit-gene-0.12-mRNA-1 ^1^ |
|  | SNF2_N | PF00176.16 | | CCMP1779_2033-mRNA-1 |
|  | SNF2_N | PF00176.16 | | CCMP1779_4550-mRNA-1 |
|  | SNF2_N | PF00176.16 | | CCMP1779_3081-mRNA-1 |
|  | SNF2_N | PF00176.16 | | CCMP1779_5237-mRNA-1 |
|  | SNF2_N | PF00176.16 | | CCMP1779_9684-mRNA-1 |
|  | SNF2_N | PF00176.16 | | CCMP1779_4234-mRNA-1 |
|  | SNF2_N | PF00176.16 | | CCMP1779_7254-mRNA-1 |
|  | SNF2_N | PF00176.16 | | CCMP1779_9976-mRNA-1 |
|  | SNF2_N | PF00176.16 | | CCMP1779_9187-mRNA-1 |
|  | SNF2_N | PF00176.16 | | CCMP1779_11503-mRNA-1 |
|  | SNF2_N | PF00176.16 | | CCMP1779_1832-mRNA-1 |
|  | SNF2_N | PF00176.16 | | CCMP1779_2443-mRNA-1 |
|  | SNF2_N | PF00176.16 | | CCMP1779_9187-mRNA-1 |
|  | SNF2_N | PF00176.16 | | CCMP1779_9187-mRNA-1 |
|  | SNF2_N | PF00176.16 | | CCMP1779_4210-mRNA-1 |
|  | SNF2_N | PF00176.16 | | CCMP1779_241-mRNA-1 |
| **SOH1 (2)** | Med31 | PF05669.5 | | CCMP1779_2467-mRNA-1 |
|  | Med31 | PF05669.5 | | CCMP1779_2467-mRNA-1 |
| **SWI/SNF-BAF60b (2)** | SWIB | PF02201.11 | | CCMP1779_5044-mRNA-1 |
|  | SWIB | PF02201.11 | | CCMP1779_10286-mRNA-1 |
| **SWI/SNF-SWI3 (1)** | SWIRM | PF04433.10 | | CCMP1779_9651-mRNA-1 |
| **TRAF (4)** | BTB | PF00651.24 | | CCMP1779_6768-mRNA-1 |
|  | BTB | PF00651.24 | | CCMP1779_7977-mRNA-1 |
|  | BTB | PF00651.24 | | CCMP1779_1712-mRNA-1 |
|  | BTB | PF00651.24 | | CCMP1779_9947-mRNA-1 |

^1^ this gene model is from augustus or snap gene annotation and was found superior to the final maker annotation after manual examination
