# Supplementary material for: Genome, Functional Gene Annotation, and Nuclear Transformation of the Heterokont Oleaginous Alga Nannochloropsis oceanica CCMP1779
Source: PLoS Genet. 2012 Nov 15;8(11):e1003064. doi: 10.1371/journal.pgen.1003064 (PMC3499364; doi:10.1371/journal.pgen.1003064)
Supplement: Text S1 — Supplemental results and discussion. Additional annotation is provided for genes predicted to be involved in ROS scavenging systems, oxidative phosphorylation, amino acid biosynthesis, degradation of branched chain amino acids, sulfate uptake and metabolism, and histones and histone variants. (DOC) [file pgen.1003064.s039.doc]

**SUPPLEMENTAL RESULTS AND DISCUSSION**

**ROS scavenging systems**

Photosynthetic organisms including plants and microalgae are commonly challenged by biotic and abiotic stresses, which are often accompanied by the production of reactive oxygen species (ROSs) and a loss in photosynthesis efficiency . Thus, there is a need for an efficient ROS scavenging system in photosynthetic cells. It involves the scavenging of hydroxyl radicals through an activation of enzymatic and nonenzymatic mechanisms. Cellular redox buffers such as ascorbate and glutathione (GSH) serve as antioxidants in plants. Glutathione is an abundant compound, found virtually in all cell compartments, which reacts non-enzymatically with cytotoxic hydroxyl radicals and H_2_O_2_. Through the ascorbate-glutathione cycle, GSH generates another powerful antioxidant, ascorbic acid . The CCMP1779 genome contains a gene for glutathione synthase (GSH) and microsomal glutathione S-transferase (mGSH), which could participate in ascorbate-glutathione mediated ROS scavenging. Enzymatic ROS scavenging mechanisms include superoxide dismutase (SOD), ascorbate peroxidase (APX), glutathione peroxidase (GPX), and catalase (CAT) (Table S11). The CCMP1779 genome has genes likely encoding SOD and GPX that might participate in enzymatic ROS scavenging pathways. Thus, CCMP1779 likely shares basic stress responses with flowering plants.

**Oxidative phosphorylation**

Organisms rely on the reduction of O_2_ to H_2_O to drive mitochondrial ATP synthesis during aerobic metabolism , e.g. algae during the night. Genes were identified which encode key subunits of NADH dehydrogenase (complex I), succinate dehydrogenase (complex II), cytochrome *c* reductase (complex III), cytochrome *c* oxidase (complex IV), and F_o_F_1_ ATP synthase (complex V), as well as numerous accessory proteins required for the proper folding and the insertion of cofactors into these protein complexes (Table S11). In addition, transcript analysis identified ESTs coding for respiratory proteins canonically coded by mitochondrial DNA, which was not sequenced. Finally, three genes were found that encode putative alternative oxidases, proteins which permit the reduction of O_2_ without the concomitant synthesis of ATP . Together, the presence of these genes strongly implies a typical respiratory electron transfer chain used to couple O_2_ reduction to ATP synthesis.

### Amino Acid Biosynthesis

CCMP1779 is predicted to synthesize Lys, Met and Thr from Asp. There are two branch-point metabolites in the pathway, Asp semialdehyde and homoserine 4-phosphate (Figure S4). Asp semialdehyde is the substrate for branch-point enzymes dihydrodipicolinate synthase (DHDPS) and homoserine dehydrogenase (HSDH). These two enzymes control the flux between Lys and the precursor for Met and Thr. Homoserine 4-phosphate is the substrate for branch-point enzymes cystathionine gamma synthase (CGS) and Thr synthase (TS). These two enzymes control the metabolic flow between Met and Thr. In Arabidopsis, there are three mono-functional Asp kinases (AK), one mono-functional HSDH, and two dual-functional AK-HSDHs (Figure S4). Based on BLAST similarity, PFAM domain support, and reverse BLAST analysis, we predict that CCMP1779 has one AK, one HSDH and one AK-HSDH (Figure S4 and Table S16). AK-HSDH fusion is also present in other organisms, such as the bacterium *Escherichia coli*, green alga *Chlamydomonas reinhardtii*, diatoms *Phaeodactylum tricornutum* and *Thalassiosira pseudonana*, and seed plant Arabidopsis, but it is not found in cyanobacterium Synechocystis *sp* (Figure S5 and Table S17). It should be noted that the putative dihydrodipicolinate reductase (DHDPR), diaminopimelate epimerase (DAPE) encoding genes and the two Thr synthase (TS) encoding genes appear to be partial in the CCMP1779 genome (Figure S4 and Table S16). The DHDPR and DAPE encoding genes are missing the 5’-ends of the coding regions (Table S16). In CCMP1779, there are five putative genes encoding CGS or cystathionine beta lyase (CBL) (Figure S4 and Table S16). Overall, CCMP1779 has all the genes for Asp-derived amino acid biosynthesis. One interesting difference between CCMP1779 and Arabidopsis is that CCMP1779 has two predicted cobalamin-independent Met synthases (MS) and one cobalamin-dependent Met synthase (MetH); Arabidopsis has three cobalamin-independent Met synthases, but no cobalamin-dependent Met synthase (Figure S4 and Table S16).

Similar to Asp-derived amino acid, the biosynthesis of aromatic amino acids also has two branch-point metabolites: chorismate and arogenate. Chorismate is the substrate for chorismate mutase (CM), the committing (or branch-point) enzyme for Phe and Tyr biosynthesis, and anthranilate synthase (ASA and ASB), the committing enzyme for Trp biosynthesis. Arogenate is the substrate for arogenate dehydratase (ADT), the committing enzyme for Phe biosynthesis, and arogenate dehydrogenase (ADH), the committing enzyme for Tyr biosynthesis. Aromatic amino acid biosynthesis is one of the most interesting pathways in CCMP1779 because five gene pairs in this pathway are predicted as fusion genes, including those encoding dehydroquinate dehydratase and shikimate dehydrogenase (DHQDH-SDH), arogenate dehydratase and arogenate dehydrogenase (ADT-ADH), anthranilate synthase alpha and beta subunits (ASA-ASB), indole-3-glycerol phosphate synthase and phosphoribosylanthranilate isomerase (IGPS-PAI), and Trp synthase alpha and beta subunits (TSA-TSB) (Figure S5). These five fusion gene pairs are also present in diatoms *P. tricornutum* and *T. pseudonana* (Table S17). It was recently proposed that the ancestral state of Trp biosynthesis is composed of separate ASA and ASB, fusion of IGPS and PAI, and fusion of TSA and TSB, and that ASA-ASB fusion in diatoms is the result of an ancient replacement of separate ASA and ASB by fused bacterial ASA-ASB . Even though some of the open reading frames are incomplete, CCMP1779 appears to contain all the necessary genes for aromatic amino acid biosynthesis.

CCMP1779 also appears to synthesize branched-chain amino acids (BCAAs) by a common pathway starting with two molecules of pyruvate to form one molecule of Val or Leu, and one molecule of pyruvate and one molecule of Thr-derived 2-oxobutanotate for Ile (Figure S4). An interesting exception is that some prokaryotes use Thr-independent citramalate pathway to form Ile from pyruvate and acetyl-CoA . A unique feature of BCAA biosynthesis is that Val and Ile are synthesized in two parallel pathways (Figure S4). They share a set of four enzymes by using different substrates, and those enzymes include AHAS, ketolacid reductoisomerase (KARI), dihydroxyacid dehydratase (DHAD) and branched-chain aminotransferase (BCAT) . The biosynthesis of Leu branches off from the last intermediate, 2-keto-isovalerate, in Val biosynthesis. Leu biosynthetic enzymes include isopropylmalate synthase (IPMS), isopropylmalate isomerase (IPMI), isopropylmalate dehydrogenase (IMDH), and BCAT . Four genes encoding IPMS-like proteins are present in Arabidopsis, two of them encode true IPMS (Figure S4) and the other two encode methylthioalkylmalate synthases (MAM), which control the Met-derived aliphatic glucosinolate side-chain elongation process . In prokaryotes, algae, and plants, the IPMI enzyme is a heterodimer composed of a large (IPMIL) and a small subunit (IPMIS) . CCMP1779 appears to have one copy of large subunit and one copy of small subunit (Figure S4 and Table S16). Aside from the fact that some open reading frames are incomplete, CCMP1779 appears to have all the genes necessary for BCAA biosynthesis.

**Degradation of branched chain amino acids**

The degradation of BCAAs occurs in mitochondria. In animals, impaired breakdown leads to several severe diseases like maple syrup urine disease, which is caused by disrupted branched-chain keto-acid dehydrogenase (BCKDH), with symptoms like a buildup of BCAAs and their toxic by-products in the blood and urine in the patients . Plants have the ability to degrade BCAAs like animals. Similarly, most of the genes involved in the degradation of BCAAs in plants are found to be targeted to mitochondria, but evidence has shown that the later steps in Val degradation might occur in peroxisomes . Several Arabidopsis mutants that have defective enzymes in Leu degradation have been characterized to show a pleiotropic seed amino acid phenotype leading by high accumulation of three BCAAs . BCAA degradation is still under intensive study and the genes for all enzymes have yet to be discovered. Several confirmed Arabidopsis genes were selected for analysis in CCMP1779 and it was possible to find corresponding orthologs for each. All were single copy except for isovaleryl-CoA dehydrogenase (IVD) which is encoded by a single locus in Arabidopsis but has two gene models with high similarity in CCMP1779. However, whether the two IVDs are true or not still needs to be confirmed experimentally.

### Sulfate uptake and metabolism

At least eight distinct genes encoding eukaryotic-type sulfate transporters were present in CCMP1779 (Table S18). The membranous proteins annotated as putative sulfate transporters are predicted to be structurally and functionally similar with plant SULTR and yeast SUL gene family members . By contrast, the sulfate transport and sensing mechanisms were suggested to be different from those in green algae. The SAC1/SLT family proteins known as putative sodium/sulfate transporters and sulfur-sensory protein of Chlamydomonas , and the components for a prokaryotic-type sulfate permease complex that may facilitate sulfate transport into algal plastids , were both absent from the CCMP1779 genome.

Subsequent to the uptake of sulfate, ATP sulfurylase (ATPS) catalyzes the synthesis adenosine 5’-phosphosulfate (APS) from sulfate and ATP . In CCMP1779, two genes encoding ATPS were present: one was predicted to encode a plastid-localized ATPS as in plants, while the other was predicted to encode a fusion protein of APS kinase (APK) and ATPS with an attachment of a pyrophosphatase domain at its *C*-terminus (Figure S6A). The latter gene model potentially encodes a 3’-phosphoadenosine 5’-phosphosulfate (PAPS) synthetase. The arrangement of APK and ATPS catalytic domains into one fusion protein appears to be conserved in heterokonts, metazoans and choanozoans , and it also resembles the organization of bacterial orthologs encoded by the *cysDNC* operon . The role of the *C*-terminal pyrophosphatase domain of heterokont and CCMP1779 putative PAPS synthetase remains uninvestigated, although it appears to be an essential component similar to GTPase (*cysN*) which drives the energetically unfavorable reaction of ATPS (*cysD*) in bacterial cells . For the subsequent APS reduction step, APR-B-type APS reductase was predicted to be the responsible enzyme in CCMP1779 (Figure S6B). APR-B is known as a variant of PAPS reductase but is capable of catalyzing the reduction of APS rather than PAPS , and is considered to be a form adapted to low-iron conditions as it does not require Fe-S cluster as a prosthetic group . APR-B was first reported from bryophyte *Physcomitrella patens* and is the enzyme present in bryophytes, lycophytes and marine algae . A more recent study suggested that the acidic residue in the P-loop region is essential for the APS reducing activity of APR-B . Consequently, the APR-B-type enzyme is likely the only sulfonucleotide reductase in CCMP1779, and the putative PAPS synthetase mentioned above is most likely responsible for phosphorylation of APS (Figure S6B). Therefore, it is reasonable to hypothesize that APS reduction and phosphorylation pathways are compartmentalized in the plastid and cytosol, requiring enzymes specialized for the respective reactions (Figure S6A).

Sulfite is reduced to sulfide by sulfite reductase . In plants, sulfide is used for synthesis of Cys , while in yeast it primarily serves as a substrate for homocysteine biosynthesis . We found gene models encoding the enzymes for both pathways in CCMP1779 (Table S18; Figure S6C). Thus, the S-amino acid biosynthetic pathways appear to be bipartite utilizing either *O*-acetylserine or *O*-acetylhomoserine as precursor carbon skeletons (Figure S6C). Genes encoding cystathionine synthesis and metabolism, and Met and *S*-adenosylmethionine biosynthetic/recycling pathways were also identified in CCMP1779. Within the Met recycling pathway, *S*-adenosylhomocysteine hydrolase (SAHH) was suggested to be a multi-domain fusion protein containing aspartate aminotransferase domain attached to its *N*-terminus (Table S18).

Biosynthesis of glutathione (GSH) occurs in two consecutive reactions catalyzed by γ-glutamylcysteine synthetase and GSH synthetase (Figure S6C). Potential orthologs encoding both enzymes were identified in the CCMP1779 genome; however a sequence similar to γ-glutamylcysteine synthetase only encoded the *N*-terminal portion of the coding region which leaves the remaining part of the enzyme yet to be identified. In addition to the biosynthetic pathways, GSH conjugating and recycling enzymes were also encoded in the CCMP1779 genome (Table S18).

### Histones and histone variants

Eukaryotic genomes are maintained in a compacted chromatin state through the presence of nucleosomes . Nucleosomes are composed of octomers of four histone cores (H2A, H2B, H3, and H4) around which the DNA double helix is wrapped . Modifications, such as acetylation and phosphorylation, on these histone cores can also be used to alter gene expression or protein binding . In addition to these modifications, variant histones can also be utilized within the nucleosome to alter the behavior of the chromatin . Like many eukaryotes, CCMP1779 contains multiple copies of each canonical histone gene, as well as at least one histone variant, histone H2A.Z. The H2A.Z variant is associated with regulation of gene expression as well as DNA repair and chromosomal segregation . The presence of this specialized variant suggests Nannochloropsis utilizes the same alterations of nucleosome components as other eukaryotes. In addition, copies of H2A, H3, and H4 encoding genes were found in close proximity on the same contig, suggesting transcription of these genes could be linked. These findings are consistent with eukaryotic histones in general. Nannochloropsis utilizes the core histones (H2A, H2B, H3, and H4) as well as key variants (H2A.Z) to maintain proper cell function.

**SUPPLEMENTAL REFERENCES**

1. Apel K, Hirt H (2004) REACTIVE OXYGEN SPECIES: Metabolism, Oxidative Stress, and Signal Transduction. Annual Review of Plant Biology 55: 373-399.

2. Dittami SM, Scornet D, Petit JL, Segurens B, Da Silva C, et al. (2009) Global expression analysis of the brown alga Ectocarpus siliculosus (Phaeophyceae) reveals large-scale reprogramming of the transcriptome in response to abiotic stress. Genome Biol 10: R66.

3. Hosler JP, Ferguson-Miller S, Mills DA (2006) Energy Transduction: Proton Transfer Through the Respiratory Complexes. Annual Review of Biochemistry 75: 165-187.

4. Gupta KJ, Zabalza A, Van Dongen JT (2009) Regulation of respiration when the oxygen availability changes. Physiologia Plantarum 137: 383-391.

5. Jiroutova K, Horak A, Bowler C, Oborn+­k M (2007) Tryptophan Biosynthesis in Stramenopiles: Eukaryotic Winners in the Diatom Complex Chloroplast. Journal of Molecular Evolution 65: 496-511.

6. Akman Gndz E, Douglas AE (2009) Symbiotic bacteria enable insect to use a nutritionally inadequate diet. Proceedings of the Royal Society B: Biological Sciences 276: 987-991.

7. Binder S (2010) Branched-Chain Amino Acid Metabolism in Arabidopsis thaliana. The Arabidopsis Book: e0137.

8. Singh BK, Shaner DL (1995) Biosynthesis of Branched Chain Amino Acids: From Test Tube to Field. The Plant Cell Online 7: 935-944.

9. de Kraker JW, Luck K, Textor S, Tokuhisa JG, Gershenzon J (2007) Two Arabidopsis Genes (IPMS1 and IPMS2) Encode Isopropylmalate Synthase, the Branchpoint Step in the Biosynthesis of Leucine. Plant Physiology 143: 970-986.

10. Textor S, de Kraker JW, Hause B, Gershenzon J, Tokuhisa JG (2007) MAM3 Catalyzes the Formation of All Aliphatic Glucosinolate Chain Lengths in Arabidopsis. Plant Physiology 144: 60-71.

11. Chuang DT, Chuang JL, Wynn RM (2006) Lessons from Genetic Disorders of Branched-Chain Amino Acid Metabolism. The Journal of Nutrition 136: 243S-249S.

12. Lange PR, Eastmond PJ, Madagan K, Graham IA (2004) An Arabidopsis mutant disrupted in valine catabolism is also compromised in peroxisomal fatty acid ƒ-oxidation. FEBS Lett 571: 147-153.

13. Lu Y, Savage LJ, Larson MD, Wilkerson CG, Last RL (2011) Chloroplast 2010: A Database for Large-Scale Phenotypic Screening of Arabidopsis Mutants. Plant Physiology 155: 1589-1600.

14. Gu L, Jones AD, Last RL (2010) Broad connections in the Arabidopsis seed metabolic network revealed by metabolite profiling of an amino acid catabolism mutant. The Plant Journal 61: 579-590.

15. Takahashi H, Kopriva S, Giordano M, Saito K, Hell R (2011) Sulfur Assimilation in Photosynthetic Organisms: Molecular Functions and Regulations of Transporters and Assimilatory Enzymes. Annual Review of Plant Biology 62: 157-184.

16. Pootakham W, Gonzalez-Ballester D, Grossman AR (2010) Identification and regulation of plasma membrane sulfate transporters in Chlamydomonas. Plant Physiol 153: 1653-1668.

17. Lindberg P, Melis A (2008) The chloroplast sulfate transport system in the green alga Chlamydomonas reinhardtii. Planta 228: 951-961.

18. Patron N, Durnford D, Kopriva S (2008) Sulfate assimilation in eukaryotes: fusions, relocations and lateral transfers. BMC Evolutionary Biology 8: 39.

19. Mougous JD, Lee DH, Hubbard SC, Schelle MW, Vocadlo DJ, et al. (2006) Molecular Basis for G Protein Control of the Prokaryotic ATP Sulfurylase. Molecular Cell 21: 109-122.

20. Kopriva S, Fritzemeier K, Wiedemann G, Reski R (2007) The putative moss 3' phosphoadenosine 5' phosphosulfate reductase is a novel form of adenosine 5' phosphosulfate reductase without an iron sulfur cluster. Journal of Biological Chemistry: M702522200.

21. Bhave DP, Hong JA, Keller RL, Krebs C, Carroll KS (2012) Iron sulfur cluster engineering provides insight into the evolution of substrate specificity among sulfonucleotide reductases. ACS Chemical Biology 7: 306-315.

22. Thomas D, Surdin-Kerjan Y (1997) Metabolism of sulfur amino acids in Saccharomyces cerevisiae. Microbiology and Molecular Biology Reviews 61: 503-532.

23. Kornberg RD, Thonmas JO (1974) Chromatin Structure: Oligomers of the Histones. Science 184: 865-868.

24. Luger K, Mader AW, Richmond RK, Sargent DF, Richmond TJ (1997) Crystal structure of the nucleosome core particle at 2.8 A resolution. Nature 389: 251-260.

25. Kouzarides T (2007) Chromatin modifications and their function. Cell 128: 693-705.

26. Bannister AJ, Kouzarides T (2011) Regulation of chromatin by histone modifications. Cell Res 21: 381-395.

27. Henikoff S, Ahmad K (2005) Assembly of variant histones into chromatin Annual Review of Cell and Developmental Biology 21: 133-153.

28. Hake SB, Allis CD (2006) Histone H3 variants and their potential role in indexing mammalian genomes: The "H3 barcode hypothesis". Proceedings of the National Academy of Sciences 103: 6428-6435.

29. Zlatanova J, Thakar A (2008) H2A.Z: View from the Top. Structure (London, England : 1993) 16: 166-179.
