# Supplementary figures and images for: Correction: Genome, Functional Gene Annotation, and Nuclear Transformation of the Heterokont Oleaginous Alga Nannochloropsis oceanica CCMP1779
Source: PLoS Genet. 2017 May 23;13(5):e1006802. doi: 10.1371/journal.pgen.1006802 (PMC5441573; doi:10.1371/journal.pgen.1006802)

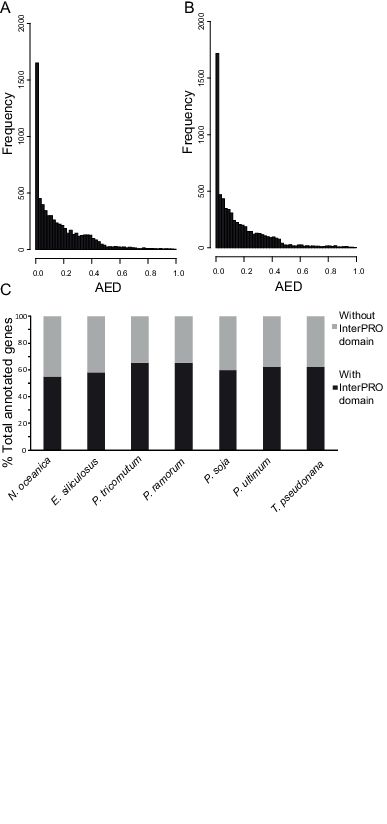

Supplement: S2 Fig — (A) Annotation Edit Distance (AED) distribution of gene models in the first annotation set after eliminating entries with AED = 1. (B). AED distribution of gene models in the second annotation after eliminating entries with AED = 1. (C) Proportion of gene models with protein domain hits in different heterokonts (abbreviated as indicated in Materials and Methods). (JPG) [file pgen.1006802.s001.jpg]
